# Global, regional, and national burden of asthma and its attributable risk factors from 1990 to 2019: a systematic analysis for the Global Burden of Disease Study 2019

**DOI:** 10.1186/s12931-023-02475-6

**Published:** 2023-06-23

**Authors:** Zhufeng Wang, Yun Li, Yi Gao, Yu Fu, Junfeng Lin, Xuedong Lei, Jinping Zheng, Mei Jiang

**Affiliations:** 1 National Clinical Research Center for Respiratory Disease, State Key Laboratory of Respiratory Disease, Guangzhou Institute of Respiratory Health, The First Affiliated Hospital of Guangzhou Medical University, 151 Yanjiang Road, Guangzhou, 510120 Guangdong China; 2 Department of Allergy and Clinical Immunology, National Clinical Research Center for Respiratory Disease, State Key Laboratory of Respiratory Disease, Guangzhou Institute of Respiratory Health, The First Affiliated Hospital of Guangzhou Medical University, Guangzhou, Guangdong China

**Keywords:** Asthma, Burden, Risk factors, Age-period-cohort analysis

## Abstract

**Background:**

The burden of asthma in terms of premature death or reduced quality of life remains a huge issue. It is of great importance to evaluate asthma burden geographically and time trends from 1990 to 2019 and to assess the contributions of age, period, and cohort effects at global level.

**Methods:**

Asthma prevalence, deaths, and disability adjusted life years (DALYs) as well as risk-attributable burden were collected from the Global Burden of Diseases, Injuries, and Risk Factors Study (GBD) 2019 database and were compared by age and sex. The Smoothing Splines models were used to estimate the relationship between asthma DALYs and the sociodemographic index (SDI). The Age-Period-Cohort model was used to determine effects of ages, periods, and birth cohorts on disease rates.

**Results:**

Between 1990 and 2019, the declines were 24.05% (95% uncertainty interval [UI] − 27.24 to − 20.82) in age-standardized asthma prevalence, 51.3% (− 59.08 to − 43.71) in mortality, and 42.55% (− 48.48 to − 36.61) in DALYs rate. However, the burden of asthma continued to rise, with an estimated 262.41 million prevalent cases globally (95% UI 224.05 to 309.45). Asthma caused greater DALYs in females than in males among people aged 20 years and older. The lowest age-standardized DALYs rate was observed at a SDI of approximately 0.70. The Longitudinal age curves showed an approximate W-shaped pattern for asthma prevalence and a likely J-shaped pattern for asthma mortality. The period effect on prevalence and mortality of asthma decreased from 1990 to 2019. Compared with the 1955–1959 birth cohort, the prevalence relative risk (RR) of asthma was highest in the 1905–1909 birth cohort, whereas the mortality RR continued to decline. At the global level, the percentages of high body-mass index, occupational asthmagens, and smoking contributing to DALYs due to asthma were 16.94%, 8.82%, and 9.87%, respectively.

**Conclusions:**

Although the age-standardized rates of asthma burden declined in the past 30 years, the overall burden of asthma remains severe. High body mass index becomes the most important risk factor for DALYs due to asthma at the global level.

**Supplementary Information:**

The online version contains supplementary material available at 10.1186/s12931-023-02475-6.

## Background

Asthma is a prevalent chronic inflammatory airway disease marked by airflow limitation, airway hyperresponsiveness, and structural changes in the airways [1]. Despite efforts to address its impact, the Global Initiative for Asthma (GINA) revealed that the burden of asthma in terms of premature death and reduced quality of life remained a significant public health challenge, with economic consequences [2]. A Canadian study found that the financial burden was associated with productivity losses, and the cost of biologic treatments for uncontrolled asthma was high and even greater for severe uncontrolled asthma [3].

The total burden of chronic respiratory diseases was estimated in previous studies by the Chronic Respiratory Disease Collaborators and Li X et al. based on the *Global Burden of Diseases, Injuries, and Risk Factors Study (GBD)* 2015 and 2017 [4–6]. With updated data, the GBD 2019 provides a tool to better quantify health loss and risk-attributable burden. Researchers used the GBD 2019 to investigate chronic respiratory diseases mortality in BRICS (Brazil, Russia, India, China, and South Africa) countries and the burden of chronic respiratory disease and attributable risk factors in North Africa and Middle East, and the burden of childhood asthma [7–9]. Recently, some studies reported the burden of asthma in China using the GBD 2019 [10, 11]. However, there remains a need for more comprehensive comparisons using common indicators such as prevalence, deaths, and disability adjusted life years (DALYs) across countries and regions. Moreover, analyzing the contributions of age, period, and cohort effects at the global level is also necessary.

Asthma management varied in different countries based on their development capacities, with varying priorities. In some African countries, the main challenge was providing effective medicines to asthma patients [12, 13]. In certain Asian countries, strengthening the interpretation of asthma guidelines is a priority [14]. In contrast, in some Latin American countries, improving asthma patients’ self-management education could enhance treatment efficiency [15].

Asthma affects people of all ages, regardless of the region or country. Referring to the Institute for Health Metrics and Evaluation (IHME) in 2010, global asthma DALYs followed a bimodal distribution, with peak values appearing in age groups 10–14 years and 75–79 years, and a trough value in the age group of 30–34 years. Among individuals younger than 30 years, the burden of DALYs was similar for males and females, but the burden was higher in males with age [16]. Recent study by the Global Asthma Network Phase one (GAN I) estimated trends in asthma prevalence and severity of symptoms in children aged 6–7 years and adolescents aged 13–14 years [17]. However, no comprehensive research was conducted to update the global, regional, and national burden of asthma across all ages and stratified by sex in the decade after 2010 [16]. The changing characteristics of asthma burden and the impact of macro-environmental factors on asthma burden remain unknown.

In addition to accurately estimating the disease burden, it is essential to report the risk-attributable burden of asthma caused by the well-recognized risk factors, such as high body-mass index, smoking, and occupational asthmagens. This information can assist in making policies related to public health [18]. For instance, the World Health Organization suggested that tobacco control could directly reduce the risk of asthma, and making adjustments to diet or engaging in physical exercise could reduce obesity and thus lower the risk of asthma [16]. However, the asthma burden attributable to each risk factor is not well defined, particularly in the case of high body-mass index.

This study aimed to provide a comprehensive update on the burden of asthma from multiple angles to provide valuable evidence for future research and policymaking in asthma.

## Methods

### Aims

The aim of this study was to provide updated estimates of asthma prevalence, deaths, and DALYs based on data from the GBD 2019 database, to assess trends from 1990 to 2019, and compared estimates across age and sex groups. The relationship between asthma DALYs and the sociodemographic index (SDI) was analyzed. The contributions of age, period, and cohort effects on the trends of asthma prevalence and mortality were reported. The risk-attributable burden of asthma caused by high body-mass index, smoking, and occupational asthmagens was also estimated.

### Overview and data source

The GBD database provides a tool to quantify health loss from hundreds of diseases, injuries, and risk factors [18, 19]. For this study, the data on asthma prevalence, deaths, and DALYs were derived from the Global Health Data Exchange (GHDx) (https://vizhub.healthdata.org/gbd-results/). In GBD 2019, asthma is a chronic lung disease marked by spasms in the bronchi usually resulting from an allergic reaction or hypersensitivity and causing difficulty in breathing. It is defined asthma as a doctor’s diagnosis and wheezing in the past year. The relevant International Classification of Disease and Injuries (ICD) -10 codes are J45 and J46 (ICD-9 code is 493). Alternative case definitions include self-reported asthma in the past year, self-reported asthma ever, only a doctor’s diagnosis in the past year and only wheezing in the past year. The last full systemic review of the literature on asthma was done for GBD 2016 and data in literature matching the case definitions above were extracted. Some new data from surveys were added. Vital registration and surveillance data from the cause of death (COD) database were used to estimate asthma mortality. The detaied case definition and sources of data for asthma are available in Additional file 1: Table S1.

The SDI is a composite indicator of development status. It is the geometric mean of 0 to 1 indices of total fertility rate under the age of 25, mean education for those ages 15 and older, and lag distributed income per capita. A location with an SDI of 0 has a theoretical minimum level of development relevant to health, while a location with an SDI of 1 has a theoretical maximum level [19]. The SDI values are available from GHDx (https://ghdx.healthdata.org/record/ihme-data/gbd-2019-socio-demographic-index-sdi-1950-2019).

The percentage of asthma DALYs attributable to risk factors, including smoking, high body-mass index, and occupational asthmagens was also obtained from the GHDx. The definition of these factors and their relative risk (RR) for asthma are previously reported [18]. Details are available in Additional file 1: Table S1.

### Statistical analyses

The main modeling tool for asthma is called DisMod-MR version 2.1. Prior settings include a maximum remission of 0.3 (reflecting the upper bound of the highest observed data) and an incidence between the ages of 0 and 0.5 years, as a diagnosis can not be made in young infants. Covariates that are associated with measures of asthma epidemiology in prior studies and for which estimates of those covariates are available for all GBD year-age-sex-location combinations are included in the DisMod model. The standard Cause of Death Ensemble modelling (CODEm) approach was applied to estimate deaths due to asthma. Verbal autopsy data were not included and were instead mapped to an overall chronic respiratory model. The unadjusted death estimates for asthma are combined with those for chronic obstructive pulmonary disease, interstitial lung disease and pulmonary sarcoidosis, pneumoconiosis, and other chronic respiratory diseases and fit to the distribution of deaths in an overall chronic respiratory disease “parent” model and redistributed to the “child” model proportionately. For all results, the 95% uncertainty (combined from input data, corrections of measurement error, estimates of residual non-sampling error, and so on) intervals were reported.

Results at the global and regional levels were compared by age and sex. The relationship between asthma burden and SDI for the 21 regions and 204 countries and territories was examined using Smoothing Splines models. Age-Period-Cohort analysis (https://analysistools.cancer.gov/apc) was used to investigate the influence of age, period, and cohort effects on trends of prevalence and mortality in asthma [20]. Data were analyzed with R version 4.0.5 (http://CRAN.R-project.org, R Foundation, Vienna, Austria).

## Results

### Global level

In 2019, a total of 262.41 (95% UI 224.05 to 309.45) million prevalent cases were estimated, with an age-standardized prevalence of 3415.53 (95% UI 2898.92 to 4066.2) per 100,000, a decrease of 24.05% (20.82 to 27.24%) since 1990. Death cases accounted for asthma were estimated to be 461.07 (95% UI 366.58 to 559.01) thousand, with an age-standardized mortality of 5.8 (95% UI 4.62 to 7.03) per 100,000, a decrease of 51.30% (43.71 to 59.08%) since 1990. The number of DALYs for asthma was 21.55 (95% UI 17.14 to 26.97) million, with an age-standardized DALYs rate of 273.63 (95% UI 216.71 to 343.38) per 100,000, a decrease of 42.55% (36.61 to 48.48%) since 1990 (Table 1).

**Table 1:** Prevalence, deaths, and disability adjusted life years (DALYs) for asthma in 2019, and percentage change in age-standardized rates (ASRs) per 100,000, by Global Burden of Disease region, from 1990 to 2019

	Prevalence (95% UI)			Deaths (95% UI)			DALYs (95% UI)		
	No, in million (95% UI)	ASRs per 100 000 (95% UI)	Percentage change in ASRs from 1990 to 2019	No, in thousand (95% UI)	ASRs per 100 000 (95% UI)	Percentage change in ASRs from 1990 to 2019	No, in thousand (95% UI)	ASRs per 100 000 (95% UI)	Percentage change in ASRs from 1990 to 2019
Global	262.41 (224.05 to 309.45)	3415.53 (2898.92 to 4066.2)	− 24.05 (− 27.24 to − 20.82)	461.07 (366.58 to 559.01)	5.8 (4.62 to 7.03)	− 51.3 (− 59.08 to − 43.71)	21.55 (17.14 to 26.97)*	273.63 (216.71 to 343.38)	− 42.55 (− 48.48 to − 36.61)
Central Asia	2.07 (1.69 to 2.56)	2277.44 (1883.33 to 2787.8)	− 15.38 (− 21.81 to − 9.43)	3.34 (2.87 to 4.05)	5.42 (4.69 to 6.54)	− 47.15 (− 58.05 to − 28.69)	160.74 (126.6 to 204.69)	197.95 (160.01 to 246.02)	− 38.94 (− 48.1 to − 27.36)
Central Europe	5.07 (4.22 to 6.05)	4203.57 (3452.76 to 5155.48)	− 27.45 (− 32.94 to − 21.91)	1.78 (1.51 to 2.14)	0.81 (0.69 to 0.98)	− 82.49 (− 85.25 to − 78.88)	226.33 (157.24 to 318.45)	180.45 (119.90 to 263.92)	− 43.2 (− 50.46 to − 36.75)
Eastern Europe	5.36 (4.38 to 6.46)	2712.03 (2160.78 to 3412.64)	− 42.64 (− 48.15 to − 37.08)	2.33 (1.96 to 3.41)	0.70 (0.59 to 1.02)	− 83.43 (− 86.35 to − 72.96)	260.77 (184.39 to 363.88)	124.09 (84.15 to 182.59)	− 56.99 (− 63.61 to − 50.16)
Australasia	2.32 (1.96 to 2.77)	8393.25 (6908.82 to 10,347.07)	− 30.56 (− 40.59 to − 18.45)	0.54 (0.45 to 0.64)	1.14 (0.96 to 1.31)	− 73.77 (− 77.01 to − 70.04)	101.07 (69.22 to 144.91)	359.99 (239.04 to 527.64)	− 39.35 (− 48.20 to − 29.41)
High-income Asia Pacific	7.25 (6.12 to 8.63)	3744.86 (3032.34 to 4726.20)	− 48.66 (− 54.28 to − 42.81)	4.95 (3.76 to 6.27)	0.83 (0.66 to 1.04)	− 88.21 (− 90.28 to − 83.00)	335.83 (237.18 to 464.86)	160.29 (105.89 to 237.58)	− 60.86 (− 66.93 to − 54.76)
High-income North America	35.61 (31.84 to 39.98)	9848.14 (8624.26 to 11,312.08)	9.59 (1.22 to 19.19)	4.37 (3.59 to 4.70)	0.83 (0.70 to 0.88)	− 49.87 (− 58.80 to − 47.11)	1.48 (1.02 to 2.06)*	412.95 (282.52 to 584.39)	2.82 (− 4.42 to 10.63)
Southern Latin America	4.43 (3.79 to 5.24)	6450.18 (5427.78 to 7800.39)	− 3.67 (− 12.12 to 5.39)	0.88 (0.73 to 1.01)	1.07 (0.89 to 1.21)	− 61.76 (− 68.23 to − 55.41)	190.58 (130.42 to 271.19)	275.77 (188.18 to 399.41)	− 17.38 (− 26.20 to − 8.53)
Western Europe	27.04 (22.87 to 31.92)	5893.41 (4900.26 to 7117.65)	− 29.27 (− 35.13 to − 23.57)	6.90 (5.73 to 7.83)	0.70 (0.61 to 0.79)	− 77.32 (− 79.48 to − 74.71)	1.15 (0.79 to 1.64)*	245.62 (162.85 to 361.91)	− 38.04 (− 44.29 to − 32.32)
Andean Latin America	2.69 (2.01 to 3.59)	4215.37 (3152.42 to 5621.36)	− 22.78 (− 33.98 to − 11.16)	0.52 (0.40 to 0.66)	0.95 (0.73 to 1.19)	− 77.09 (− 83.11 to − 68.78)	121.61 (78.55 to 185.55)	192.28 (124.91 to 291.95)	− 51.49 (− 62.11 to − 39.47)
Caribbean	2.74 (2.30 to 3.29)	6072.63 (5048.46 to 7345.09)	− 14.59 (− 18.39 to − 10.31)	2.23 (1.69 to 2.83)	4.54 (3.43 to 5.83)	− 43.61 (− 54.70 to − 30.58)	193.00 (146.20 to 249.88)	422.79 (315.68 to 553.54)	− 30.55 (− 39.15 to − 21.19)
Central Latin America	7.92 (6.22 to 10.05)	3244.35 (2546.19 to 4160.40)	− 26.17 (− 32.45 to − 19.81)	3.42 (2.81 to 4.05)	1.49 (1.23 to 1.76)	− 77.17 (− 81.63 to − 72.82)	403.92 (283.11 to 574.65)	166.15 (116.28 to 237.45)	− 50.80 (− 58.72 to − 43.09)
Tropical Latin America	9.90 (7.74 to 12.69)	4907.35 (3771.52 to 6383.32)	− 18.25 (− 24.50 to − 12.63)	2.94 (2.61 to 3.45)	1.28 (1.14 to 1.50)	− 62.84 (− 67.42 to − 56.01)	466.00 (321.85 to 685.48)	227.28 (154.50 to 339.89)	− 33.18 (− 40.67 to − 26.47)
North Africa and Middle East	22.13 (18.62 to 26.44)	3819.33 (3262.52 to 4512.65)	− 7.88 (− 12.40 to − 3.15)	32.08 (26.20 to 38.35)	8.39 (6.86 to 9.92)	− 58.97 (− 68.18 to − 49.33)	1.68 (1.32 to 2.12)*	324.16 (259.23 to 397.53)	− 47.56 (− 56.02 to − 39.12)
South Asia	39.87 (33.20 to 47.77)	2443.40 (2029.82 to 2909.76)	− 10.86 (− 16.97 to − 7.75)	232.19 (160.83 to 316.30)	18.95 (12.92 to 26.43)	− 51.19 (− 62.08 to − 39.77)	6.91 (5.21 to 8.70)*	472.00 (350.52 to 601.23)	− 49.56 (− 59.09 to − 39.38)
East Asia	26.50 (21.61 to 32.72)	2025.52 (1577.43 to 2631.41)	− 14.15 (− 18.54 to − 10.25)	27.21 (22.73 to 33.03)	1.59 (1.33 to 1.93)	− 75.93 (− 84.97 to − 64.10)	1.53 (1.14 to 2.06)*	106.42 (75.33 to 152.05)	− 50.71 (− 63.62 to − 38.80)
Oceania	0.53 (0.47 to 0.60)	4265.16 (3834.97 to 4731.89)	− 21.74 (− 24.60 to − 18.77)	2.56 (1.89 to 3.45)	46.76 (34.38 to 63.61)	− 31.17 (− 46.86 to − 13.16)	95.04 (74.14 to 122.20)	1102.21 (863.70 to 1431.32)	− 31.58 (− 44.99 to − 15.66)
Southeast Asia	22.36 (19.26 to 26.37)	3431.82 (2926.72 to 4059.76)	− 8.17 (− 10.99 to − 5.08)	72.06 (61.16 to 81.55)	13.79 (11.64 to 15.64)	− 55.42 (− 65.27 to − 43.68)	2.68 (2.25 to 3.16)*	433.23 (365.45 to 509.73)	− 49.68 (− 56.81 to − 41.31)
Central Sub-Saharan Africa	4.12 (3.36 to 5.10)	3081.67 (2653.81 to 3633.35)	− 15.91 (− 19.94 to − 11.20)	9.79 (6.18 to 17.11)	20.63 (12.04 to 41.64)	− 33.87 (− 50.23 to − 13.03)	479.54 (341.41 to 658.75)	572.95 (389.25 to 907.95)	− 37.73 (− 51.18 to − 21.32)
Eastern Sub-Saharan Africa	17.78 (14.42 to 22.25)	4151.16 (3582.13 to 4898.70)	− 19.14 (− 22.59 to − 15.22)	19.76 (15.36 to 28.24)	11.29 (8.54 to 17.52)	− 45.55 (− 53.99 to − 34.03)	1.45 (1.12 to 1.86)*	450.86 (356.42 to 582.77)	− 42.96 (− 50.79 to − 31.78)
Southern Sub-Saharan Africa	2.66 (1.92 to 3.41)	3476.21 (2532.37 to 4396.58)	− 15.95 (− 22.47 to − 9.98)	7.11 (6.30 to 8.29)	13.78 (12.14 to 16.11)	− 39.34 (− 53.48 to − 27.19)	298.97 (249.02 to 366.22)	446.49 (378.51 to 530.69)	− 40.06 (− 48.86 to − 31.57)
Western Sub-Saharan Africa	14.05 (11.32 to 17.96)	3087.32 (2629.03 to 3669.71)	− 18.11 (− 20.96 to − 14.97)	24.11 (19.91 to 29.11)	13.13 (10.95 to 15.72)	− 43.01 (− 52.00 to − 31.48)	1.34 (1.08 to 1.67)*	425.22 (354.05 to 508.79)	− 39.61 (− 48.11 to − 29.74)

*No, in million (95% UI)

### Regional level

The number of prevalent asthma cases increased from 226.9 million in 1990 to 262.41 million in 2019. South Asia (39.87, 95%UI 33.20 to 47.77), High-income North America (35.61, 95%UI 31.84 to 39.98), and Western Europe (27.04, 95%UI 22.87 to 31.92) had the highest number of prevalent cases in 2019 (unit: million). Point deaths from asthma increased from 460.01 thousand in 1990 to 461.07 thousand in 2019, and point DALYs for asthma decreased from 22.32 million in 1990 to 21.55 million in 2019. South Asia, Southeast Asia, and North Africa and Middle East had the highest number of deaths (232.19, 95% UI 160.83 to 316.30; 72.06, 95%UI 61.16 to 81.55; and 32.08, 95%UI 26.20 to 38.35; unit: thousand) and the highest number of DALYs (6.91, 95%UI 5.21 to 8.70; 2.68, 95%UI 2.25 to 3.16; and 1.68, 95%UI 1.32 to 2.12; unit: million) in 2019 (Table 1).

High-income North America (9848.14) and Australasia (8393.25) had the highest age-standardized prevalence of more than 7,000 per 100,000 people for asthma, whereas East Asia (2025.52), Central Asia (2277.44), and South Asia (2443.40) had the lowest. A total of seven regions had an age-standardized point mortality of more than ten per 100,000 people for asthma, including Oceania (46.76), four Sub-Saharan African regions [Central Sub-Saharan Africa (20.63), Southern Sub-Saharan Africa (13.78), Western Sub-Saharan Africa (13.13), and Eastern Sub-Saharan Africa (11.29)], and two Asian regions [South Asia (18.95) and Southeast Asia (13.79)]. While six regions had point age-standardized mortality of less than one per 100,000 people for asthma, Western Europe (0.70), Eastern Europe (0.70), and Central Europe (0.81) had the lowest values. The leading point age-standardized DALYs rate for asthma (per 100,000) was observed in Oceania (1102.21), which was almost twice that in the next region, Central Sub-Saharan Africa (572.95). East Asia (106.42), Eastern Europe (124.09), and High-income Asia Pacific (160.29) had the lowest age-standardized DALYs rate for asthma (per 100,000) (Table 1). Age-standardized prevalence, mortality, and DALYs rate by sex are shown in Additional file 1: Figure S1 to Figure S3.

From 1990 to 2019, High-income Asia Pacific (− 48.66%), Eastern Europe (− 42.64%), and Australasia (− 30.56%) experienced the greatest decline in age-standardized prevalence of asthma, and only High-income North America (9.59%) experienced an increase. The decreases in age-standardized deaths from asthma from 1990 to 2019 was observed in all regions, which recorded a decrease from − 31.17 to − 88.21%. High-income Asia Pacific (− 88.21%), Eastern Europe (− 83.43%), and Central Europe (− 82.49%) were the only regions that experienced a decrease greater than 80% during this period. High-income Asia Pacific (− 60.86%), Eastern Europe (− 56.99%), and Andean Latin America (− 51.49%) regions experienced the greatest decline in age-standardized DALYs rates for asthma between 1990 and 2019, while only High-income North America (2.82%) experienced an increase (Table 1). The percent change in age-standardized prevalence, mortality, and DALYs rates by sex is shown in Additional file 1: Figure S4 to Figure S6.

### National level

**Fig. 1:**
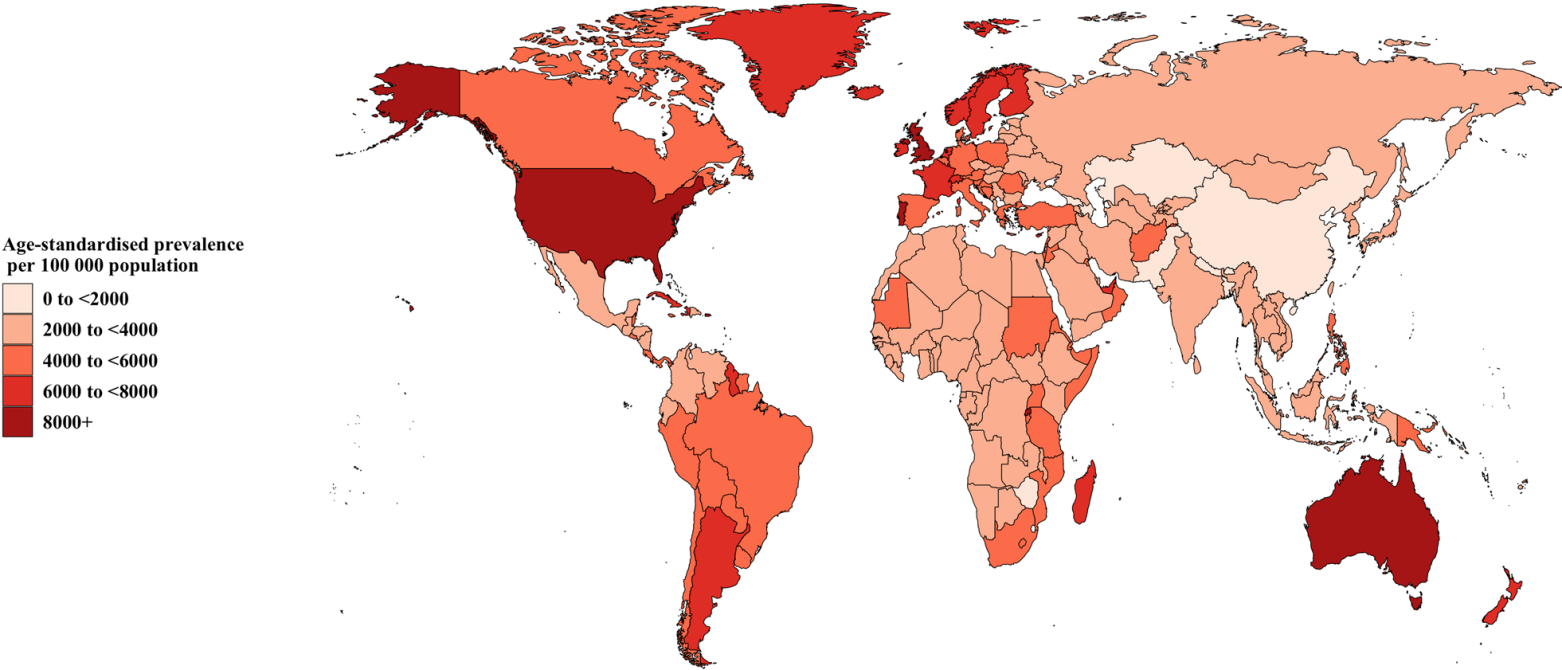
Age-standardized prevalence of asthma per 100,000 population in 2019

**Fig. 2:**
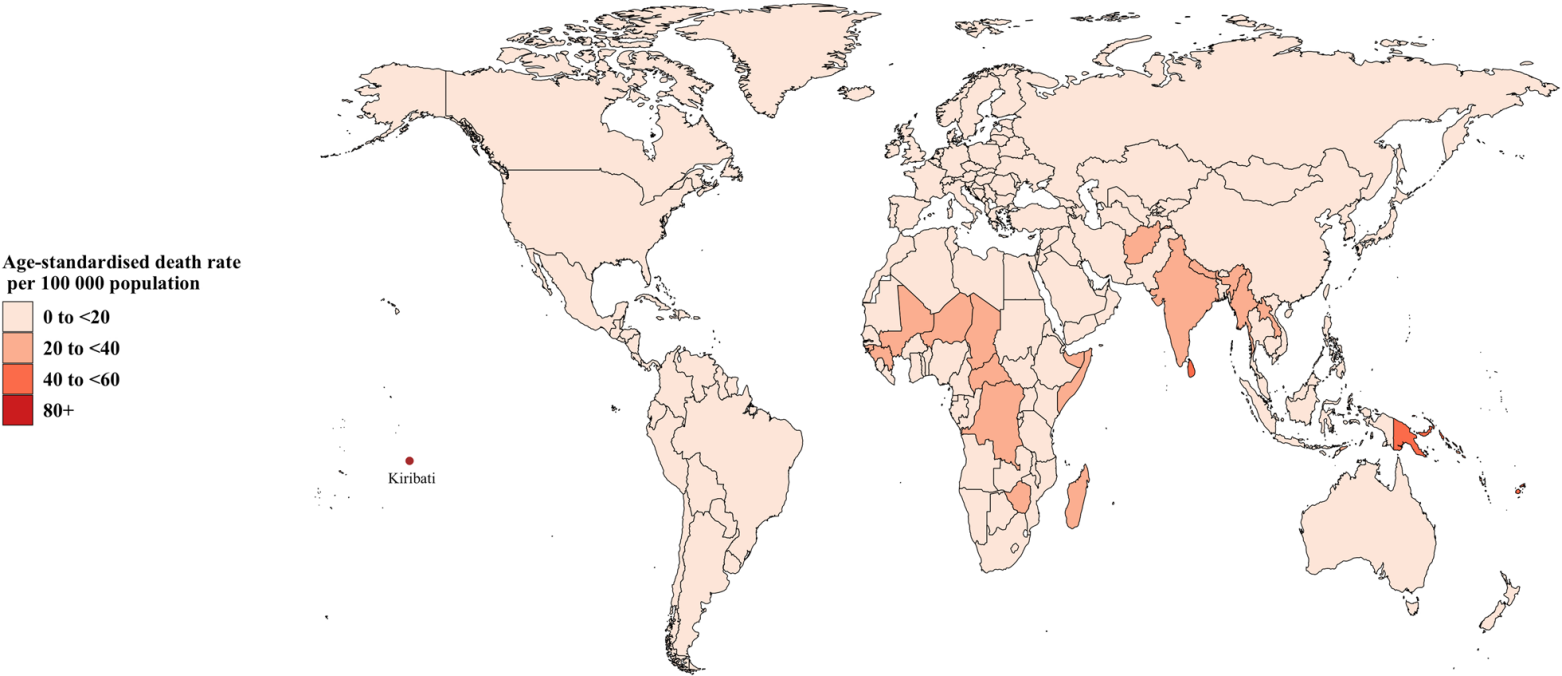
Age-standardized death rate of asthma per 100,000 population in 2019

The age-standardized prevalence of asthma for 204 countries and territories ranged from 1072.46 to 10,399.27 per 100,000 in 2019. The United States of America (10,399.27, 95%UI 9140.29 to 11,903.19), the United Kingdom (9166.57, 95%UI 7645.04 to 11,034.51), and Portugal (9106.18, 95%UI 7499.66 to 11,069.60) had the highest age-standardized prevalence of asthma per 100,000, while Nepal (1072.46, 95%UI 932.39 to 1,214.78), Lesotho (1377.07, 95%UI 1185.73 to 1567.73), and Bangladesh (1390.91, 95%UI 1217.24 to 1574.20) had the lowest (Fig. 1 and Additional file 1: Table S2). Country specific age-standardized mortality for asthma ranged from 0.26 to 80.50 per 100,000 people in 2019. Kiribati (80.50, 95%UI 59.22 to 104.19), Papua New Guinea (55.81, 95%UI 39.05 to 80.31), and Fiji (43.33, 95%UI 34.22 to 54.76) had the highest estimates per 100,000 people. A total of 49 countries had an age-standardized asthma mortality of less than one per 100,000 people in 2019, and Greece (0.26, 95%UI 0.21 to 0.31) had the lowest (Fig. 2 and Additional file 1: Table S3). The national age-standardized DALYs rate of asthma for more than 1000 per 100,000 people was observed in Kiribati (1795.09, 95%UI 1411.44 to 2242.16) and Papua New Guinea (1250.25, 95%UI 941.24 to 1697.62) in 2019. And Armenia (91.09, 95%UI 59.15 to 136.04) was the only country with asthma DALYs rate below 100 per 100,000 in 2019. (Additional file 1: Figure S7 & Table S4).

New Zealand (− 55.14%, 95%UI − 59.09 to − 50.36) and Japan (− 52.82%, 95%UI − 57.7 to − 47.85) experienced a decrease in age-standardized prevalence of more than 50% since 1990. While 29 countries showed an increase in age-standardized prevalence since 1990, Oman (31.45%, 95%UI 19.2 to 49.62), followed by Saudi Arabia (26.24%, 95%UI 16.54 to 40.04) and Viet Nam (18.80%, 95%UI 13.14 to 25.63) showed the largest increase (Additional file 1: Figure S8 & Table S2). Age-standardized mortality decreased in all countries and territories since 1990, with the Republic of Korea (− 90.23%, 95%UI − 92.41 to − 83.76) showing the largest decrease (Additional file 1: Figure S9 & Table S3). From 1990 to 2019, the Maldives (− 75.61%, 95%UI − 83.03 to − 64.26), Guatemala (− 72.90%, 95%UI − 79.21 to − 63.57), and the Republic of Korea (− 72.35%, 95%UI − 79.18 to − 60.84) experienced the greatest decrease in DALYs for asthma, while an increase was seen only in Montenegro (6.68%, 95%UI − 1.26–15.94), Oman (6.03%, 95%UI − 10.86–24.02), United States of America (4.44%, 95%UI − 3.45–12.86), and Paraguay (3.13%, 95%UI − 7.85–13.73) (Additional file 1: Figure S10 & Table S4).

### Age and sex pattern

**Fig. 3:**
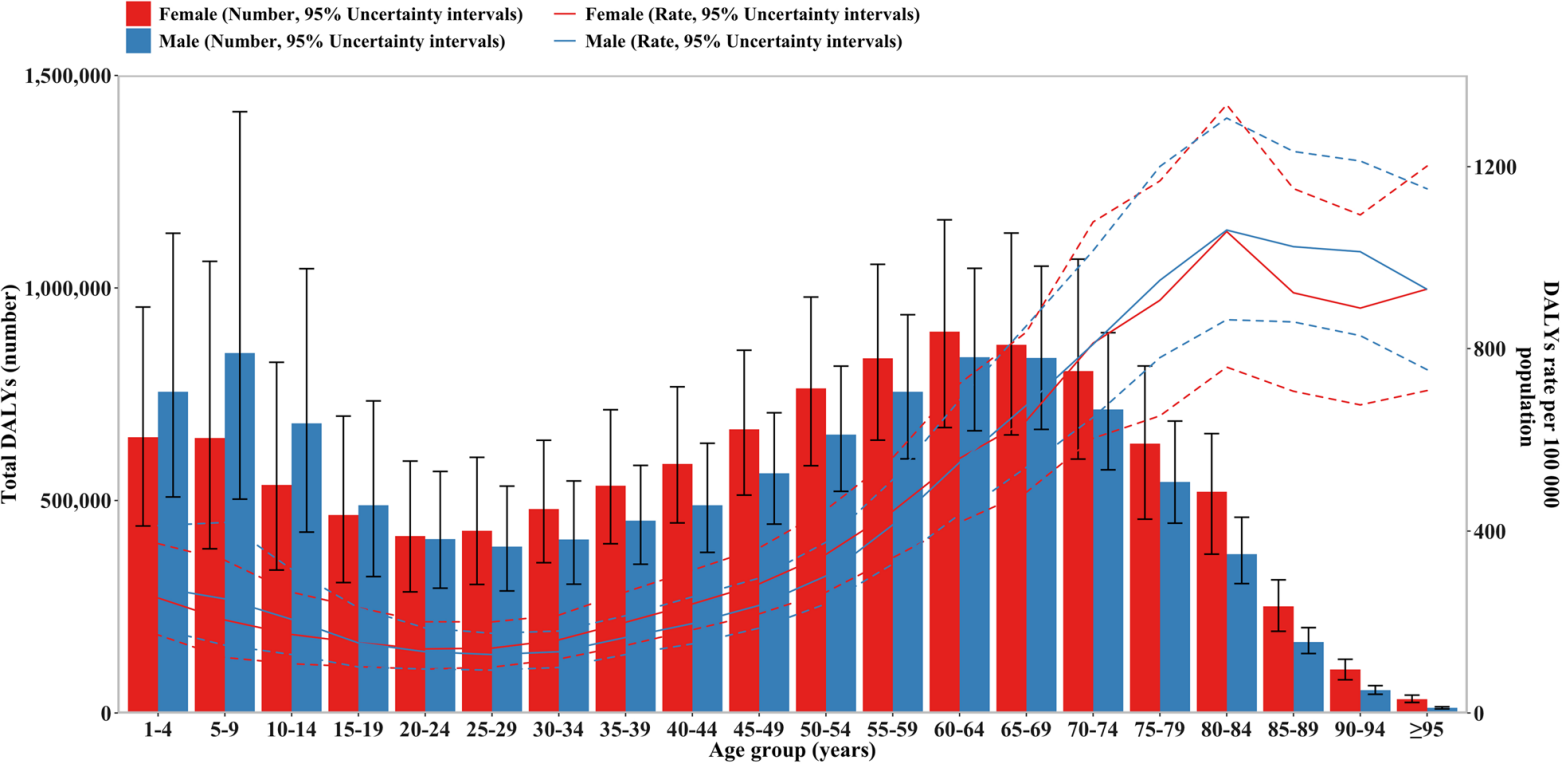
Number of DALY cases globally and DALY rate of asthma per 100,000 population, by age and sex in 2019. Boxes indicate DALY cases with 95% uncertainty intervals for men and women. DALY: disability adjusted life year

More asthma point prevalence cases were observed in children aged five to nine years, with about 19 million in males and 14 million in females, and with a relatively high age-standardized point prevalence of 5717.50 per 100,000 people in males and 4508.27 per 100,000 people in females (Additional file 1: Figure S11). Males aged 75–79 years and females aged 80–84 years had the highest number of asthma death cases, 28,297.80 cases and 36,492.10 cases, respectively. The highest age-standardized point mortality was observed in people older than 95 years, with 126.14 per 100,000 in males and 118.29 per 100,000 in females (Additional file 1: Figure S12). The number of asthma DALYs for males and females showed a bimodal distribution. The highest number of asthma DALYs in males was in children aged 5–9 years, which totaled 847,222.3 DALYs, whereas in females it was in adults aged 60–64 years, with a total of 896,868.8 DALYs. The number of asthma DALYs was higher in female adults older than 20–24 years. The peak age-standardized DALYs rate was seen in people aged 80 to 84 years [males: 1060.8, females: 1057.7, unit per 100,000 people] (Fig. 3).

### Association with the sociodemographic index

**Fig. 4:**
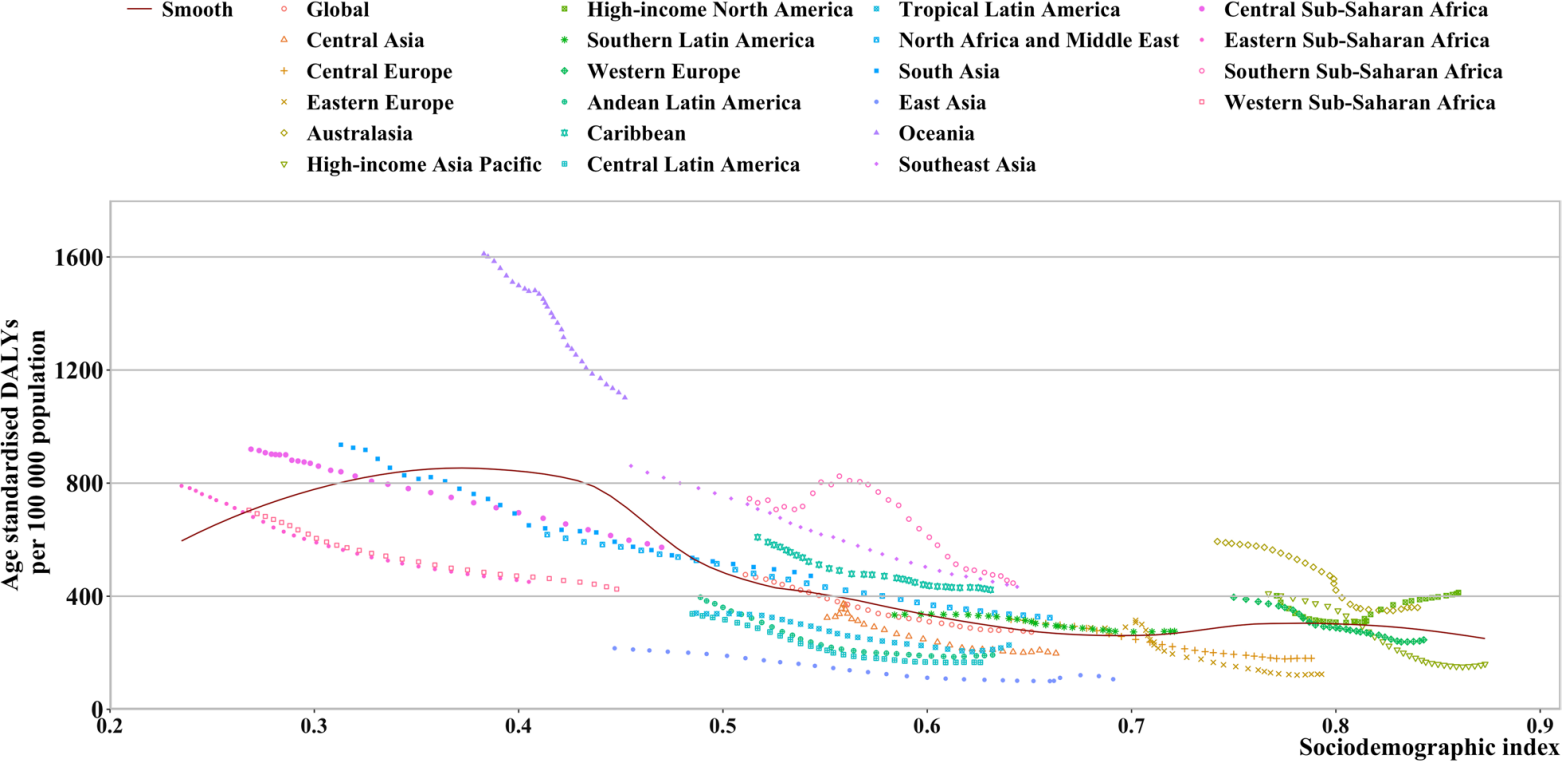
Age-standardized disability adjusted life year (DALY) rates of asthma for the 21 Global Burden of Disease regions by sociodemographic index, 1990–2019. Thirty points are plotted for each region and show the observed age-standardized DALY rates from 1990 to 2019 for that region. Expected values, based on the sociodemographic index and disease rates in all locations, are shown as a solid line. Regions above the solid line represent a higher than expected burden and regions below the line show a lower than expected burden

At the global level, asthma DALYs decreased with the increase in SDI (Fig. 4). At the regional level, a non-linear relationship was observed between age-standardized asthma DALYs rate and SDI, and the lowest age-standardized DALYs rate was observed at an SDI of approximately 0.70. Oceania, Southern Sub-Saharan Africa, Southeast Asia, the Caribbean, and Australasia had higher DALYs rates from 1990 to 2019 than expected based on their SDI. At the national level, a L-shaped relationship was observed between asthma DALYs rate and SDI (Additional file 1: Figure S13). Many countries had higher than expected DALYs rates in 2019 based on their SDI, which was seen in Kiribati, Papua New Guinea, Fiji, and the Central African Republic.

### Age-period-cohort analysis

Declines were observed in asthma prevalence and mortality (asthma prevalence net drift = − 1.45, unit: %/year; p < 0.05; asthma mortality net drift = − 2.62, unit: %/year; p < 0.05). Comparison between local drifts and net drifts showed statistical significance (p < 0.05), suggesting that the overall annual percentage change (net drift) in asthma prevalence and mortality could not adequately explain the variations in age-specific annual percentage change (local drifts) over time. The longitudinal age curve showed that asthma prevalence had an approximately W-shaped pattern, peaking at age 7.5 years. The results showed that asthma mortality probably had a J-shaped pattern, with a higher rate in children younger than 7.5 years (rate = 7.68 in children aged 2.5 years), and it was lowest at age 12.5 years (rate = 1.13) and then increased with age.

Period RR results showed higher relative risks for asthma prevalence and mortality before 2002 (asthma prevalence: RR = 1.26 in 1992, RR = 1.13 in 1997; asthma mortality: RR = 1.29 in 1992, RR = 1.16 in 1997). Cohort RR results showed higher relative risks in asthma prevalence and mortality before the 1955 birth cohort (both asthma prevalence and mortality: RR = 1.00 in 1955) and lower relative risks in subsequent birth cohorts (Additional file 1: Figure S14).

### Risk factors

**Fig. 5:**
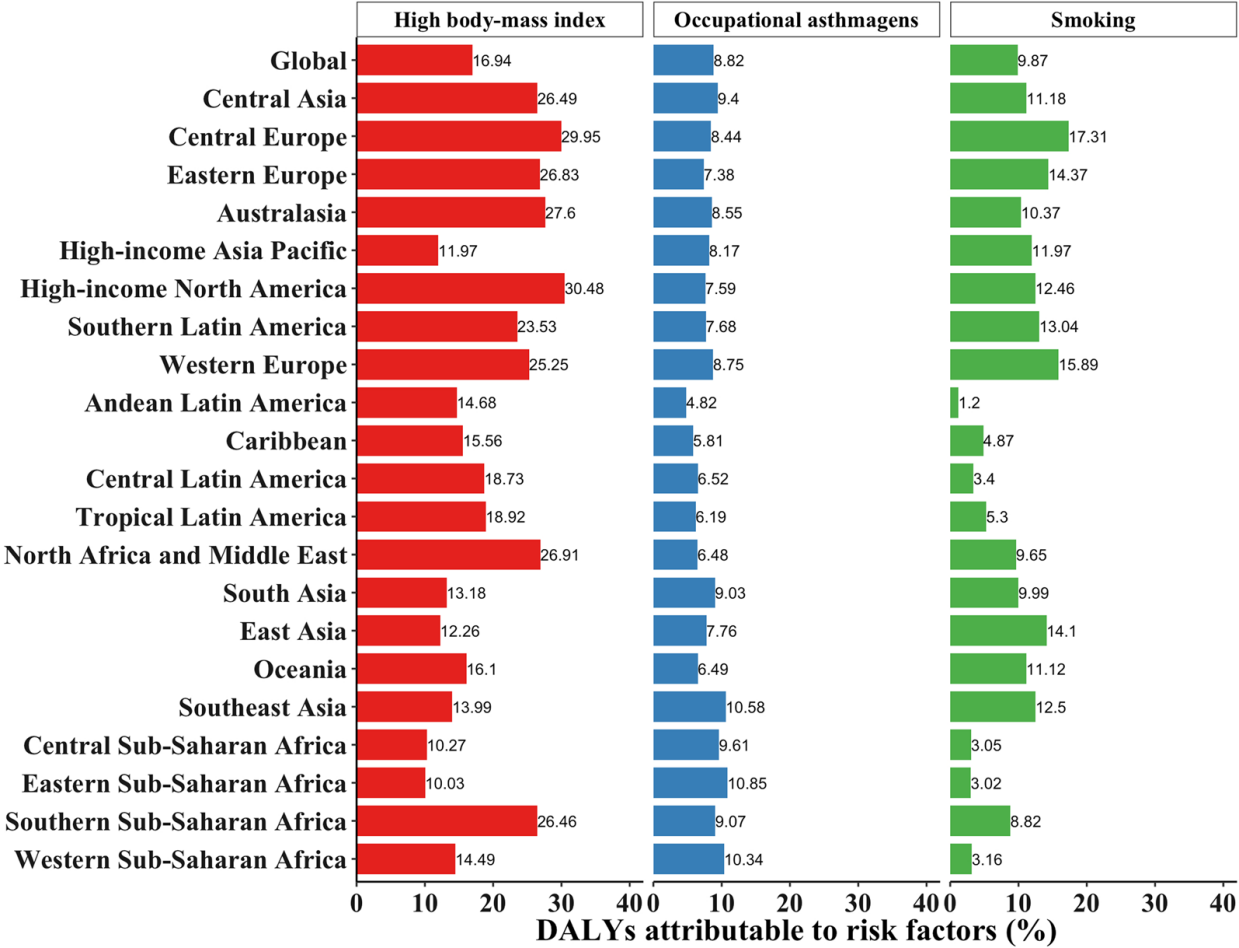
Percentage of disability adjusted life years (DALYs) due to asthma attributable to each risk factor for the 21 Global Burden of Disease regions in 2019

Factors such as high body mass index, occupational asthmagens, and smoking had some influence on asthma DALYs at the global level, accounting for 16.94%, 8.82%, and 9.87%, respectively. At the regional level, 10.03 to 30.48% of asthma DALYs were due to high body mass index, 4.82–10.85% of asthma DALYs were due to occupational asthmagens, and 1.2–17.31% of asthma DALYs were due to smoking (Fig. 5). The leading risk factor in terms of risk-attributable DALYs globally in 2019 was high body-mass index in females (risk-attributable DALYs = 19.06%) (Additional file 1: Figure S15) and smoking in males (risk-attributable DALYs = 15.38%) (Additional file 1: Figure S16).

At the global level, asthma DALYs attributed to high body mass index showed a W-shaped distribution, in which it increased rapidly in adults aged 20–24 years (15.05%) and peaked at age of 45–49 years (23.64%), decreased to a low level at age of 80–84 years (14.47%), and then increased again. Asthma DALYs attributed to occupational asthmagens showed an inverted U-shaped distribution in individuals aged 15–84 years and peaked at age of 35–39 years (16.25%). Asthma DALYs attributed to smoking showed an inverted U-shaped distribution in people aged 30–95 years and peaked at age of 60–64 years (16.31%) (Additional file 1: Figure S17). Notably, in every age group between 25 and 74 years, more than 20% of asthma DALYs in females were attributed to high body mass index (Additional file 1: Figure S18). And asthma DALYs attributed to smoking in males showed a U-shaped distribution in individuals older than 35 years, with a peak of 25.87% at age of 60–64 years (Additional file 1: Figure S19).

## Discussion

In this study, the burden of asthma was analyzed based on the GBD database. We found that age-standardized asthma prevalence, mortality, and DALYs had marked declines in the past 30 years, but the number of prevalent and death cases of asthma increased. Many countries had higher DALYs rates in 2019 than expected based on their SDI. The Age-Period-Cohort effects of asthma prevalence and mortality were determined. In terms of the percentage of asthma-related DALYs attributable to each risk factor, high body mass-index had the largest effect, while occupational asthmagens and smoking had a similar effect at the global level.

From 1990 to 2019, age-standardized asthma prevalence and DALYs decreased globally, but increased in some countries such as Oman, Saudi Arabia and Viet Nam, and so on. Previous epidemiological studies of asthma in Oman, Saudi Arabia, and Viet Nam mentioned some reasons for the high disease burden of asthma, such as inadequate use of preventive asthma medications and inadequate use of pulmonary function tests to diagnose or monitor asthma progression [21, 22]. For some countries, it could also be important to improve awareness of asthma in the population [23]. A study in Portugal also concluded that physicians needed to improve their knowledge of asthma diagnosis and treatment [24].

On the other hand, our results showed that countries could have a high age-standardized asthma prevalence but a low age-standardized asthma death rate, which was mainly observed in high-income countries. The main reason for this phenomenon might be that most high-income countries did a good job in managing medicines and standard care for asthma patients. Thus, although some unavoidable risk factors such as urbanization led to high asthma prevalence, the asthma mortality was maintained at a low level. In contrast, some low- and middle-income countries (LMICs) had low age-standardized asthma prevalence but high age-standardized asthma death rates. Effective, accessible, and affordable medicines were a major challenge for LMICs [25, 26]. For example, in some countries, long-term standards for medical care were not established and there were few outstanding physicians and organizations, so medical care for asthma patients was generally poor. Therefore, there are still many unnecessary deaths from asthma in these LMICs. The location of these LMICs, such as Kiribati, Papua New Guinea, and Fiji, is near the ocean, and the ocean climate may influence the prevalence of asthma. However, the relationship between ocean climate and the prevalence of asthma was not well-determined [27].

Stratified by sex and age, we found that the distribution of asthma DALYs had some changes over the past 10 years. Results from IHME’s analysis in 2010 showed that peak asthma DALYs occurred at age of 10–14 years and at age of 75–79 years, and the trough value was at age of 30–34 years [16]. However, in our study, the global asthma DALYs in 2019 found that both the peak and trough DALYs occurred at an earlier age. Additionally, the results of the IHME study showed that the distribution of asthma DALYs was similar in males and females before the age of 30–34 years, and the burden of asthma was higher in males than that in females with age. However, our results showed that asthma DALYs in females was higher than that in males in people older than 20 years, which was consistent with the Centers for Disease Control and Prevention (US) reports indicating that women had a higher prevalence than men in adults, based on the current surveillance summaries [28]. But trends of asthma burden would not be the same in every country or region. A survey derived from seven cities in China showed that men had higher asthma prevalence among people older than 15 years in six cities [29]. The distribution patterns of asthma prevalence and DALYs by age and sex, which were influenced by various risk factors, were not well explained. For instance, the phenomenon that females showed higher asthma prevalence after puberty might be related to the pathogenic effect of sex hormones [30].

In terms of Age-Period-Cohort analysis, longitudinal age curves showed that asthma was most prevalent in children, while the elderly had the highest asthma mortality. Childhood asthma is influenced by a multitude of factors that contribute to its increased prevalence. These factors include viral infections, air pollution, genetic susceptibility, obesity, and abnormal immune maturation during early life [31–34]. While the factors causing asthma in the elderly could be mainly attributed to social factors or in conjunction with comorbidities. However, the relationship between these factors and the pathogenesis of asthma is complex, and further studies on the biological mechanism are needed [35, 36]. The period effect on prevalence and mortality of asthma decreased from 1990 to 2019, which could be related to the improvement in the management of asthma [2, 37, 39]. When inhaled corticosteroids (ICS) or combination therapy of ICS with long-acting beta2 agonists (LABA) or long-acting muscarinic antagonists (LAMA) were introduced for asthma, great improvements in symptom control were observed, and exacerbations and asthma-related mortality also decreased [2]. Methylxanthines such as theophylline and aminophylline were also used for many years to treat patients with asthma [40]. Among biologic agents, omalizumab helps reduce the frequency and severity of asthma attacks by binding to immunoglobulin E (IgE), while mepolizumab and reslizumab are monoclonal antibodies that target interleukin-5 (IL-5) and are used to treat severe eosinophilic asthma [2]. However, based on GBD 2019, the drug-related reduction in asthma mortality could not be estimated due to lack of data on the different drugs, and further studies are needed to assess the related burden. Additionally, previous studies found that changes in policy and social economics could influence air quality, tobacco use, and awareness of early screening or diagnosis of asthma, which could also affect disease rates [41, 42].

Regarding risk factors, our results showed that high body mass index was most critical for DALYs due to asthma at the global level, which was consistent with the findings of previous studies [6, 43–45]. Li et al. found that the age-standardized mortality of asthma attributed to smoking decreased shapely from 1990 to 2017, while in 2017, the age-standardized mortality of asthma attributed to high body mass index had a larger effect than that of smoking [6]. However, smoking was still thought to be one of the main risk factors for asthma [46, 47]. The results of the International Study of Asthma and Allergies in Childhood (ISAAC) showed that open-fire cooking and maternal tobacco use could be the risk factors for asthma symptoms in children aged 6–7 years and 13–14 years [48]. When analyzing the percentage of DALYs due to asthma attributable to smoking, our results also indicated that smoking had a large impact on asthma DALYs, which was 9.87% at the global level. In Central Europe, Western Europe, and Eastern Europe, smoking had a greater impact on asthma DALYs, accounting for 17.31%, 15.89%, and 14.37%, respectively. As a result, we considered it essential for governments to continue the decisions on tobacco control and dietary adjustments, and to improve measures to prevent occupational asthmagens, because occupational asthmagens also had a large impact on DALYs [49]. And more high-quality epidemiological studies are needed to identify the potential risk factors for asthma, to identify the cause, and to reduce exposure to these factors.

Our study updated asthma burden data and analyzed changes over the past three decades, which could provide clues for future studies and useful information for policy makers. However, our study had some limitations. First, our study might provide little interesting information about the burden of asthma in a clinical context because of a lack of data on emergency department visits, hospital admissions, and use of key asthma medications. Second, our study considered only the best-known risk factors for asthma and not some potential factors such as household air pollution from solid fuels and particulate matter in the workplace. Due to the lack of data on the risk burden of obesity, future epidemiologic studies are needed to determine the impact of other potential factors. Third, many countries did not have registration systems to record deaths, so estimates had to be obtained from autopsy studies. However, in these studies, estimates of asthma-related deaths did not distinguish between the different types of chronic respiratory diseases that should not be recorded, resulting in underestimation. Fourth, although multiple calculation methods, correction of disease miscoding, and redistribution of waste codes were used in the GBD study, the potential inaccuracy of the data also had to be considered.

## Conclusions

The age-standardized disease burden rate of asthma declined by some extent over the past 30 years. However, the number of prevalent cases and deaths of asthma continues to increase and the burden remains severe, especially in countries with a low SDI. Age-Period-Cohort effects of asthma prevalence and mortality were determined. High body mass index becomes the most important risk factor for DALYs due to asthma at the global level.

## Supplementary Information

**Additional file 1: Table S1.** Definition and data sources. **Table S2.** Prevalence of asthma in 1990 and 2019 and the percentage change in the age-standardized ratesper 100,000, by location. **Table S3.** Deaths of asthma in 1990 and 2019 and the percentage change in the age-standardized ratesper 100,000, by location. **Table S4.** Disability adjusted life yearsof asthma in 1990 and 2019 and the percentage change in the age-standardized ratesper 100,000, by location. **Figure S1**. The age-standardized prevalence of asthma in 2019 for the 21 Global Burden of Disease regions, by sex. **Figure S2**. The age-standardized deaths rate of asthma in 2019 for the 21 Global Burden of Disease regions, by sex. **Figure S3**. The age-standardized DALY rate of asthma in 2019 for the 21 Global Burden of Disease regions, by sex. DALY: disability adjusted life year. **Figure S4**. The percentage change in the age-standardized prevalence of asthma from 1990 to 2019 for the 21 Global Burden of Disease regions, by sex. **Figure S5**. The percentage change in the age-standardized death rate of asthma from 1990 to 2019 for the 21 Global Burden of Disease regions, by sex. **Figure S6**. The percentage change in the age-standardized DALY rate of asthma from 1990 to 2019 for the 21 Global Burden of Disease regions, by sex. DALY: disability adjusted life year. **Figure S7**. Age-standardized disability adjusted life yearsrate of asthma per 100 000 population in 2019. **Figure S8**. The percentage change in the age-standardized prevalence of asthma from 1990 to 2019 for the 204 Global Burden of Disease countries and territories. **Figure S9**. The percentage change in the age-standardized death rate of asthma from 1990 to 2019 for the 204 Global Burden of Disease countries and territories. **Figure S10**. The percentage change in the age-standardized DALY rate of asthma from 1990 to 2019 for the 204 Global Burden of Disease countries and territories. **Figure S11**. Number of prevalent cases globally and prevalence of asthma per 100 000 population, by age and sex in 2019. Boxes indicate prevalent cases with 95% uncertainty intervals for men and women. **Figure S12**. Number of death cases globally and death rate of asthma per 100 000 population, by age and sex in 2019. Boxes indicate death cases with 95% uncertainty intervals for men and women. **Figure S13.** Age-standardized disability adjusted life yearrates of asthma for the 204 Global Burden of Disease countries and territories by sociodemographic index, in 2019. Points are plotted for each country and territory and show the observed age-standardized DALY rates in 2019 for that country or territory. Expected values, based on the sociodemographic index and disease rates in all locations, are shown as a solid line. Countries and territories above the solid line represent a higher than expected burden and countries and territories below the line show a lower than expected burden. **Figure S14.** The Age-Period-Cohort analysis in prevalence and death rate of asthma; A: The age effect in the prevalence of asthma; B: The age effect in the death rate of asthma; C: The period effect in the prevalence of asthma; D: The period effect in the death rate of asthma; E: The cohort effect in the prevalence of asthma; F: The cohort effect in the death rate of asthma. **Figure S15.** Percentage of DALYs due to asthma attributable to risk factors among females for 21 GBD regions in 2019. DALY = disability adjusted life years. **Figure S16.** Percentage of DALYs due to asthma attributable to risk factors among males for 21 GBD regions in 2019. DALY = disability adjusted life years. **Figure S17.** Percentage of DALYs due to asthma attributable to each risk factor, by age, in 2019. DALY = disability adjusted life years. **Figure S18.** Percentage of DALYs due to asthma attributable to each risk factor among females, by age, in 2019. DALY = disability adjusted life years. **Figure S19.** Percentage of DALYs due to asthma attributable to each risk factor among males, by age, in 2019. DALY = disability adjusted life years.

## Abbreviations

BRICS: Brazil, Russia, India, China, and South Africa
COD: Cause of Death
CODEm: Cause of Death Ensemble modelling
DALYs: Disability adjusted life years
GAN I: Global Asthma Network Phase one
GBD: Global Burden of Disease
GHDx: Global Health Data Exchange
GINA: Global Initiative for Asthma
ICD: International Classification of Disease and Injuries
ICS: Inhaled corticosteroids
IgE: Immunoglobulin E
IL-5: Interleukin-5
IHME: Institute for Health Metrics and Evaluation
ISAAC: International Study of Asthma and Allergies in Childhood
LABA: Long-acting beta2 agonists
LAMA: Long-acting muscarinic antagonists
LMICs: Low- and middle-income countries
RR: Relative risk
SDI: Sociodemographic index
95% UI: 95% Uncertainty interval

## Declarations

### Availability of data and materials

The datasets used and/or analyzed during the current study are available from the corresponding author on reasonable request.
