# Supplementary material for: Global, regional, and national burden of asthma and its attributable risk factors from 1990 to 2019: a systematic analysis for the Global Burden of Disease Study 2019
Source: Respir Res. 2023 Jun 23;24:169. doi: 10.1186/s12931-023-02475-6 (PMC10288698; doi:10.1186/s12931-023-02475-6)
Supplement: Supplementary file 1 — Additional file 1: Table S1. Definition and data sources. Table S2. Prevalence of asthma in 1990 and 2019 and the percentage change in the age-standardized ratesper 100,000, by location. Table S3. Deaths of asthma in 1990 and 2019 and the percentage change in the age-standardized ratesper 100,000, by location. Table S4. Disability adjusted life yearsof asthma in 1990 and 2019 and the percentage change in the age-standardized ratesper 100,000, by location. Figure S1. The age-standardized prevalence of asthma in 2019 for the 21 Global Burden of Disease regions, by sex. Figure S2. The age-standardized deaths rate of asthma in 2019 for the 21 Global Burden of Disease regions, by sex. Figure S3. The age-standardized DALY rate of asthma in 2019 for the 21 Global Burden of Disease regions, by sex. DALY: disability adjusted life year. Figure S4. The percentage change in the age-standardized prevalence of asthma from 1990 to 2019 for the 21 Global Burden of Disease regions, by sex. Figure S5. The percentage change in the age-standardized death rate of asthma from 1990 to 2019 for the 21 Global Burden of Disease regions, by sex. Figure S6. The percentage change in the age-standardized DALY rate of asthma from 1990 to 2019 for the 21 Global Burden of Disease regions, by sex. DALY: disability adjusted life year. Figure S7. Age-standardized disability adjusted life yearsrate of asthma per 100 000 population in 2019. Figure S8. The percentage change in the age-standardized prevalence of asthma from 1990 to 2019 for the 204 Global Burden of Disease countries and territories. Figure S9. The percentage change in the age-standardized death rate of asthma from 1990 to 2019 for the 204 Global Burden of Disease countries and territories. Figure S10. The percentage change in the age-standardized DALY rate of asthma from 1990 to 2019 for the 204 Global Burden of Disease countries and territories. Figure S11. Number of prevalent cases globally and prevalence of asthma per 100 000 populatio [file 12931_2023_2475_MOESM1_ESM.docx]

**Additional Materials:**

1. **Table S1.** Definition and data sources
2. **Table S2.** Prevalence of asthma in 1990 and 2019 and the percentage change in the age-standardized rates (ASRs) per 100,000, by location
3. **Table S3.** Deaths of asthma in 1990 and 2019 and the percentage change in the age-standardized rates (ASRs) per 100,000, by location
4. **Table S4.** Disability adjusted life years (DALYs) of asthma in 1990 and 2019 and the percentage change in the age-standardized rates (ASRs) per 100,000, by location
5. **Figure S1**. The age-standardized prevalence of asthma in 2019 for the 21 Global Burden of Disease regions, by sex
6. **Figure S2**. The age-standardized deaths rate of asthma in 2019 for the 21 Global Burden of Disease regions, by sex
7. **Figure S3**. The age-standardized DALY rate of asthma in 2019 for the 21 Global Burden of Disease regions, by sex. DALY: disability adjusted life year
8. **Figure S4**. The percentage change in the age-standardized prevalence of asthma from 1990 to 2019 for the 21 Global Burden of Disease regions, by sex
9. **Figure S5**. The percentage change in the age-standardized death rate of asthma from 1990 to 2019 for the 21 Global Burden of Disease regions, by sex
10. **Figure S6**. The percentage change in the age-standardized DALY rate of asthma from 1990 to 2019 for the 21 Global Burden of Disease regions, by sex. DALY: disability adjusted life year
11. **Figure S7**. Age-standardized disability adjusted life years (DALYs) rate of asthma per 100 000 population in 2019
12. **Figure S8**. The percentage change in the age-standardized prevalence of asthma from 1990 to 2019 for the 204 Global Burden of Disease countries and territories
13. **Figure S9**. The percentage change in the age-standardized death rate of asthma from 1990 to 2019 for the 204 Global Burden of Disease countries and territories
14. **Figure S10**. The percentage change in the age-standardized DALY rate of asthma from 1990 to 2019 for the 204 Global Burden of Disease countries and territories
15. **Figure S11**. Number of prevalent cases globally and prevalence of asthma per 100 000 population, by age and sex in 2019. Boxes indicate prevalent cases with 95% uncertainty intervals for men and women
16. **Figure S12**. Number of death cases globally and death rate of asthma per 100 000 population, by age and sex in 2019. Boxes indicate death cases with 95% uncertainty intervals for men and women
17. **Figure S13.** Age-standardized disability adjusted life year (DALY) rates of asthma for the 204 Global Burden of Disease countries and territories by sociodemographic index, in 2019. Points are plotted for each country and territory and show the observed age-standardized DALY rates in 2019 for that country or territory. Expected values, based on the sociodemographic index and disease rates in all locations, are shown as a solid line. Countries and territories above the solid line represent a higher than expected burden and countries and territories below the line show a lower than expected burden.
18. **Figure S14.** The Age-Period-Cohort analysis in prevalence and death rate of asthma; A: The age effect in the prevalence of asthma; B: The age effect in the death rate of asthma; C: The period effect in the prevalence of asthma; D: The period effect in the death rate of asthma; E: The cohort effect in the prevalence of asthma; F: The cohort effect in the death rate of asthma
19. **Figure S15.** Percentage of DALYs due to asthma attributable to risk factors among females for 21 GBD regions in 2019. DALY=disability adjusted life years
20. **Figure S16.** Percentage of DALYs due to asthma attributable to risk factors among males for 21 GBD regions in 2019. DALY=disability adjusted life years
21. **Figure S17.** Percentage of DALYs due to asthma attributable to each risk factor, by age, in 2019. DALY=disability adjusted life years
22. **Figure S18.** Percentage of DALYs due to asthma attributable to each risk factor among females, by age, in 2019. DALY=disability adjusted life years
23. **Figure S19.** Percentage of DALYs due to asthma attributable to each risk factor among males, by age, in 2019. DALY=disability adjusted life years

**Table S1.** Definition and data sources

| **Case definition and data sources for asthma** | Asthma is a chronic lung disease marked by spasms in the bronchi usually resulting from an allergic reaction or hypersensitivity and causing difficulty in breathing. In GBD 2019, it is defined asthma as a doctor’s diagnosis and wheezing in the past year. The relevant ICD-10 codes are J45 and J46 (ICD-9 code is 493). Alternative case definitions include self-reported asthma in the past year, self-reported asthma ever, only a doctor’s diagnosis in the past year and only wheezing in the past year. The last full systemic review of the literature on Asthma was done for GBD 2016. The search string was (Asthma[Title/Abstract] AND prevalence[Title/Abstract] AND "Cross-Sectional Studies"[MeSH Terms]) and filtered by studies of humans published between January 2012 and November 2016. Data in literature matching the case definitions above were extracted. In addition to claims data used in GBD 2017, USA claims data for the years 2015 and 2016 were added. The new data for Wave 7 of the English Longitudinal Study of Ageing (ELSA) were also added. Surveys carried out as part of the International Study of Asthma and Allergies in Childhood (ISAAC) collaboration are the most important source of prevalence data in children. Vital registration and surveillance data from the cause of death (COD) database were used to estimate asthma mortality. Verbal autopsy data were not included and were instead mapped to an overall chronic respiratory model. The outlier criteria excluded data points that (1) were implausibly high or low relative to global or regional patterns, (2) substantially conflicted with established age or temporal patterns, or (3) significantly conflicted with other data sources conducted from the same locations or locations with similar characteristics (ie, Socio-demographic Index). A total of 136 countries with 413 total sources provided data for asthma in all measures. |
| --- | --- |
| **Bias adjustments** | In GBD 2019, the bias adjustment methods utilized a MR-BRT model outside of DisMod to allow a more direct comparison between different case definitions and/or study designs. Data that don’t completely match the case definition, doctor’s diagnosis and wheezing in the past year was made a series of adjustments. The estimation of Asthma in a population varies slightly by the case definition used (wheezing and diagnosis, only wheezing, etc). Similarly, claims data is subject to biases. An analysis for GBD 2017 showed that claims data were systemically lower than asthma survey data, probably reflecting selection bias with regard to socioeconomic status. Adjustments are made to these data to correct these biases.  The adjustment is a logit-transformation method in MR-BRT. The general process is described below:  1. Identify data points with overlapping year, age, sex, and location between reference and alternative definitions.  2. Logit transform overlapping data points of alternative and reference case definitions  3. Convert overlapping data points into a difference in logit space using the following equation:  *logit (alternative) − logit (reference)*  4. Use the delta method to compute standard errors of overlapping data points in logit space, then calculate standard error of logit difference using the following equation:  $\sqrt{\left( variance of alternative \right)+(variance of reference)}$  5. Using MR-BRT, conduct a random effects meta-regression to obtain the pooled logit difference of alternative to reference  6. Apply the pooled logit difference to all data points of alternative case definitions using the following equation:  *new_estimate_ = inverse.logit((logit(alternative)) – (pooled logit difference))*  7. Calculate new standard errors using the delta method, accounting for gamma (between-study heterogeneity)  Data derived from claims from commercial health insurance in the United States were also adjusted using a factor estimated in MR-BRT. To account for this, a MarketScan 2000 coefficient and a separate MarketScan coefficient for the remaining years of MarketScan data were estimated, by comparing the national values in these datasets to national asthma estimates from the USA National Health and Nutrition Examination Survey and National Health Interview Surveys. |
| **Estimate deaths due to asthma** | The standard CODEm modelling approach was applied to estimate deaths due to asthma. Separate models were conducted for male and female mortality, and the age range for both models was 1–95+ years. Asthma is a “child’ disease that is fit into an overall chronic respiratory disease model. The unadjusted death estimates for asthma are combined with those for chronic obstructive pulmonary disease, interstitial lung disease and pulmonary sarcoidosis, pneumoconiosis, and other chronic respiratory diseases and fit to the distribution of deaths in an overall chronic respiratory disease “parent” model. This results in deaths recorded using non-specific coding systems, such as verbal autopsy, being included in the parent model and redistributed to the child models proportionately.  Vital registration with medical certification of cause of death is a crucial resource for the GBD cause of death analysis. Cause of death data obtained using various revisions of the International Classification of Disease and Injuries (ICD) were mapped to the GBD cause list. Many deaths, however, are assigned to causes that cannot be the underlying cause of death or are inadequately specified. These deaths were reassigned to the most probable underlying causes of death as part of the data processing for GBD. Redistribution algorithms can be divided into three categories: proportionate redistribution, fixed proportion redistribution based on published studies or expert judgment, or statistical algorithms. |
| **Definition for verbal autopsy** | Verbal autopsy (VA) is a method of determining an individuals’ causes of death and cause-specific mortality fractions in populations without a complete vital registration system. Verbal autopsies consist of a trained interviewer using a questionnaire to collect information about the signs, symptoms, and demographic characteristics of a recently deceased person from an individual familiar with the deceased. A standard VA instrument paired with easy-to-implement and effective analytic methods can help bridge significant gaps in information about causes of death, particularly in resource-poor settings. |
| **Severity and years lived with disability** | The distribution between three health states for asthma was derived from an analysis of the USA Medical Expenditure Panel Surveys (MEPS), for which disability weights (DW) had been previously identified. Anyone with a diagnosis of asthma in whom the disability assigned to asthma is negative or zero is asymptomatic. Non-zero values that bin into the three health states assuming a split between these at the midpoint between DW values. In GBD study, the asthma health states were classified into asymptomatic, controlled, partially controlled and uncontrolled levels. The severity levels were multiplied by the severity specific DW values to produce the years lived with disability (YLD). |
| **The calculation of Years of life lost (YLL)** | Years of life lost (YLL) was calculated by multiplying the number of estimated deaths by the standard life expectancy at age of death. |
| **The calculation of disability adjusted life years (DALYs)** | YLD and YLL were summed to produce disability adjusted life years (DALYs). |
| **Estimating uncertainty** | To account for uncertainty in the burden estimates, a total of 1000 draws were estimated, from which the lower and upper bounds of the 95% uncertainty interval (UI) were obtained from the 25th and 975th ranked values. |
| **Definition for high-body mass index** | High body-mass index (BMI) for adults (ages 20+) is defined as BMI greater than 20 to 25 kg/m^2^. High BMI for children (ages 1–19) is defined as being overweight or obese based on International Obesity Task Force standards. |
| **Definition for exposure of smoking** | The prevalence of current smoking and the prevalence of former smoking were estimated using data from cross-sectional nationally representative household surveys. Current smokers were defined as individuals who currently use any smoked tobacco product on a daily or occasional basis. Former smokers were defined as individuals who quit using all smoked tobacco products for at least six months, where possible, or according to the definition used by the survey. |
| **Definition for exposure of occupational asthmagens** | Exposure of occupational asthmagens was estimated for ages 15 and older. Proportion of the working population exposed to asthmagens, based on population distributions across nine occupational categories. |

**Table S2.** Prevalence of asthma in 1990 and 2019 and the percentage change in the age-standardized rates (ASRs) per 100,000, by location

|  | 1990 | | 2019 | | Percentage change in ASRs per 100,000 |
| --- | --- | --- | --- | --- | --- |
| countries and territories | No (95% UI) | ASRs per 100 000 (95% UI) | No (95% UI) | ASRs per 100 000 (95% UI) |  |
| Armenia | 65275.32 (52617.35 to 81444.05) | 1951.76 (1590.37 to 2395.32) | 62215.66 (46975.13 to 76441.97) | 2093.13 (1628.91 to 2624.83) | 7.24 (-6.28 to 16.2) |
| Azerbaijan | 147617.7 (121631.18 to 182985.75) | 2114.05 (1788.84 to 2546.52) | 189910.21 (156275.45 to 234270.89) | 1961.21 (1608.59 to 2435.73) | -7.23 (-13.17 to -0.74) |
| Georgia | 115958.26 (99783.38 to 136628.6) | 2093.3 (1799.08 to 2487.86) | 69581.84 (56805.01 to 85459.17) | 1958.92 (1572.51 to 2475.31) | -6.42 (-17.61 to 6.07) |
| Kazakhstan | 251154.06 (208289.4 to 304227.39) | 1600.12 (1341.65 to 1907.62) | 286976.47 (232366.6 to 356099.44) | 1590.71 (1282.74 to 1958.23) | -0.59 (-9.31 to 7.81) |
| Kyrgyzstan | 140899.04 (117605.42 to 172070.03) | 3441.9 (2937.41 to 4035.55) | 156012.09 (124978.14 to 197290.77) | 2417.2 (1960.71 to 3005.35) | -29.77 (-38.11 to -21.23) |
| Mongolia | 47623.06 (38948.83 to 58904.18) | 2609.96 (2257.19 to 3036.67) | 67113.67 (55071.32 to 82906.07) | 2114.21 (1759.6 to 2568.47) | -18.99 (-25.38 to -12.42) |
| Tajikistan | 122500.61 (98602.28 to 155195.37) | 2610.93 (2233.73 to 3065.59) | 194584.32 (154929.7 to 248712.27) | 2264.74 (1862.91 to 2762.31) | -13.26 (-20.37 to -5.64) |
| Turkmenistan | 100660.65 (84042.36 to 122848.27) | 2936.57 (2566.79 to 3387.63) | 101613.78 (78998.91 to 129040.41) | 2011.28 (1553.21 to 2532.4) | -31.51 (-45.63 to -22.45) |
| Uzbekistan | 737479.54 (617143.73 to 882556.25) | 4267.92 (3652.84 to 4907.67) | 944061.85 (779576.43 to 1162862.71) | 3058.75 (2581.57 to 3661.02) | -28.33 (-34.98 to -21.19) |
| Albania | 79534.76 (66440.33 to 98951.64) | 2613.12 (2252.14 to 3122.14) | 79785.19 (66536.61 to 96602.43) | 2828.38 (2319.68 to 3483.86) | 8.24 (-3.35 to 21.17) |
| Bosnia and Herzegovina | 202585.9 (169114.32 to 245601.78) | 4597.21 (3865.68 to 5553.5) | 161181.22 (134157.15 to 192943.12) | 4466.42 (3656.72 to 5529.45) | -2.85 (-9.02 to 2.56) |
| Bulgaria | 395018.51 (328696.28 to 474830.53) | 4196.23 (3509.42 to 5086.58) | 260802.85 (213949.07 to 311776.07) | 3612.72 (2919.2 to 4427.34) | -13.91 (-21.06 to -5.18) |
| Croatia | 285240.58 (232914.11 to 342261.47) | 5327.11 (4355.52 to 6448.66) | 188078.24 (158810.02 to 223334.43) | 4018.57 (3316.2 to 4929.38) | -24.56 (-31.81 to -18.18) |
| Czechia | 328324.49 (275297.35 to 398491.6) | 3146.19 (2596.81 to 3844.58) | 327052.3 (269708.16 to 392255.67) | 3053.07 (2479.86 to 3781.11) | -2.96 (-9.45 to 2.81) |
| Hungary | 447614.2 (382529.88 to 522250.64) | 4067.31 (3435.01 to 4817.84) | 321229.13 (265613.64 to 385389.84) | 3307.09 (2677.6 to 4083.25) | -18.69 (-24.97 to -11.35) |
| Montenegro | 20386.75 (16548.92 to 25645.08) | 3256.97 (2650.2 to 4102.26) | 22895.84 (18278.45 to 28178.49) | 3620.93 (2883.62 to 4557.22) | 11.18 (2.85 to 20.21) |
| North Macedonia | 135836.51 (105001.39 to 167769.79) | 7017.14 (5386.63 to 8674.96) | 123319.78 (105483.46 to 145172.12) | 5199.79 (4376.14 to 6200.46) | -25.9 (-37.23 to -9.84) |
| Poland | 3982472.18 (3319829.48 to 4735833.69) | 9777.56 (8170.35 to 11707.33) | 2214693.36 (1848569.88 to 2654560.39) | 5412.72 (4413.97 to 6639.11) | -44.64 (-50.32 to -38.71) |
| Romania | 1116655.64 (913030.77 to 1341825) | 4547.38 (3707.62 to 5545.12) | 844316.44 (694481.94 to 1015130.73) | 4179.67 (3402.26 to 5180.64) | -8.09 (-17.36 to 3.47) |
| Serbia | 305521.15 (264297.96 to 356220.48) | 3180.46 (2729.91 to 3769.3) | 260376.53 (224079.91 to 303470.99) | 2849.13 (2358.05 to 3452.56) | -10.42 (-15.69 to -4.89) |
| Slovakia | 159981.33 (133541.99 to 192932.55) | 2996.21 (2487.21 to 3645.75) | 159787.45 (131166.69 to 192791.58) | 2914.98 (2363.25 to 3588.8) | -2.71 (-8.9 to 3.97) |
| Slovenia | 125434.85 (106300.07 to 150286.5) | 5944.55 (5013.1 to 7137.35) | 102604.13 (84761.84 to 123241.22) | 4591.92 (3730.8 to 5677.77) | -22.75 (-29.39 to -16.32) |
| Belarus | 601868.66 (498203 to 710928.52) | 5389.71 (4451.56 to 6393.18) | 328033.37 (271029.94 to 396927.2) | 3417.66 (2760.82 to 4292.83) | -36.59 (-44.05 to -28.86) |
| Estonia | 45503.74 (39890.56 to 51538.75) | 2800.52 (2426.71 to 3210.7) | 26128.41 (21890.75 to 30890.36) | 2075.2 (1663.82 to 2580.6) | -25.9 (-35.44 to -17.18) |
| Latvia | 142340.33 (123130.58 to 163443.26) | 4930.2 (4268.5 to 5758.8) | 60696.29 (51538.88 to 72357.78) | 3184.73 (2584.81 to 4052.73) | -35.4 (-44.31 to -25.22) |
| Lithuania | 126759.67 (107839.06 to 147317.08) | 3288.95 (2775.81 to 3878.73) | 73987 (61789.49 to 88188.76) | 2718.44 (2191.37 to 3363.57) | -17.35 (-26.86 to -5.93) |
| Republic of Moldova | 162782.22 (140214.36 to 193736.88) | 3628.57 (3119.35 to 4341.83) | 98058.45 (78492.61 to 119716.47) | 2750.93 (2200.51 to 3503.81) | -24.19 (-33.48 to -16.97) |
| Russian Federation | 7745719.67 (6534272.78 to 9187026.09) | 4811.5 (4055.71 to 5734.01) | 3507708.44 (2866449.67 to 4254388.34) | 2520.13 (2004.47 to 3174.17) | -47.62 (-53.26 to -41.99) |
| Ukraine | 2517577.91 (2098292.18 to 3020646.41) | 4703.49 (3848.75 to 5741.11) | 1268398.99 (1019017.37 to 1553151.05) | 3227.13 (2528.4 to 4076.43) | -31.39 (-37.76 to -24.97) |
| Australia | 1926397.27 (1794472.82 to 2066599.95) | 11699.48 (10879.25 to 12543.42) | 2047080.58 (1720113.3 to 2448384.04) | 8768.43 (7162.3 to 10821.74) | -25.05 (-37.66 to -9.06) |
| New Zealand | 490464.26 (409341.26 to 591109.98) | 14102.17 (11736.51 to 17001.42) | 270230.52 (226570.44 to 327194.5) | 6325.87 (5156.98 to 7871.32) | -55.14 (-59.09 to -50.36) |
| Brunei Darussalam | 11044.68 (9069.21 to 13800.52) | 5638.87 (4734.3 to 6942.58) | 17726.41 (14630.67 to 21813.5) | 4887.06 (4088.09 to 5908.93) | -13.33 (-22.43 to -7.59) |
| Japan | 11246046.34 (9650456.71 to 12976284.39) | 8192.5 (6971.18 to 9598.36) | 5117128.05 (4314187.03 to 6091158.74) | 3865.42 (3118.67 to 4890.82) | -52.82 (-57.7 to -47.85) |
| Republic of Korea | 1673791.92 (1416284.09 to 2002797.53) | 4491.3 (3824.36 to 5330.33) | 1915846.03 (1611521.12 to 2324501.29) | 3608.56 (2959.47 to 4514.64) | -19.65 (-32.15 to -7.23) |
| Singapore | 188894.13 (156202.02 to 220536.34) | 6704.47 (5653.4 to 7742.12) | 199395.45 (165088.97 to 242228.77) | 3667.15 (3012.13 to 4564.72) | -45.3 (-52.96 to -34.61) |
| Canada | 1424420.5 (1181400.74 to 1715393.55) | 5369.62 (4412.68 to 6548.46) | 1650336.91 (1381362.93 to 1967954.67) | 4817.27 (3892.85 to 5970.94) | -10.29 (-25.18 to -1.93) |
| Greenland | 5033.64 (4016.28 to 5984.42) | 10231.08 (8081.11 to 12146.18) | 3735.74 (3272.19 to 4292.75) | 6702.35 (5767.29 to 7927.96) | -34.49 (-41.67 to -20.75) |
| United States of America | 23477765.41 (20002836.31 to 28076779.08) | 9374.01 (7857.79 to 11294.15) | 33954467.6 (30347008.47 to 38109958.36) | 10399.27 (9140.29 to 11903.19) | 10.94 (2.01 to 21.39) |
| Argentina | 2401216.77 (2056469.5 to 2793029.76) | 7241.79 (6184.77 to 8404.3) | 3234944.5 (2748264.28 to 3848411.19) | 7019.77 (5893.61 to 8448.07) | -3.07 (-12.56 to 7.29) |
| Chile | 654432.97 (558825.09 to 785779.89) | 5329.9 (4616.15 to 6309.12) | 990774.73 (847404.81 to 1160034.8) | 5196.37 (4391.45 to 6249.28) | -2.51 (-10.85 to 6.63) |
| Uruguay | 221567.99 (195395.64 to 251630.33) | 6693.56 (5875.56 to 7703.27) | 205338.76 (178243.04 to 237531.82) | 5663.19 (4778.55 to 6765.04) | -15.39 (-23.37 to -8.41) |
| Andorra | 3821.84 (3174.64 to 4578.72) | 6830.61 (5712.59 to 8190.86) | 5104.38 (4195.79 to 6161.16) | 5846.73 (4810.24 to 7128.01) | -14.4 (-20.63 to -8.74) |
| Austria | 618927.19 (525088.13 to 718197.82) | 7014.85 (5951.85 to 8272.91) | 489835.48 (405030.6 to 590357.05) | 5220.26 (4280.7 to 6380.52) | -25.58 (-33.7 to -16.39) |
| Belgium | 838526.01 (729289.09 to 974389.63) | 7261.55 (6235.65 to 8543.23) | 542550.51 (455999.54 to 645455.86) | 4609.89 (3791.88 to 5606.24) | -36.52 (-44.76 to -26.44) |
| Cyprus | 60638.46 (48930.59 to 73867.6) | 7624.9 (6154.05 to 9289.55) | 99524.74 (80592.5 to 120953.32) | 7088.53 (5756.88 to 8584.47) | -7.03 (-14.52 to 0.5) |
| Denmark | 359686.19 (309203.19 to 417605.44) | 6288.16 (5436.02 to 7394.62) | 282374.8 (236946.48 to 337040.28) | 4782.68 (3910.04 to 5846.14) | -23.94 (-33.17 to -14.26) |
| Finland | 363900.16 (310887.4 to 422891.01) | 6716.65 (5669.76 to 7866.09) | 382167.45 (324211.66 to 446290.84) | 6102.5 (5043.17 to 7355.66) | -9.14 (-16.68 to -2.11) |
| France | 6252763.36 (5566863.67 to 7109419.52) | 9667.56 (8521.8 to 11260) | 4605053.17 (3858023.81 to 5438404.55) | 6555.19 (5357.38 to 7932.96) | -32.19 (-40.02 to -23.15) |
| Germany | 6650125.15 (5702966.34 to 7623159.55) | 7013.11 (6073.46 to 8135.45) | 3609451.99 (3014105.75 to 4299202.51) | 4153.11 (3405.37 to 5081.31) | -40.78 (-49.19 to -31.45) |
| Greece | 690825.72 (577397.27 to 825221.12) | 5885.14 (4858.42 to 7170.64) | 591070.09 (487643.67 to 712681.18) | 5301.47 (4326.18 to 6546.28) | -9.92 (-18.99 to 3.14) |
| Iceland | 24412.19 (20629.3 to 28822.07) | 9533.07 (8020.66 to 11268.75) | 25583.8 (21169.03 to 30770.98) | 7200.09 (5844.36 to 8849.12) | -24.47 (-32.05 to -17.42) |
| Ireland | 376823.07 (323905.49 to 434076.21) | 10213.71 (8764.93 to 11834.99) | 343379.75 (288588.41 to 410323.23) | 6860.25 (5695.04 to 8348.21) | -32.83 (-39.12 to -25.91) |
| Israel | 324288.66 (271489.01 to 382243.53) | 6611.94 (5557.39 to 7744.74) | 465362.11 (392157.35 to 560172.17) | 4958.02 (4134.53 to 6019.91) | -25.01 (-31.24 to -18.53) |
| Italy | 3953861.58 (3330333.66 to 4689030.84) | 5629.27 (4734.78 to 6690.8) | 2561074.29 (2052641.61 to 3143467.01) | 4027.89 (3173.84 to 5106.71) | -28.45 (-37.29 to -18.83) |
| Luxembourg | 38739.11 (32543.95 to 49712.51) | 9298.17 (7741.43 to 11642.58) | 44283.42 (36989.95 to 52364.09) | 6772.13 (5585.75 to 8149.6) | -27.17 (-38.42 to -20.46) |
| Malta | 35646.8 (30501.26 to 41968.58) | 9026.04 (7735.24 to 10612.02) | 35017.81 (29624.47 to 41787.89) | 7075.87 (5876.26 to 8569.64) | -21.61 (-29.85 to -12.35) |
| Monaco | 2033.79 (1720.99 to 2417.64) | 6174.48 (5087.98 to 7532.26) | 2407.32 (2012.49 to 2863.7) | 6014.03 (4939.68 to 7271.8) | -2.6 (-8.06 to 3.37) |
| Netherlands | 1317146.99 (1051807.94 to 1607954.06) | 8134.51 (6444.96 to 10065.64) | 1328831.78 (1109851.89 to 1597935.78) | 6941.99 (5702.57 to 8418.9) | -14.66 (-23.92 to 3.72) |
| Norway | 568647.19 (489979.49 to 666025.34) | 11310.76 (9649.84 to 13320.01) | 372463.04 (309273.72 to 444224.63) | 6790.15 (5589.85 to 8270.13) | -39.97 (-45.61 to -34.45) |
| Portugal | 1261597.8 (1053218.21 to 1506636.89) | 11168.28 (9284.43 to 13524) | 1104293.97 (925175.46 to 1307773.62) | 9106.18 (7499.66 to 11069.6) | -18.46 (-28.08 to -6.34) |
| San Marino | 1442.65 (1197.58 to 1719.46) | 5894.45 (4867.27 to 7102.57) | 1958.54 (1614.93 to 2369.43) | 5759.19 (4728.01 to 7134.22) | -2.29 (-7.97 to 4.17) |
| Spain | 2029684 (1721962.27 to 2420902.16) | 4757.39 (3990.13 to 5809.48) | 2235478.5 (1869134.37 to 2664775.27) | 4481.25 (3636.51 to 5512.17) | -5.8 (-16.6 to 11.01) |
| Sweden | 1190422.1 (999986.29 to 1417070.39) | 11444.22 (9558.65 to 13661.75) | 824426.3 (669881 to 1009468.51) | 7586.11 (6046.56 to 9402.98) | -33.71 (-40.27 to -26.33) |
| Switzerland | 643531.37 (548930.04 to 753372.21) | 8440.95 (7133.54 to 9998.17) | 608821.39 (507685.74 to 733942.75) | 6615.85 (5412.6 to 8052.13) | -21.62 (-30.59 to -11.26) |
| United Kingdom | 8667758.58 (7453176.8 to 10051050.71) | 13998.59 (11939.17 to 16447.44) | 6459572.97 (5468359.22 to 7563833.98) | 9166.57 (7645.04 to 11034.51) | -34.52 (-37.66 to -31.03) |
| Bolivia (Plurinational State of) | 459654.96 (351462.47 to 590008.9) | 5363.51 (4229.61 to 6709.93) | 559829.15 (420622.99 to 732213.98) | 4250.69 (3250.05 to 5536.08) | -20.75 (-30.65 to -10.84) |
| Ecuador | 640099.32 (479396.66 to 830020.13) | 5063.61 (3886 to 6475.25) | 703958.09 (523152 to 942678.03) | 3966.97 (2945.08 to 5332.17) | -21.66 (-33.65 to -8.74) |
| Peru | 1532445.66 (1192069.19 to 1956088.29) | 5669.91 (4486.72 to 7165.46) | 1424251.37 (1057717.12 to 1889702.81) | 4335.62 (3189.83 to 5773.3) | -23.53 (-36.27 to -10.27) |
| Antigua and Barbuda | 3370.34 (2642.27 to 4340.54) | 5338.2 (4266.69 to 6817.64) | 4280.63 (3485.48 to 5300.03) | 5611.62 (4390.4 to 7155.64) | 5.12 (-1.7 to 11.74) |
| Bahamas | 14986.55 (12010.37 to 18586.51) | 5582.22 (4560.77 to 6869.15) | 19052.34 (15600.94 to 23532.56) | 5660.23 (4491.02 to 7161.64) | 1.4 (-6 to 9.5) |
| Barbados | 16885.95 (14246.29 to 20255.61) | 6747.64 (5681.4 to 8152.07) | 17847.41 (14891.75 to 21186.16) | 6878.85 (5508.21 to 8515.22) | 1.94 (-8.59 to 15.09) |
| Belize | 13506.31 (10851.2 to 17142.09) | 6011.39 (5038.07 to 7353.51) | 24260.37 (19296.17 to 30533.96) | 5657.5 (4549 to 7101.45) | -5.89 (-14.73 to 3.17) |
| Bermuda | 2956.22 (2410.15 to 3629.64) | 5534.53 (4390.98 to 6936.55) | 2712.78 (2225.92 to 3334.94) | 5842.79 (4499.49 to 7495.36) | 5.57 (-1.97 to 13.45) |
| Cuba | 808382.77 (661438.61 to 988727.43) | 7590.62 (6224.79 to 9387.2) | 653774.73 (535739.32 to 791659.74) | 6979.37 (5504.92 to 8790.42) | -8.05 (-16.33 to -0.42) |
| Dominica | 5180.02 (4298.03 to 6332.49) | 6708.59 (5684.05 to 8026.61) | 4327.29 (3728.53 to 5081.53) | 6834.37 (5762.73 to 8245.25) | 1.87 (-3.84 to 7.36) |
| Dominican Republic | 328856.44 (267105.37 to 407416.83) | 3933.48 (3275.5 to 4743.56) | 420153.01 (335178.4 to 527757.42) | 3858.57 (3093.28 to 4851.15) | -1.9 (-12.32 to 9.72) |
| Grenada | 6601.13 (5224.1 to 8351.28) | 7094.83 (5720.49 to 8789.49) | 6767.89 (5433.89 to 8402.12) | 7097.17 (5663.9 to 8994.22) | 0.03 (-7.49 to 7.68) |
| Guyana | 55001 (45326.94 to 67074.78) | 6701.1 (5741.22 to 7946.06) | 48079.49 (39843.07 to 58262.83) | 6206.78 (5127.47 to 7539.04) | -7.38 (-13.49 to -0.78) |
| Haiti | 616839.17 (513397.64 to 746777.58) | 8218.27 (7092.08 to 9611.38) | 944674.36 (796000.15 to 1137376.42) | 7086.45 (6059.95 to 8372.99) | -13.77 (-18.88 to -8.76) |
| Jamaica | 175081.04 (142442.3 to 218619.49) | 6714.03 (5616.66 to 8214.99) | 146410.93 (117835.58 to 181759.27) | 5691.56 (4485.71 to 7172.32) | -15.23 (-24.05 to -6.82) |
| Puerto Rico | 389421.17 (326049.78 to 461382.56) | 10828.83 (9074.55 to 12841.4) | 243455.36 (202295.95 to 292121.1) | 8571.06 (6736.88 to 10699.94) | -20.85 (-28.7 to -13.02) |
| Saint Kitts and Nevis | 2662.47 (2124.16 to 3363.98) | 6109.12 (5012.31 to 7552.67) | 2896.81 (2322.21 to 3581.57) | 5647.33 (4364.73 to 7149.5) | -7.56 (-15.72 to 0.14) |
| Saint Lucia | 10660.88 (8564.62 to 13294.22) | 7234.42 (6046.95 to 8719.05) | 10517.69 (8965.54 to 12359.81) | 6751.58 (5593.62 to 8238.9) | -6.67 (-13.73 to 0.19) |
| Saint Vincent and the Grenadines | 7149.55 (5696.39 to 9034.95) | 5883.44 (4840.7 to 7248.07) | 5879.16 (4873.16 to 7270.72) | 5703.08 (4605.61 to 7224.47) | -3.07 (-9.4 to 3.8) |
| Suriname | 23141.68 (18871.45 to 28886.97) | 5557.84 (4633.21 to 6790.95) | 29416.68 (23781.49 to 36861.01) | 5360.3 (4298 to 6764.45) | -3.55 (-11.22 to 4.26) |
| Trinidad and Tobago | 54227.44 (43661.12 to 67448.97) | 4189.31 (3457.37 to 5149.25) | 54667.26 (43022.62 to 68099.75) | 4631.31 (3578.78 to 5962.65) | 10.55 (-6.22 to 24.77) |
| United States Virgin Islands | 5386.72 (4343.43 to 6764.22) | 5019.94 (4065.55 to 6267.63) | 4723.95 (3918.71 to 5750.2) | 5366.08 (4255.76 to 6754.18) | 6.9 (0.49 to 13.5) |
| Colombia | 1605880.46 (1267081.07 to 2032067.95) | 4826.13 (3977.63 to 5909.6) | 1551387.66 (1228472.17 to 1944967.83) | 3478.04 (2729.24 to 4428.09) | -27.93 (-34.95 to -20.41) |
| Costa Rica | 220712.53 (177445.37 to 271958.25) | 6971.38 (5804.34 to 8480.04) | 246651.56 (200613.17 to 304693.94) | 5529.27 (4443.27 to 6905.54) | -20.69 (-29.48 to -11.83) |
| El Salvador | 524413.36 (400755.42 to 667852.23) | 8741.65 (6849.07 to 10792.19) | 337602.54 (272860.39 to 419278.49) | 5419.43 (4395.27 to 6674.38) | -38 (-46.99 to -25.44) |
| Guatemala | 529027.38 (427158.17 to 658829.84) | 5135.94 (4304.93 to 6155.73) | 547911.79 (420000.63 to 720466.57) | 2865.85 (2258.38 to 3708.11) | -44.2 (-52.78 to -34.73) |
| Honduras | 338106.89 (271542.86 to 422419.05) | 5433.45 (4551.69 to 6564.12) | 404373.93 (317883.87 to 517982.07) | 3778.05 (3053.69 to 4714.63) | -30.47 (-38.33 to -21.89) |
| Mexico | 3193956.18 (2488867.75 to 4169496.37) | 3534.94 (2901.91 to 4376.13) | 3353354.64 (2591663.45 to 4377728.91) | 2765.09 (2128.83 to 3625.99) | -21.78 (-28.6 to -14.4) |
| Nicaragua | 288604.72 (222269.84 to 367502.26) | 5784.44 (4734.52 to 7081.98) | 255250.58 (199175.89 to 328145.8) | 3790.58 (3008.67 to 4812.98) | -34.47 (-43.18 to -25.5) |
| Panama | 142096.72 (116904.31 to 175401.23) | 5396.86 (4514.54 to 6576.13) | 182693.23 (145350.09 to 229023.69) | 4431.83 (3509.89 to 5574.74) | -17.88 (-28.07 to -6.3) |
| Venezuela (Bolivarian Republic of) | 999661.36 (785216.42 to 1272634.47) | 4672.99 (3795.94 to 5791.31) | 1041985.13 (829836 to 1304105.1) | 3882.41 (3058.39 to 4893.49) | -16.92 (-25.34 to -8.12) |
| Brazil | 9894735.31 (7559903.35 to 13161901.17) | 6045.9 (4725.06 to 7807.75) | 9520210.56 (7444049.49 to 12180750.27) | 4892.43 (3757.28 to 6352.04) | -19.08 (-25.47 to -13.46) |
| Paraguay | 219130.76 (170123.8 to 288666.23) | 4583.57 (3700.19 to 5780.54) | 375953.23 (293749.42 to 490425.32) | 5408.15 (4227.82 to 7017.87) | 17.99 (8.75 to 26.88) |
| Afghanistan | 498370.57 (428704.74 to 582821.38) | 4740.1 (4210.69 to 5299.51) | 1515531.73 (1250583.77 to 1877887.06) | 4281.02 (3758.84 to 4909.04) | -9.69 (-14.34 to -4.9) |
| Algeria | 777902.78 (640644.41 to 976465.89) | 3171.04 (2723.35 to 3752.87) | 1311914.59 (1073558.12 to 1613359.19) | 3219.45 (2632.92 to 3923.89) | 1.53 (-8.23 to 11.51) |
| Bahrain | 19611.07 (16473.49 to 23652.58) | 4485.51 (3915.71 to 5195.32) | 46332.6 (38382.26 to 56216.15) | 3639.21 (2998.99 to 4452.81) | -18.87 (-26.38 to -11.6) |
| Egypt | 2023076.91 (1676916.35 to 2471492.65) | 3697.98 (3170.86 to 4332.3) | 3347076.33 (2743235.62 to 4121161.6) | 3486.8 (2903.16 to 4200.21) | -5.71 (-13.35 to 1.1) |
| Iran (Islamic Republic of) | 2298638.93 (1871633.97 to 2861152.27) | 4235.23 (3602.37 to 5011.99) | 2635572.9 (2182430.09 to 3205593.56) | 3280.14 (2717.29 to 3978.44) | -22.55 (-26.66 to -18.05) |
| Iraq | 818212.63 (685130.98 to 1010795.3) | 5006.8 (4413.91 to 5753.84) | 1451875.53 (1169492.13 to 1834319.03) | 3446.54 (2875.1 to 4218.2) | -31.16 (-37.83 to -24.21) |
| Jordan | 155570.98 (121602.37 to 199268.72) | 4273.34 (3560.58 to 5114.41) | 470579.07 (373464.89 to 600637.68) | 4044.69 (3266.12 to 5037.91) | -5.35 (-13.24 to 5.32) |
| Kuwait | 74040.51 (59756.26 to 91926.98) | 4470.85 (3734.58 to 5337.94) | 174108.87 (138549.35 to 221830.84) | 4174.46 (3290.55 to 5265.12) | -6.63 (-18.15 to 6.18) |
| Lebanon | 143799.58 (119374.79 to 172410.86) | 4514.71 (3828.82 to 5299.21) | 227323.47 (186844.61 to 274255.63) | 4443.27 (3641.77 to 5385.77) | -1.58 (-10.04 to 7.92) |
| Libya | 167768.95 (136989.7 to 209553.25) | 4259.24 (3718.16 to 4984.94) | 235518.58 (199455.83 to 282297.43) | 3725.34 (3122.59 to 4497.03) | -12.54 (-17.88 to -6.98) |
| Morocco | 665779.51 (550849.77 to 810007.77) | 2675.66 (2312.12 to 3124.72) | 1020288.24 (859392.6 to 1228506.91) | 2914.58 (2452.51 to 3485.52) | 8.93 (0.82 to 16.63) |
| Oman | 60553.27 (48051.22 to 78424.78) | 3084.05 (2504.79 to 3817.47) | 172074.98 (136810.21 to 218228.73) | 4053.95 (3279.06 to 5014.45) | 31.45 (19.2 to 49.62) |
| Palestine | 72287.08 (57156.67 to 93316.71) | 3654.66 (3083.74 to 4340.73) | 167758.89 (130859.35 to 216934.67) | 3332.38 (2715.94 to 4088.46) | -8.82 (-16.35 to -1.51) |
| Qatar | 14085.92 (11478.68 to 17566.49) | 3410.6 (2829.31 to 4117.75) | 84800.06 (67145.66 to 108244.68) | 3460.53 (2793.84 to 4306.11) | 1.46 (-7.06 to 9.79) |
| Saudi Arabia | 344118.61 (275108.38 to 439247.72) | 2092.99 (1765.37 to 2507.05) | 853996.12 (705952.38 to 1027815.08) | 2642.17 (2154.91 to 3207.34) | 26.24 (16.54 to 40.04) |
| Sudan | 843167.68 (682566.22 to 1031843.98) | 4326.31 (3687.08 to 5084.28) | 1626704.75 (1297906.32 to 2051809.59) | 4174.67 (3493.15 to 4976.25) | -3.51 (-11.07 to 4.45) |
| Syrian Arab Republic | 430804.22 (356993.17 to 525828.34) | 3348.28 (2925.57 to 3861.47) | 469398.58 (397788.76 to 559452.72) | 3268.89 (2774.54 to 3868.76) | -2.37 (-8.84 to 4.68) |
| Tunisia | 290735.64 (241635.77 to 356962.57) | 3603.34 (3107.95 to 4296.02) | 412522.29 (348081.55 to 491378.82) | 3640.19 (3020.49 to 4393.76) | 1.02 (-5.81 to 8.77) |
| Turkey | 3029325.77 (2586991.08 to 3670399.65) | 5503.39 (4813.36 to 6430.52) | 4082369.92 (3517473.53 to 4743626.7) | 5102.54 (4401.62 to 6040.67) | -7.28 (-14.14 to -0.81) |
| United Arab Emirates | 126457.42 (105116.48 to 150912.72) | 8100.42 (6883.34 to 9295.82) | 614690.18 (514497.66 to 719007.06) | 7179.91 (6188.25 to 8345.37) | -11.36 (-16.88 to -4.38) |
| Yemen | 599901.01 (498756.4 to 737931.28) | 4807.31 (4277.85 to 5430.54) | 1189810.06 (968154.28 to 1487502.54) | 3946.64 (3401.3 to 4620.19) | -17.9 (-23.08 to -12.28) |
| Bangladesh | 1522762.57 (1338901.97 to 1747547.24) | 1855.79 (1667.22 to 2057.79) | 2073478.31 (1801990.97 to 2362799.75) | 1390.91 (1217.24 to 1574.2) | -25.05 (-29.62 to -19.71) |
| Bhutan | 9816.36 (8567.18 to 11265.61) | 2022.18 (1811.26 to 2239.02) | 9763.37 (8608.13 to 11051.01) | 1429.78 (1272.34 to 1603.04) | -29.3 (-34.09 to -23.97) |
| India | 20333262.35 (17097405.64 to 24464734.37) | 2970.59 (2519.45 to 3502.25) | 34305973.24 (27950714.4 to 41323723.48) | 2680.88 (2189.55 to 3221.55) | -9.75 (-16.71 to -6.19) |
| Nepal | 191969.16 (168074.85 to 220532.28) | 1254.04 (1117.27 to 1405.47) | 289922.63 (249850.52 to 332070.17) | 1072.46 (932.39 to 1214.78) | -14.48 (-19.66 to -9.13) |
| Pakistan | 1982622.89 (1669902.73 to 2383032.88) | 2133.26 (1825.96 to 2475.09) | 3192551.52 (2659807 to 3848337.33) | 1702.08 (1448.11 to 1974.78) | -20.21 (-23.15 to -16.68) |
| China | 25559015.07 (20406449.83 to 32633237.64) | 2296.63 (1852.1 to 2880.73) | 24766952.29 (20076066.34 to 30666151.01) | 1974.16 (1530.16 to 2565.37) | -14.04 (-18.41 to -10.2) |
| Democratic People's Republic of Korea | 763485.74 (628963.73 to 963120.81) | 3852.07 (3195.86 to 4916.22) | 998397.84 (880328.47 to 1140796.98) | 3704.71 (3216.47 to 4372.58) | -3.83 (-21.64 to 5.26) |
| Taiwan (Province of China) | 796766.34 (670325.13 to 980823.65) | 4235.82 (3579.83 to 5155.33) | 737208.52 (628777.96 to 879469.15) | 3385.72 (2740.55 to 4296.11) | -20.07 (-31.69 to -4.45) |
| American Samoa | 1655.29 (1320.1 to 2108.87) | 3486.46 (2789.18 to 4263.99) | 1852.81 (1276.54 to 2350.73) | 3340.52 (2255.05 to 4204.59) | -4.19 (-21.66 to 11.41) |
| Cook Islands | 754.29 (563.02 to 969.97) | 3996.52 (2926.74 to 5014.24) | 637.59 (422.16 to 785.69) | 3582.17 (2502.86 to 4533.59) | -10.37 (-17.17 to 3.99) |
| Fiji | 31450.47 (27141.95 to 36539.03) | 4795.31 (4209.92 to 5431.4) | 29195.57 (25127.87 to 34144.14) | 3289.09 (2847.38 to 3816.31) | -31.41 (-36.07 to -26.6) |
| Guam | 5264.87 (3871.66 to 6708.72) | 3991.59 (2848.07 to 5007.12) | 5679.99 (3905.12 to 7189.15) | 3380.03 (2381.97 to 4343.26) | -15.32 (-20.51 to -10.01) |
| Kiribati | 4573.59 (4159.41 to 5041.39) | 6715.19 (6192.73 to 7250.43) | 5276.8 (4787.32 to 5860.92) | 4759.28 (4377.4 to 5201.51) | -29.13 (-32.09 to -25.9) |
| Marshall Islands | 1646.62 (1392.66 to 1984.82) | 3894.18 (3513.52 to 4360.85) | 1682.23 (1382.93 to 2062.22) | 3015.2 (2495.91 to 3682.02) | -22.57 (-32.29 to -11.27) |
| Micronesia (Federated States of) | 3763.61 (3245.74 to 4444.47) | 3883.06 (3497.07 to 4330.61) | 3139.83 (2583.01 to 3835.55) | 3144.08 (2602.21 to 3828.47) | -19.03 (-29.15 to -7.36) |
| Nauru | 345.13 (286.51 to 434.05) | 3479.47 (2998.37 to 4127.4) | 340.03 (260.52 to 451.66) | 3289.83 (2534.29 to 4239.2) | -5.45 (-23.28 to 10.35) |
| Niue | 86.42 (67.78 to 110.14) | 3569.06 (2794.22 to 4479.16) | 58.8 (38.92 to 73.09) | 3507.34 (2425.36 to 4402.84) | -1.73 (-16.84 to 10.21) |
| Northern Mariana Islands | 1661.99 (1300.35 to 2101.11) | 4054.57 (3137.65 to 5061.41) | 1414.15 (939.52 to 1750.38) | 3574.83 (2623.32 to 4576.91) | -11.83 (-20.5 to 3.75) |
| Palau | 601.46 (497.88 to 722.68) | 4096.5 (3422.99 to 4835.41) | 573.17 (460.04 to 675.53) | 3321.08 (2719.29 to 4038.01) | -18.93 (-22.85 to -13.66) |
| Papua New Guinea | 217930.58 (193532.13 to 247824.7) | 6044.03 (5460.11 to 6634.91) | 412884.08 (365291.58 to 465899.4) | 4585.56 (4143.28 to 5043.65) | -24.13 (-27.15 to -20.84) |
| Samoa | 5475.93 (4503.16 to 6712.58) | 3535.46 (3027.18 to 4102.23) | 6240.9 (5020.99 to 7809.78) | 2953.1 (2413.03 to 3599.31) | -16.47 (-24.04 to -4.98) |
| Solomon Islands | 12058.84 (10101.81 to 14652.48) | 3903.98 (3445.61 to 4493.34) | 22073.88 (17930.94 to 27938.58) | 3471.97 (2872 to 4273.75) | -11.07 (-21.25 to -0.77) |
| Tokelau | 59.24 (44.51 to 80.62) | 3385.19 (2567.81 to 4566.16) | 49.54 (34.97 to 63.87) | 3426.61 (2381.94 to 4338.56) | 1.22 (-17.67 to 28.62) |
| Tonga | 4216.86 (3428.58 to 5331.44) | 4503.5 (3827.75 to 5376.12) | 3798.06 (3041.75 to 4782.14) | 3661.38 (2980.68 to 4537.51) | -18.7 (-25.59 to -6.05) |
| Tuvalu | 286.4 (243.24 to 341.88) | 3107.53 (2681.79 to 3649.81) | 395.77 (271.91 to 504.49) | 3395.45 (2319.94 to 4292.1) | 9.27 (-21.63 to 27.79) |
| Vanuatu | 4901.75 (4120.92 to 5868.94) | 3425.91 (3019.57 to 3884.87) | 8924.47 (6535.11 to 11928.9) | 3047.07 (2203.69 to 3990.17) | -11.06 (-33.03 to 8.83) |
| Cambodia | 227574.92 (184184.9 to 283855.36) | 2172.79 (1884.4 to 2526.42) | 386095.49 (322587.17 to 474534.85) | 2403.29 (2034.03 to 2896.1) | 10.61 (3.94 to 18.24) |
| Indonesia | 6702563.41 (5644398.8 to 8162426.35) | 3811.87 (3282.97 to 4515.18) | 8673884.37 (7367856.05 to 10270736.17) | 3431.25 (2918.28 to 4055.37) | -9.99 (-13.43 to -6.05) |
| Lao People's Democratic Republic | 103986.3 (87489.02 to 126244.27) | 2401.57 (2100.24 to 2774) | 147308.67 (126745.94 to 175671.3) | 2140.92 (1867.95 to 2502.13) | -10.85 (-15.98 to -4.58) |
| Malaysia | 531063.9 (460610.38 to 622801.25) | 3348.11 (2966.97 to 3795.64) | 792596.05 (662894.81 to 969016.49) | 2624.7 (2178.25 to 3253.87) | -21.61 (-30.68 to -11.17) |
| Maldives | 8980.32 (7529.09 to 10819.73) | 3624.4 (3167.89 to 4154.63) | 11017.6 (8811.18 to 13954.64) | 2422.72 (1927.16 to 3083.75) | -33.16 (-43.66 to -21.1) |
| Mauritius | 42203.76 (36763.25 to 49125.03) | 4222.42 (3732.13 to 4835.53) | 33596.2 (29237.54 to 39278.13) | 2774.03 (2334.5 to 3376.84) | -34.3 (-40.21 to -27.39) |
| Myanmar | 843885.34 (731074.28 to 992833.36) | 2178.29 (1935.93 to 2475.64) | 1065997.14 (933365.5 to 1212653.04) | 2016.24 (1770.17 to 2293.06) | -7.44 (-12.18 to -2.8) |
| Philippines | 3950818.07 (3223138.49 to 4989880.7) | 5870.1 (4962.4 to 7115.66) | 5218947.6 (4372997.03 to 6402826.38) | 4628.31 (3916.19 to 5595.11) | -21.15 (-23.09 to -19.24) |
| Seychelles | 1784.58 (1491.63 to 2188.36) | 2476.52 (2120.59 to 2959.51) | 2394.21 (2041.18 to 2850.16) | 2475.75 (2052.28 to 3052.36) | -0.03 (-5.72 to 5.7) |
| Sri Lanka | 537048.68 (470010.91 to 619235.73) | 3611.24 (3208.86 to 4041.89) | 799525.86 (705681.51 to 914751.36) | 3541.69 (3091.39 to 4114.25) | -1.93 (-8.7 to 5.61) |
| Thailand | 2009331.32 (1734418.24 to 2354719.17) | 3917.91 (3442.68 to 4486) | 2309630.86 (2005711.93 to 2688542.41) | 3411.53 (2863.7 to 4141.62) | -12.92 (-21.15 to -4.21) |
| Timor-Leste | 26324.73 (21879.23 to 31618.41) | 3155.12 (2752.16 to 3619.85) | 39728.74 (33709.86 to 47060.69) | 2904.97 (2544.44 to 3332.76) | -7.93 (-13.79 to -1.86) |
| Viet Nam | 1708254.81 (1411737.44 to 2146063.25) | 2603.15 (2231.71 to 3086.15) | 2849226.88 (2429245.31 to 3358420.3) | 3092.64 (2613.07 to 3713.87) | 18.8 (13.14 to 25.63) |
| Angola | 473611.72 (399473.89 to 576302.1) | 4472.68 (3958.66 to 5120.28) | 987366.31 (784258.65 to 1232058.17) | 3140.14 (2694.02 to 3682.62) | -29.79 (-35.44 to -24.1) |
| Central African Republic | 118620.82 (100637.41 to 141253.05) | 4409.86 (3923.28 to 4972.66) | 183038.99 (152908.8 to 221750.73) | 3476.77 (3045.21 to 3965.72) | -21.16 (-24.91 to -17.39) |
| Congo | 107610.94 (89540.05 to 130973.05) | 4496.14 (3934.29 to 5172.43) | 191763.58 (156525.21 to 236966.27) | 3717.56 (3167.36 to 4387.34) | -17.32 (-22.91 to -11.59) |
| Democratic Republic of the Congo | 1360067.62 (1097868.42 to 1692027.14) | 3342.79 (2865.73 to 3903.78) | 2664393.15 (2183111.66 to 3293445.5) | 3003.45 (2582.79 to 3545.38) | -10.15 (-15.03 to -4.13) |
| Equatorial Guinea | 17469.8 (14646.88 to 21189.94) | 4004.75 (3518.47 to 4559.11) | 43537.12 (33747.21 to 55835.49) | 3027.96 (2529.24 to 3664.17) | -24.39 (-31.42 to -17.12) |
| Gabon | 33821.97 (28052.94 to 41412.78) | 3454.6 (2986.13 to 4055.44) | 47723.65 (38512.82 to 60949.61) | 2736.53 (2258.42 to 3395.03) | -20.79 (-27.25 to -13.96) |
| Burundi | 344826.75 (289563.61 to 413622.01) | 6006.62 (5296 to 6776.02) | 517185 (419705.08 to 636973.05) | 4142.85 (3585.48 to 4806.9) | -31.03 (-35.39 to -26.88) |
| Comoros | 23463.84 (19700.93 to 28536.41) | 4807.4 (4195.46 to 5604.7) | 29194.43 (24318.45 to 35463.54) | 4061.88 (3450.4 to 4841.62) | -15.51 (-22.24 to -9.21) |
| Djibouti | 26428.29 (21824.44 to 32454.73) | 5245.52 (4563.13 to 6057) | 48835 (40329.59 to 59711.67) | 4132.74 (3519.31 to 4912.78) | -21.21 (-26.78 to -15.1) |
| Eritrea | 161786.12 (135703.96 to 194609.77) | 5360.57 (4737.84 to 6074.41) | 294185.24 (245718.89 to 357430.21) | 4403.82 (3837.02 to 5096.06) | -17.85 (-22.33 to -12.87) |
| Ethiopia | 1989810.91 (1603190.3 to 2509502.94) | 3780.55 (3205.88 to 4489.35) | 3249237.34 (2524663.64 to 4200802.33) | 2791.61 (2299.35 to 3414.23) | -26.16 (-31.31 to -20.19) |
| Kenya | 775051.74 (610439.96 to 1000262.55) | 3221.24 (2698.51 to 3856.04) | 1418419.53 (1122229.98 to 1798199.77) | 2730.22 (2259.15 to 3321.32) | -15.24 (-17.5 to -12.65) |
| Madagascar | 1321024 (1136958.18 to 1544897.14) | 9951.56 (8841.92 to 11236.94) | 1735900.5 (1490585.3 to 2034248.23) | 6355.94 (5633.56 to 7165.78) | -36.13 (-40.33 to -31.41) |
| Malawi | 412315.29 (330978.96 to 519276.28) | 4023.52 (3426 to 4706) | 719451.02 (565656.58 to 901923.61) | 3707.18 (3090.3 to 4431.57) | -7.86 (-13.56 to -1.75) |
| Mozambique | 622611.22 (515046.26 to 768083.06) | 4301.37 (3745.85 to 5011.2) | 1329709.36 (1075478 to 1671937.92) | 4312.89 (3736.06 to 5066.18) | 0.27 (-5.91 to 7.02) |
| Rwanda | 869272.03 (723189.36 to 1040145.11) | 12041.83 (10348.66 to 13779.87) | 1013743.15 (843674.99 to 1238599.32) | 8203.91 (7081.25 to 9665.99) | -31.87 (-37.41 to -24.99) |
| Somalia | 428872.62 (360946.47 to 524616.66) | 5833.37 (5168.39 to 6666.08) | 917607.83 (755094.8 to 1135565.59) | 4452.97 (3914.46 to 5133.05) | -23.66 (-27.67 to -19.25) |
| South Sudan | 295384.01 (244982.3 to 362764.25) | 4839.06 (4210.63 to 5632.84) | 389614.89 (313999.5 to 490359.63) | 3963.92 (3389.98 to 4680.27) | -18.08 (-22.7 to -12.62) |
| Uganda | 1060938.68 (864544.66 to 1333995.03) | 5822.26 (5049.92 to 6843.37) | 2136098.96 (1682331.38 to 2750153.02) | 4952.62 (4216.71 to 5881.59) | -14.94 (-20.69 to -8.74) |
| United Republic of Tanzania | 1661671.43 (1340859.91 to 2072273.46) | 6189.98 (5266.16 to 7300.72) | 3509358.14 (2815093.18 to 4429075.62) | 5923.46 (5047.86 to 7127.75) | -4.31 (-9.58 to 1.22) |
| Zambia | 199390.68 (160973.04 to 250480.56) | 2340.79 (2011.5 to 2747.57) | 459061.03 (353763.21 to 602972) | 2304.07 (1889.08 to 2844.53) | -1.57 (-9.69 to 8.32) |
| Botswana | 28782.45 (24204.29 to 34737.64) | 2497.09 (2199.84 to 2832.94) | 56625.75 (46318.66 to 68640.16) | 2572.75 (2165.53 to 3052.39) | 3.03 (-6.67 to 18.54) |
| Eswatini | 34985.72 (29498.44 to 42263.95) | 5161.17 (4529.13 to 5816.13) | 46773.81 (40350.44 to 54756.12) | 4646.92 (4078.1 to 5220.68) | -9.96 (-14.21 to -5.43) |
| Lesotho | 22888.83 (18971.07 to 27417.57) | 1341.01 (1162.03 to 1530.05) | 27207.85 (23277.1 to 31479.79) | 1377.07 (1185.73 to 1567.73) | 2.69 (-3.01 to 8.82) |
| Namibia | 31445.36 (26714 to 37669.41) | 2489.33 (2191.25 to 2850.52) | 47132.27 (39200.31 to 57783.35) | 2096.89 (1800.72 to 2468.94) | -15.77 (-20.61 to -9.68) |
| South Africa | 1796364.16 (1181000.02 to 2313975.33) | 4970.22 (3355.21 to 6224.02) | 2219861.11 (1485832.27 to 2910877.45) | 4123.97 (2787.98 to 5399.18) | -17.03 (-24.95 to -9.79) |
| Zimbabwe | 173744 (140920.54 to 218376.31) | 1915.39 (1669.6 to 2217.42) | 265193.6 (219778.02 to 321809.06) | 1989.98 (1728.06 to 2286.69) | 3.89 (-0.87 to 8.73) |
| Benin | 149530.81 (123694.96 to 182223.6) | 3241.92 (2874.74 to 3694.52) | 345397.36 (275120.87 to 436253.89) | 2783.41 (2387.15 to 3274.67) | -14.14 (-19.6 to -8.73) |
| Burkina Faso | 265313.15 (212800.06 to 336829.94) | 2595.47 (2228.41 to 3056.79) | 615592.24 (487309.83 to 799997.12) | 2586.12 (2178.5 to 3125.76) | -0.36 (-5.91 to 5.65) |
| Cabo Verde | 10690.69 (8686.23 to 13394.89) | 3046.64 (2631.55 to 3583.54) | 13527.32 (11060.66 to 16913.14) | 2443.63 (2020.5 to 3024.72) | -19.79 (-26.29 to -12.58) |
| Cameroon | 262618.77 (213035.99 to 328472.94) | 2694.41 (2310.95 to 3168.33) | 679954.66 (536380.75 to 880881.21) | 2325.79 (1937.92 to 2835.75) | -13.68 (-20.29 to -6.76) |
| Chad | 137722 (112144.46 to 172438.32) | 2320.1 (2009.53 to 2722.77) | 371908.16 (292030.36 to 479771.78) | 2161.69 (1838.47 to 2574.23) | -6.83 (-12.29 to -0.58) |
| Côte d'Ivoire | 394613.39 (324190.83 to 487962.24) | 3450.94 (2993.9 to 4035.39) | 805616.74 (658269.84 to 1010962.43) | 3154.29 (2690.66 to 3760.81) | -8.6 (-14.2 to -2.73) |
| Gambia | 29420.07 (23910.29 to 36443.66) | 3217.6 (2822.5 to 3745.44) | 60977.98 (49112.4 to 76401) | 2791.54 (2399.96 to 3300.47) | -13.24 (-18.09 to -8.14) |
| Ghana | 314077.05 (250560.43 to 395455.98) | 2037.4 (1731.91 to 2412.07) | 667512.45 (541607.55 to 840813.14) | 2118.82 (1785.69 to 2580.9) | 4 (-1.85 to 9.51) |
| Guinea | 224458.17 (183774.61 to 275830.03) | 3690.27 (3154.77 to 4346.61) | 422768.58 (336843.96 to 536670.96) | 3372.99 (2835.33 to 3999.13) | -8.6 (-14.83 to -2.14) |
| Guinea-Bissau | 35844.14 (30228.04 to 43000.38) | 3917.06 (3485.23 to 4395.86) | 56298.83 (46912.35 to 68779.57) | 3123.22 (2760.66 to 3618.12) | -20.27 (-24.93 to -14.77) |
| Liberia | 59315.62 (48521.74 to 74831.34) | 2714.1 (2351.71 to 3235.39) | 115411.8 (91514.49 to 147961.65) | 2334.41 (1955.58 to 2853) | -13.99 (-21.94 to -7.39) |
| Mali | 180947.95 (148099.37 to 227712.19) | 2128.29 (1835.48 to 2502.46) | 480574.85 (382959.55 to 628685.16) | 2140.49 (1829 to 2567.68) | 0.57 (-4.68 to 6.13) |
| Mauritania | 105881.61 (86144.99 to 128860.73) | 5666.62 (4812.32 to 6590.38) | 180652.76 (147063.4 to 225869.38) | 4687.13 (4033.79 to 5506.4) | -17.29 (-24.03 to -8.49) |
| Niger | 273988.86 (227371.88 to 340374.06) | 3410.18 (3015.83 to 3942.99) | 654110 (519223.01 to 840417.61) | 2713.05 (2339 to 3202.37) | -20.44 (-25.65 to -14.89) |
| Nigeria | 4054611.68 (3334022.5 to 5015410.68) | 4727.22 (4045.73 to 5589.32) | 7720069.73 (6110404.59 to 9942613.83) | 3626.95 (3065.9 to 4365.72) | -23.28 (-26.2 to -20.03) |
| Sao Tome and Principe | 6002.69 (5011.85 to 7136.87) | 4980.73 (4394.4 to 5641.56) | 8040.64 (6908.4 to 9563.02) | 4253.65 (3782.33 to 4849.6) | -14.6 (-19.98 to -8.54) |
| Senegal | 186393.8 (153562.55 to 232839.86) | 2517.06 (2183.9 to 2939.24) | 348821.54 (278480.62 to 440631.45) | 2318.17 (1960.09 to 2779.97) | -7.9 (-14.75 to -1.92) |
| Sierra Leone | 117405.89 (98452.3 to 141759.5) | 3188.27 (2825.33 to 3660.66) | 229112.23 (187283.75 to 285127.83) | 2768.06 (2393.95 to 3284.09) | -13.18 (-18.64 to -7.25) |
| Togo | 142993.16 (115812.47 to 178026.89) | 4210.07 (3608.03 to 4910.99) | 276092.2 (225766.32 to 346221.85) | 3616.65 (3068.84 to 4309.65) | -14.1 (-20.53 to -6.69) |

**Table S3.** Deaths of asthma in 1990 and 2019 and the percentage change in the age-standardized rates (ASRs) per 100,000, by location

|  | 1990 | | 2019 | | Percentage change in ASRs per 100,000 |
| --- | --- | --- | --- | --- | --- |
| countries and territories | No (95% UI) | ASRs per 100 000 (95% UI) | No (95% UI) | ASRs per 100 000 (95% UI) |  |
| Armenia | 58.67 (43.32 to 74.15) | 2.41 (1.76 to 3.08) | 17.97 (13.8 to 23.04) | 0.45 (0.35 to 0.57) | -81.48 (-87.48 to -72.73) |
| Azerbaijan | 448.55 (352.11 to 596.37) | 9.67 (7.43 to 13.23) | 364.22 (259.4 to 591.47) | 5.34 (3.57 to 8.5) | -44.81 (-71.4 to -9.35) |
| Georgia | 375.62 (309.17 to 446.08) | 6.65 (5.49 to 7.89) | 115.97 (90.85 to 156.14) | 1.94 (1.54 to 2.54) | -70.86 (-77.25 to -62.24) |
| Kazakhstan | 1333.17 (1015.37 to 1737.05) | 11.44 (8.66 to 15.07) | 1259.48 (928.99 to 1611.59) | 8.44 (6.2 to 10.86) | -26.23 (-43.74 to 2.57) |
| Kyrgyzstan | 155.95 (122.35 to 190.72) | 5.13 (3.99 to 6.3) | 57.06 (45.94 to 80.59) | 1.32 (1.08 to 1.85) | -74.22 (-80.79 to -56.83) |
| Mongolia | 152.83 (112.94 to 199.3) | 16.78 (12.42 to 22.24) | 105.03 (77.55 to 154.2) | 6.31 (4.7 to 9.05) | -62.39 (-74.7 to -38.82) |
| Tajikistan | 380.1 (254.27 to 554.18) | 14.15 (9.38 to 20.87) | 250.06 (191.44 to 327.13) | 9.39 (6.74 to 12.16) | -33.61 (-58.35 to -2.07) |
| Turkmenistan | 205.77 (145.74 to 260.12) | 10.64 (7.56 to 13.73) | 49.24 (32.96 to 92.56) | 1.31 (0.87 to 2.55) | -87.68 (-92.11 to -71.36) |
| Uzbekistan | 1363.59 (924.56 to 1716.27) | 12.57 (8.27 to 16.04) | 1123.06 (885.57 to 1640.88) | 7.22 (5.73 to 11.59) | -42.52 (-59.78 to -3.76) |
| Albania | 203.81 (155.74 to 251.09) | 11.72 (8.98 to 14.84) | 143.29 (99.36 to 199.28) | 3.41 (2.36 to 4.72) | -70.89 (-80 to -53.22) |
| Bosnia and Herzegovina | 88.55 (61.84 to 120.48) | 2.75 (1.9 to 3.83) | 56.91 (42.89 to 73.91) | 0.99 (0.75 to 1.29) | -63.81 (-76.15 to -45.69) |
| Bulgaria | 264.15 (232.83 to 296.8) | 2.36 (2.07 to 2.63) | 51.81 (38.36 to 67.7) | 0.37 (0.27 to 0.48) | -84.36 (-88.62 to -79.22) |
| Croatia | 256.1 (228.89 to 283.89) | 4.48 (3.99 to 4.99) | 55.17 (42.17 to 70.33) | 0.59 (0.45 to 0.75) | -86.85 (-89.84 to -83.28) |
| Czechia | 265.46 (242.27 to 306.12) | 2.02 (1.85 to 2.32) | 116.78 (88.98 to 146.22) | 0.59 (0.45 to 0.73) | -71.06 (-77.8 to -62.76) |
| Hungary | 326.47 (301.37 to 374.78) | 2.32 (2.14 to 2.63) | 109.68 (85.35 to 137.41) | 0.58 (0.46 to 0.72) | -74.99 (-80.3 to -68.65) |
| Montenegro | 3.06 (2.42 to 4.19) | 0.53 (0.42 to 0.75) | 3.1 (2.32 to 4.01) | 0.34 (0.25 to 0.44) | -36.76 (-61.65 to -8.75) |
| North Macedonia | 136.9 (110.82 to 166) | 8.85 (6.94 to 10.99) | 57.83 (43.59 to 74.03) | 2.16 (1.64 to 2.75) | -75.57 (-82.71 to -66.32) |
| Poland | 2956.82 (2751.05 to 3108.2) | 7.05 (6.54 to 7.44) | 523.58 (427.94 to 690.63) | 0.73 (0.6 to 0.96) | -89.67 (-91.38 to -86.99) |
| Romania | 1098.48 (738.37 to 1389.6) | 4.41 (2.91 to 5.55) | 254.36 (199.39 to 328.77) | 0.65 (0.51 to 0.84) | -85.26 (-89.6 to -74.16) |
| Serbia | 602.82 (471.11 to 770.2) | 6.04 (4.66 to 7.79) | 331.38 (256.63 to 416.69) | 2.18 (1.7 to 2.7) | -63.99 (-74.73 to -50.21) |
| Slovakia | 102.84 (86.01 to 141.26) | 1.78 (1.5 to 2.4) | 52.03 (39.38 to 70.77) | 0.59 (0.45 to 0.79) | -67 (-74.89 to -56.46) |
| Slovenia | 51.33 (40.01 to 66.32) | 2.18 (1.71 to 2.82) | 19.86 (14.98 to 26.51) | 0.42 (0.32 to 0.57) | -80.66 (-86.74 to -72.52) |
| Belarus | 906.58 (700.4 to 1152.19) | 7.18 (5.59 to 9.14) | 126.83 (79.61 to 287.31) | 0.8 (0.5 to 1.82) | -88.82 (-92.94 to -74.02) |
| Estonia | 81.21 (69.97 to 94.81) | 4.02 (3.49 to 4.68) | 29.59 (22.02 to 40.58) | 1.06 (0.8 to 1.45) | -73.71 (-80.29 to -65.21) |
| Latvia | 168.88 (144.12 to 197.68) | 4.76 (4.06 to 5.54) | 35.27 (26.77 to 53.87) | 0.86 (0.65 to 1.3) | -82.04 (-86.7 to -73.76) |
| Lithuania | 123.15 (101.62 to 146.12) | 2.75 (2.27 to 3.24) | 36.75 (28.17 to 51.91) | 0.63 (0.48 to 0.88) | -77.27 (-83.59 to -65.63) |
| Republic of Moldova | 135.78 (105.11 to 164.07) | 3.21 (2.49 to 3.86) | 30.6 (24.64 to 41.29) | 0.53 (0.43 to 0.73) | -83.43 (-87.66 to -72.4) |
| Russian Federation | 8677.13 (7314.65 to 9434) | 4.91 (4.11 to 5.34) | 1786.37 (1498.89 to 2443.28) | 0.78 (0.66 to 1.07) | -84.03 (-86.68 to -76.32) |
| Ukraine | 1271.18 (714.98 to 1658.15) | 1.97 (1.09 to 2.53) | 280.12 (211.17 to 521.46) | 0.41 (0.31 to 0.74) | -79.28 (-86.58 to -40.89) |
| Australia | 813.45 (722.68 to 881.04) | 4.32 (3.83 to 4.65) | 467.55 (380.46 to 548.97) | 1.14 (0.95 to 1.32) | -73.61 (-77.17 to -69.53) |
| New Zealand | 165.42 (149.69 to 182.23) | 4.4 (3.98 to 4.83) | 77.11 (65.44 to 88.42) | 1.12 (0.98 to 1.26) | -74.62 (-77.62 to -71.3) |
| Brunei Darussalam | 17.74 (13.6 to 21.37) | 30.45 (21.01 to 37.4) | 14.45 (11.92 to 17.45) | 10.94 (8.63 to 13.46) | -64.07 (-72.5 to -50.79) |
| Japan | 6103.33 (5439.67 to 6539.11) | 4.04 (3.59 to 4.32) | 2215.07 (1592.46 to 3000.38) | 0.46 (0.36 to 0.62) | -88.52 (-90.53 to -84.85) |
| Republic of Korea | 6133 (3869.57 to 7153.01) | 35.6 (20.81 to 42.09) | 2673.7 (2001.91 to 3282.02) | 3.48 (2.56 to 4.31) | -90.23 (-92.41 to -83.76) |
| Singapore | 126.62 (104.26 to 138.77) | 5.62 (4.59 to 6.21) | 45.1 (36.94 to 67.19) | 0.62 (0.5 to 0.93) | -89.06 (-91.13 to -81.18) |
| Canada | 510.75 (459.29 to 564.53) | 1.63 (1.47 to 1.8) | 300.28 (231.54 to 358.5) | 0.45 (0.36 to 0.53) | -72.34 (-76.94 to -67.65) |
| Greenland | 2.4 (1.97 to 3.03) | 6.65 (5.32 to 8.75) | 1.21 (0.93 to 1.65) | 1.93 (1.48 to 2.65) | -71 (-78.53 to -60.93) |
| United States of America | 5011.78 (4618.92 to 5803.83) | 1.66 (1.54 to 1.9) | 4071.53 (3353.17 to 4367.45) | 0.87 (0.74 to 0.92) | -47.51 (-57.04 to -44.54) |
| Argentina | 769.26 (653.86 to 896.17) | 2.42 (2.06 to 2.82) | 491.48 (407.07 to 571.67) | 0.92 (0.77 to 1.07) | -61.93 (-69.33 to -53.31) |
| Chile | 260.91 (218.93 to 289.76) | 2.86 (2.37 to 3.18) | 269.75 (209.63 to 332.79) | 1.15 (0.89 to 1.41) | -59.87 (-67 to -50.12) |
| Uruguay | 226.13 (194.47 to 264.99) | 5.97 (5.14 to 6.99) | 122.84 (100.75 to 144.42) | 2.2 (1.83 to 2.55) | -63.12 (-70.61 to -55.56) |
| Andorra | 0.89 (0.64 to 1.28) | 1.84 (1.34 to 2.56) | 1 (0.66 to 1.46) | 0.7 (0.46 to 1.02) | -62.06 (-75.03 to -43.46) |
| Austria | 407.94 (370.73 to 456.09) | 3.39 (3.07 to 3.77) | 78.11 (63.24 to 91.38) | 0.41 (0.34 to 0.48) | -87.78 (-89.79 to -85.69) |
| Belgium | 454.16 (408.37 to 504.58) | 3.12 (2.82 to 3.45) | 149.75 (119.44 to 176.48) | 0.65 (0.54 to 0.75) | -79.19 (-82.18 to -75.7) |
| Cyprus | 43.86 (31.39 to 57.92) | 8.26 (5.58 to 11.33) | 29.41 (21.08 to 43.17) | 1.93 (1.31 to 3.05) | -76.61 (-82.29 to -66.51) |
| Denmark | 228.81 (197.82 to 263.41) | 2.94 (2.57 to 3.33) | 70.36 (56.68 to 83.67) | 0.63 (0.52 to 0.73) | -78.72 (-82.24 to -74.3) |
| Finland | 110.97 (95.41 to 133.71) | 1.63 (1.4 to 1.95) | 109.86 (85.44 to 135.09) | 0.78 (0.62 to 0.94) | -52.17 (-61.58 to -39.86) |
| France | 2289.62 (2029.8 to 2507.71) | 2.8 (2.51 to 3.05) | 1137.77 (898.45 to 1476.65) | 0.73 (0.6 to 0.95) | -73.79 (-77.9 to -66.54) |
| Germany | 6377.59 (5424.49 to 7364.8) | 5.08 (4.34 to 5.8) | 1242.6 (1045.6 to 1468.49) | 0.64 (0.55 to 0.75) | -87.3 (-89.49 to -84.3) |
| Greece | 176.79 (156.92 to 200.44) | 1.24 (1.1 to 1.39) | 66.95 (50.69 to 81.61) | 0.26 (0.21 to 0.31) | -78.93 (-82.68 to -74.71) |
| Iceland | 6.37 (5.6 to 7.33) | 2.2 (1.94 to 2.53) | 2.88 (2.21 to 3.44) | 0.5 (0.39 to 0.59) | -77.22 (-81.88 to -72.44) |
| Ireland | 135.83 (117.18 to 155.3) | 3.46 (3.02 to 3.95) | 50.08 (41.16 to 61.55) | 0.7 (0.58 to 0.86) | -79.64 (-83.17 to -75.13) |
| Israel | 207.15 (177.59 to 239.69) | 4.43 (3.82 to 5.12) | 134.08 (110.36 to 178.28) | 1.11 (0.92 to 1.46) | -75.07 (-79.68 to -66.92) |
| Italy | 1961.9 (1799.2 to 2133.5) | 2.26 (2.06 to 2.46) | 537.44 (428.44 to 608.84) | 0.32 (0.27 to 0.37) | -85.68 (-87.22 to -83.99) |
| Luxembourg | 22.72 (20.09 to 25.74) | 4.35 (3.84 to 4.87) | 10.06 (7.99 to 12.47) | 0.98 (0.78 to 1.21) | -77.41 (-81.7 to -71.74) |
| Malta | 9.39 (8.22 to 10.77) | 2.32 (2.04 to 2.67) | 4.95 (3.87 to 6.17) | 0.53 (0.43 to 0.66) | -76.99 (-81.85 to -70.87) |
| Monaco | 0.57 (0.43 to 0.75) | 0.83 (0.63 to 1.06) | 0.54 (0.41 to 0.67) | 0.55 (0.42 to 0.68) | -34.07 (-51.4 to -9.83) |
| Netherlands | 234.53 (209.8 to 259.18) | 1.21 (1.09 to 1.34) | 115.76 (86.12 to 139.24) | 0.35 (0.27 to 0.41) | -71.52 (-78.17 to -66.23) |
| Norway | 348.59 (320.55 to 399.31) | 4.95 (4.56 to 5.63) | 97.47 (75.9 to 109.8) | 0.92 (0.71 to 1.04) | -81.35 (-86.34 to -78.85) |
| Portugal | 498.98 (442.86 to 553.33) | 3.98 (3.51 to 4.4) | 147.73 (113.37 to 181) | 0.54 (0.43 to 0.66) | -86.44 (-88.87 to -83.31) |
| San Marino | 0.27 (0.21 to 0.34) | 0.86 (0.67 to 1.07) | 0.4 (0.26 to 0.58) | 0.58 (0.37 to 0.85) | -32.95 (-57.95 to 4.85) |
| Spain | 1190.6 (1062.97 to 1324.13) | 2.27 (2.03 to 2.51) | 1122.3 (797.95 to 1444.03) | 0.92 (0.69 to 1.15) | -59.4 (-67.63 to -49.47) |
| Sweden | 383.92 (346.15 to 456.23) | 2.56 (2.33 to 3) | 153.39 (116.2 to 184.09) | 0.65 (0.51 to 0.77) | -74.47 (-81.29 to -69.98) |
| Switzerland | 280.71 (234.95 to 324.19) | 2.65 (2.23 to 3.05) | 109.91 (85.8 to 132.92) | 0.54 (0.43 to 0.64) | -79.59 (-83.64 to -75.07) |
| United Kingdom | 2170.8 (2037.21 to 2593.4) | 2.67 (2.51 to 3.16) | 1523.54 (1283.97 to 1713.91) | 1.22 (1.06 to 1.36) | -54.19 (-60.74 to -49.15) |
| Bolivia (Plurinational State of) | 431.13 (264.6 to 637.86) | 8.49 (5.65 to 11.41) | 198.27 (146.46 to 261.02) | 2.43 (1.77 to 3.2) | -71.4 (-79.19 to -59.97) |
| Ecuador | 338.84 (191.88 to 436.82) | 4.21 (2.85 to 5.42) | 94.19 (74.14 to 124.82) | 0.7 (0.55 to 0.92) | -83.39 (-88.75 to -73.57) |
| Peru | 521.3 (369.67 to 690.44) | 2.94 (2.2 to 3.64) | 228.85 (159.44 to 313.48) | 0.7 (0.49 to 0.97) | -76.16 (-84.49 to -63.23) |
| Antigua and Barbuda | 1.61 (1.39 to 1.88) | 2.9 (2.52 to 3.37) | 1.84 (1.43 to 2.28) | 1.95 (1.53 to 2.39) | -32.77 (-47.67 to -14.54) |
| Bahamas | 6.12 (5.28 to 7.16) | 3.68 (3.14 to 4.31) | 7.89 (6.13 to 10.15) | 2.06 (1.61 to 2.62) | -43.83 (-57.46 to -27.39) |
| Barbados | 12.72 (10.74 to 14.69) | 4.36 (3.7 to 5.02) | 13.14 (10.31 to 16.3) | 2.84 (2.23 to 3.5) | -34.9 (-50.83 to -17.61) |
| Belize | 4.49 (3.73 to 5.35) | 3.59 (2.92 to 4.48) | 8.44 (6.9 to 10.18) | 2.7 (2.21 to 3.27) | -24.64 (-44.83 to 2.78) |
| Bermuda | 1.96 (1.48 to 2.42) | 3.3 (2.51 to 4.04) | 1.09 (0.85 to 1.38) | 0.88 (0.7 to 1.1) | -73.43 (-80.19 to -63.51) |
| Cuba | 260.24 (218.19 to 288.43) | 2.45 (2.05 to 2.71) | 261.02 (193.84 to 332.28) | 1.52 (1.16 to 1.93) | -37.87 (-51.08 to -19.55) |
| Dominica | 4.55 (3.79 to 5.48) | 6.34 (5.32 to 7.66) | 4.36 (3.29 to 5.6) | 5.05 (3.8 to 6.49) | -20.23 (-44.72 to 6.21) |
| Dominican Republic | 486.07 (375.5 to 568.57) | 10.6 (8.21 to 12.13) | 477.16 (345.71 to 632.77) | 5.17 (3.75 to 6.84) | -51.25 (-65.29 to -33.28) |
| Grenada | 3.4 (2.82 to 4.12) | 4.52 (3.76 to 5.42) | 2.13 (1.73 to 2.52) | 2.03 (1.65 to 2.4) | -55.03 (-65.85 to -42.36) |
| Guyana | 35.89 (30.52 to 42.39) | 8.55 (7.21 to 10.14) | 29.18 (21.7 to 38.18) | 4.55 (3.43 to 5.94) | -46.83 (-61.74 to -28.62) |
| Haiti | 1155.92 (747.9 to 1640.93) | 26.29 (15.46 to 47.01) | 1093.83 (674.09 to 1665.2) | 13.08 (7.57 to 22.31) | -50.26 (-64.25 to -30.74) |
| Jamaica | 75.65 (65.93 to 87.72) | 3.86 (3.35 to 4.55) | 76.98 (58.35 to 100.62) | 2.54 (1.92 to 3.32) | -34.22 (-52.54 to -12.33) |
| Puerto Rico | 162.18 (144.31 to 185.69) | 4.6 (4.11 to 5.26) | 93.49 (70.04 to 120.27) | 1.44 (1.09 to 1.87) | -68.75 (-76.59 to -59.14) |
| Saint Kitts and Nevis | 1.82 (1.54 to 2.12) | 5.04 (4.28 to 5.89) | 1.17 (0.9 to 1.49) | 1.94 (1.51 to 2.43) | -61.6 (-70.61 to -49.31) |
| Saint Lucia | 7.98 (6.94 to 9.06) | 8.79 (7.65 to 10.06) | 8.45 (6.49 to 10.49) | 4.15 (3.21 to 5.14) | -52.78 (-64.6 to -40.5) |
| Saint Vincent and the Grenadines | 3.56 (3.08 to 4.14) | 4.71 (4.02 to 5.48) | 3.85 (3.08 to 4.69) | 3.02 (2.42 to 3.65) | -35.81 (-52.64 to -20.14) |
| Suriname | 13.08 (11.08 to 15.19) | 4.68 (4.02 to 5.42) | 14.72 (11.53 to 18.26) | 2.52 (1.98 to 3.12) | -46.14 (-58.26 to -30.26) |
| Trinidad and Tobago | 73.71 (66.43 to 80.9) | 8.64 (7.79 to 9.6) | 54.78 (39.61 to 75.24) | 3.25 (2.35 to 4.4) | -62.4 (-73.18 to -48.96) |
| United States Virgin Islands | 3.66 (3.03 to 4.41) | 4.44 (3.67 to 5.31) | 4.3 (3.37 to 5.31) | 2.54 (1.99 to 3.11) | -42.87 (-57.83 to -25.83) |
| Colombia | 759.1 (661.39 to 822.21) | 4.21 (3.59 to 4.57) | 263.8 (192.39 to 346.99) | 0.5 (0.36 to 0.66) | -88.15 (-91.32 to -84.04) |
| Costa Rica | 45.4 (39.69 to 52.86) | 2.33 (2.01 to 2.74) | 50.76 (37.13 to 67.18) | 0.99 (0.72 to 1.3) | -57.63 (-69.82 to -42.26) |
| El Salvador | 350.52 (252.7 to 422.82) | 9.86 (7.52 to 12.09) | 174.19 (123.88 to 229.81) | 2.75 (1.95 to 3.64) | -72.15 (-82.06 to -59.77) |
| Guatemala | 732.34 (531.59 to 857.33) | 14.35 (11.18 to 17.53) | 326.96 (247.27 to 437.69) | 2.97 (2.27 to 3.95) | -79.29 (-85.95 to -69.27) |
| Honduras | 383.08 (265.91 to 497.34) | 11.82 (8.62 to 15.57) | 371.65 (240.41 to 527.3) | 6.19 (4 to 8.71) | -47.64 (-66.79 to -24.72) |
| Mexico | 2871.28 (2520.8 to 3052.31) | 7.36 (6.36 to 7.84) | 1655.17 (1341.59 to 1930.88) | 1.52 (1.23 to 1.77) | -79.34 (-83.18 to -75.92) |
| Nicaragua | 149.03 (122.38 to 178.77) | 7.09 (5.94 to 8.34) | 100.71 (77.39 to 137.87) | 2.59 (1.98 to 3.5) | -63.55 (-73.61 to -46.18) |
| Panama | 64.03 (53.47 to 73.36) | 3.57 (3.05 to 4.11) | 60.55 (44.47 to 78.29) | 1.45 (1.06 to 1.87) | -59.54 (-70.96 to -44.33) |
| Venezuela (Bolivarian Republic of) | 392.5 (333.92 to 443.25) | 3.27 (2.81 to 3.77) | 415.26 (305.27 to 554.2) | 1.47 (1.08 to 1.96) | -55.11 (-68.14 to -38.07) |
| Brazil | 3225.89 (2837.73 to 3524.34) | 3.48 (3.04 to 3.83) | 2849.42 (2542.19 to 3359.9) | 1.27 (1.13 to 1.5) | -63.39 (-67.88 to -56.51) |
| Paraguay | 58.47 (49.07 to 70.22) | 2.41 (2.01 to 2.97) | 90.1 (64.3 to 118.6) | 1.67 (1.19 to 2.19) | -30.85 (-53.62 to -3.92) |
| Afghanistan | 2967.63 (1906.05 to 4265.66) | 45.35 (27.95 to 67.56) | 2947.18 (1953.88 to 4134.08) | 25.78 (15.93 to 36.64) | -43.15 (-57.9 to -25.58) |
| Algeria | 2122.06 (1569.28 to 2804.94) | 23.32 (17.09 to 30.94) | 2249.54 (1728.19 to 2848.49) | 8.53 (6.5 to 10.73) | -63.41 (-72.92 to -50.07) |
| Bahrain | 23.3 (19.48 to 28.67) | 14.96 (12.21 to 19.22) | 31.11 (23.81 to 41.79) | 4.87 (3.81 to 6.53) | -67.47 (-76.24 to -56.9) |
| Egypt | 7950.28 (6153.99 to 9827.4) | 28.54 (22.47 to 36.04) | 7489.93 (5033.26 to 10490.67) | 13.66 (9.43 to 18.75) | -52.13 (-66.19 to -36.18) |
| Iran (Islamic Republic of) | 3806.11 (3075.15 to 4777.05) | 18.52 (14 to 24.95) | 3605.61 (3095.35 to 4029.75) | 5.61 (4.77 to 6.25) | -69.71 (-78.87 to -59.7) |
| Iraq | 1166.38 (903.73 to 1468.55) | 14.93 (11.56 to 19.32) | 1157.34 (891.31 to 1474.27) | 5.71 (4.5 to 7.32) | -61.78 (-72.35 to -47.24) |
| Jordan | 113.03 (90.28 to 137.14) | 9.28 (7.24 to 11.65) | 142.99 (115.64 to 178.41) | 2.61 (2.09 to 3.28) | -71.88 (-79.61 to -61.63) |
| Kuwait | 33.24 (27.9 to 37.78) | 5.57 (4.5 to 6.62) | 32.93 (26.27 to 41.62) | 1.46 (1.14 to 1.85) | -73.83 (-79.78 to -65.18) |
| Lebanon | 292.49 (217.13 to 409.44) | 15.24 (11.15 to 21.34) | 278.79 (177.05 to 376.37) | 5.45 (3.47 to 7.37) | -64.23 (-78.89 to -48.44) |
| Libya | 252.99 (176.97 to 358.6) | 14.4 (9.77 to 21.02) | 350.13 (241.26 to 471.06) | 7.67 (5.33 to 10.4) | -46.75 (-63.44 to -20.46) |
| Morocco | 2515.26 (1828.2 to 3698.07) | 21.05 (14.89 to 33.03) | 3053.95 (2254.84 to 4072.69) | 11.76 (8.62 to 15.83) | -44.12 (-57.96 to -29.06) |
| Oman | 10.75 (7.42 to 16.95) | 1.87 (1.24 to 3.09) | 6.99 (5.6 to 9.07) | 0.64 (0.48 to 0.84) | -65.57 (-81.25 to -43.61) |
| Palestine | 106.92 (76.79 to 140.15) | 13.09 (9.35 to 17.52) | 98.89 (76.3 to 120.38) | 5.21 (3.93 to 6.33) | -60.2 (-72.36 to -43.61) |
| Qatar | 5.09 (3.74 to 7.48) | 4.66 (3.06 to 8.7) | 8.26 (5.96 to 12.18) | 1.36 (1.02 to 1.94) | -70.8 (-84.94 to -51.05) |
| Saudi Arabia | 853.11 (602.7 to 1236.43) | 15.94 (11.16 to 23.98) | 906.22 (684.12 to 1225.22) | 6.15 (4.73 to 8.03) | -61.45 (-77.98 to -38.52) |
| Sudan | 3195.65 (1949.03 to 4946.29) | 34.68 (19.7 to 60.74) | 2483.98 (1551.91 to 3790.51) | 14.72 (8.97 to 23.46) | -57.54 (-66.93 to -41.19) |
| Syrian Arab Republic | 1167.54 (908.88 to 1479.67) | 22.36 (16.66 to 29.66) | 1171.84 (873.94 to 1539.1) | 12.38 (9.29 to 15.95) | -44.64 (-60.98 to -20.59) |
| Tunisia | 612.41 (474.99 to 838.64) | 14.25 (10.84 to 19.59) | 686.84 (485.38 to 917) | 6.11 (4.32 to 8.18) | -57.13 (-72.18 to -39.16) |
| Turkey | 3309.08 (2564.53 to 4595.64) | 9.83 (7.37 to 14.24) | 2666.19 (1981.85 to 3587.67) | 3.32 (2.46 to 4.5) | -66.28 (-78.05 to -50.59) |
| United Arab Emirates | 155.34 (96.45 to 250.99) | 33.65 (22.32 to 52.87) | 550.09 (312.57 to 1011.53) | 12.24 (7.58 to 21.41) | -63.63 (-75.91 to -45.76) |
| Yemen | 1738.7 (1114.7 to 2638.17) | 38.29 (23.34 to 64.6) | 2124.65 (1546.72 to 3038.78) | 18.04 (13.12 to 26.32) | -52.88 (-68.55 to -27.83) |
| Bangladesh | 15262.32 (10818.76 to 22340.28) | 32.58 (22.28 to 49.86) | 10719.29 (7475.33 to 17539.04) | 8.83 (6.16 to 14.55) | -72.89 (-82.08 to -57.14) |
| Bhutan | 74.7 (43.32 to 129.28) | 34.27 (19.55 to 64.26) | 75.28 (52.48 to 112.69) | 15.23 (10.73 to 22.81) | -55.55 (-72.2 to -28.88) |
| India | 149454.5 (90390.89 to 227979.18) | 41.98 (23.9 to 67.5) | 198798.87 (129621.75 to 271915.86) | 20.05 (12.9 to 28.19) | -52.24 (-63.32 to -40.53) |
| Nepal | 5145.44 (3022.18 to 7571.67) | 63.26 (35.89 to 101.19) | 6900.81 (4301.41 to 9792.62) | 36.4 (22.45 to 52.15) | -42.46 (-60.58 to -19.31) |
| Pakistan | 15177.62 (10100.61 to 24482.66) | 28.09 (18.24 to 47.46) | 15693.77 (12142.76 to 21425.53) | 16.39 (12.59 to 22.93) | -41.65 (-57.05 to -19.41) |
| China | 40414.03 (30325.5 to 58426.14) | 6.37 (4.69 to 9.45) | 24750.22 (20244.86 to 30769.13) | 1.51 (1.23 to 1.86) | -76.35 (-85.85 to -63.78) |
| Democratic People's Republic of Korea | 1294.2 (858.41 to 1867.65) | 10.24 (6.58 to 15.41) | 1635.63 (1073.02 to 2524.26) | 5.69 (3.66 to 8.99) | -44.47 (-60.78 to -20.78) |
| Taiwan (Province of China) | 1785.19 (1561.54 to 1928.09) | 14.5 (12.57 to 15.84) | 825.24 (618.63 to 1103.99) | 2.06 (1.54 to 2.76) | -85.78 (-89.42 to -80.55) |
| American Samoa | 3.83 (3.15 to 4.7) | 21.82 (17.74 to 26.89) | 4.25 (3.39 to 5.36) | 11 (8.64 to 13.95) | -49.56 (-63.6 to -32.42) |
| Cook Islands | 1.64 (1.24 to 2.08) | 15.65 (11.64 to 19.99) | 1.35 (0.92 to 1.86) | 6.02 (4.11 to 8.35) | -61.52 (-72.06 to -48.4) |
| Fiji | 268.48 (214.36 to 335.43) | 95.03 (76.38 to 119.1) | 242.91 (187.96 to 314.85) | 43.33 (34.22 to 54.76) | -54.4 (-68.32 to -36.2) |
| Guam | 4.58 (3.74 to 5.39) | 9.87 (7.79 to 11.89) | 7.47 (5.75 to 9.51) | 4.03 (3.1 to 5.18) | -59.2 (-69.42 to -45.97) |
| Kiribati | 32.65 (26.61 to 40.02) | 105.34 (84.79 to 140.79) | 39.61 (30.24 to 50.6) | 80.5 (59.22 to 104.19) | -23.59 (-44.77 to -1.94) |
| Marshall Islands | 6.83 (5 to 10.01) | 47.22 (34.45 to 71.35) | 7.14 (4.67 to 9.98) | 27.2 (18.48 to 37.53) | -42.4 (-58.8 to -23.79) |
| Micronesia (Federated States of) | 21.26 (15.71 to 29.43) | 53.47 (38.58 to 80.2) | 14.25 (9.98 to 19.79) | 27.69 (20.23 to 37.7) | -48.22 (-69.2 to -24.16) |
| Nauru | 1.25 (0.87 to 1.6) | 36.89 (27.7 to 46.32) | 0.7 (0.4 to 0.97) | 21.13 (13.06 to 28.16) | -42.7 (-62.18 to -21.96) |
| Niue | 0.57 (0.39 to 0.73) | 23.89 (16.67 to 30.47) | 0.23 (0.13 to 0.32) | 11.04 (6.24 to 15.55) | -53.8 (-70.67 to -35.65) |
| Northern Mariana Islands | 1.8 (1.41 to 2.27) | 13.73 (10.91 to 17.03) | 2.15 (1.74 to 2.75) | 6.65 (5.24 to 8.47) | -51.56 (-65.38 to -34.44) |
| Palau | 1.82 (1.4 to 2.4) | 22.64 (17.22 to 30.22) | 1.93 (1.5 to 2.43) | 12.81 (10 to 15.92) | -43.43 (-59.83 to -21.72) |
| Papua New Guinea | 1037.6 (767.33 to 1452.84) | 72.76 (51.51 to 107.82) | 1939.75 (1370.16 to 2752.88) | 55.81 (39.05 to 80.31) | -23.31 (-42.19 to -1.23) |
| Samoa | 29.4 (21.39 to 42.59) | 39.02 (28.38 to 57.65) | 25.42 (19.49 to 32.68) | 19.86 (15.35 to 25.5) | -49.09 (-67.66 to -25.32) |
| Solomon Islands | 57.02 (40.84 to 76.42) | 49.12 (34.66 to 69.36) | 91.33 (64.76 to 118.86) | 36.46 (25.23 to 46.75) | -25.78 (-46.78 to 0.38) |
| Tokelau | 0.45 (0.34 to 0.61) | 35.61 (26.94 to 47.94) | 0.17 (0.12 to 0.22) | 14.23 (10.29 to 18.75) | -60.05 (-73.79 to -43.23) |
| Tonga | 9.1 (7.19 to 12.21) | 19.84 (15.41 to 26.82) | 9.11 (6.81 to 11.62) | 11.9 (8.93 to 15.17) | -40.02 (-58.56 to -18.13) |
| Tuvalu | 2.9 (2.09 to 4.04) | 52.16 (36.08 to 76.39) | 1.79 (1.29 to 2.41) | 21.37 (15.23 to 28.49) | -59.03 (-75.04 to -37.37) |
| Vanuatu | 26.54 (16.77 to 41.78) | 47.53 (30.15 to 79.43) | 45.37 (31.37 to 66.35) | 31.55 (21.84 to 47.19) | -33.61 (-49.94 to -10.19) |
| Cambodia | 1136.16 (851.52 to 1371.18) | 26.22 (18.47 to 34.26) | 1359.33 (1038.87 to 1674.89) | 13.67 (10.42 to 16.71) | -47.87 (-62.78 to -31.88) |
| Indonesia | 24274.05 (19535.98 to 30694.63) | 26.58 (21.16 to 35.81) | 26457.66 (21282.11 to 32819.96) | 14.87 (11.99 to 18.55) | -44.05 (-57.74 to -27.68) |
| Lao People's Democratic Republic | 1185.14 (864.97 to 1490.78) | 53.4 (38.29 to 71.76) | 820.92 (590.79 to 1126.27) | 21.11 (15.24 to 29.06) | -60.48 (-71.24 to -45.61) |
| Malaysia | 1509.93 (1219.97 to 1809.35) | 16.75 (13.5 to 20.54) | 938.72 (680.79 to 1411.6) | 3.83 (2.77 to 5.79) | -77.15 (-85.64 to -63.13) |
| Maldives | 19.77 (12.68 to 27.28) | 23.37 (15.56 to 33.94) | 10.39 (8.3 to 12.94) | 4.2 (3.31 to 5.24) | -82.01 (-88.96 to -73.21) |
| Mauritius | 243.24 (207.53 to 265.78) | 38.43 (32.58 to 42.19) | 146.61 (112.57 to 196.7) | 9.55 (7.38 to 12.72) | -75.15 (-81.18 to -64.76) |
| Myanmar | 16004.48 (10751.65 to 20422.83) | 74.11 (47.22 to 98.38) | 11043.03 (7905.19 to 13819.9) | 27.8 (19.51 to 34.4) | -62.48 (-71.92 to -50.08) |
| Philippines | 9191.07 (8097.14 to 10255.52) | 32.01 (27.71 to 36.72) | 11776.38 (9712.04 to 14703.12) | 16.36 (13.54 to 21.18) | -48.89 (-59.55 to -31.42) |
| Seychelles | 7.38 (5.82 to 9.57) | 13 (10.2 to 16.77) | 6.37 (4.38 to 8.48) | 6.59 (4.46 to 8.95) | -49.26 (-62.43 to -36.45) |
| Sri Lanka | 6682.49 (5604.74 to 7565.14) | 80.95 (67.77 to 91.19) | 8228.72 (5744.13 to 10722.7) | 40.31 (27.9 to 51.84) | -50.2 (-64.99 to -34.2) |
| Thailand | 4630.67 (3346.3 to 5833.8) | 15.02 (10.5 to 19.31) | 4193.72 (3053.05 to 5581.75) | 4.41 (3.21 to 5.83) | -70.66 (-80.71 to -54.98) |
| Timor-Leste | 120.1 (81.09 to 159.76) | 36.83 (25.08 to 53.61) | 144.63 (97.95 to 203) | 19.75 (13.47 to 27.95) | -46.37 (-61.36 to -24.02) |
| Viet Nam | 7609.93 (2721.72 to 10854.36) | 20.56 (7.09 to 30.2) | 6834.5 (2814.45 to 9199.92) | 8.68 (3.55 to 11.87) | -57.8 (-78.16 to -36.11) |
| Angola | 1356.2 (953.43 to 1880.96) | 32.12 (20.81 to 54.91) | 1484.62 (1047.82 to 2099.67) | 14.54 (10.25 to 23.21) | -54.72 (-67.13 to -36.16) |
| Central African Republic | 499.63 (326.93 to 724.06) | 43.94 (26.1 to 77.67) | 709.74 (428.53 to 1224.66) | 35.4 (19.28 to 74.16) | -19.44 (-42.86 to 10.31) |
| Congo | 324.85 (221.12 to 500.86) | 32.69 (20.97 to 58.25) | 325.5 (216.35 to 463.4) | 14.57 (9.84 to 22.4) | -55.44 (-68.35 to -36.7) |
| Democratic Republic of the Congo | 4735.76 (2913.46 to 7208.22) | 30.26 (17.09 to 55.72) | 7140.8 (4144.43 to 13524.78) | 22.48 (11.83 to 48.44) | -25.72 (-48.04 to 1.22) |
| Equatorial Guinea | 72.98 (45.18 to 117.15) | 38.01 (21.65 to 72.17) | 44.73 (26.1 to 82.69) | 10.51 (6.07 to 20.36) | -72.35 (-83.99 to -53.49) |
| Gabon | 110.03 (80.22 to 150.35) | 21.21 (15.16 to 31.27) | 86.89 (62.25 to 116.42) | 9.55 (6.88 to 12.86) | -54.96 (-70.12 to -32.76) |
| Burundi | 919.64 (593.33 to 1238.68) | 31.09 (20.06 to 44.58) | 815.25 (542.18 to 1325.57) | 16.28 (10.44 to 28.78) | -47.62 (-63.53 to -25.55) |
| Comoros | 49.68 (25.41 to 71.87) | 20.89 (12.06 to 30.22) | 50.32 (36.29 to 70.15) | 10.64 (7.77 to 15.19) | -49.07 (-63.46 to -19.1) |
| Djibouti | 29.01 (19.15 to 40.71) | 17.09 (11.05 to 23.43) | 50.17 (29.4 to 72.1) | 8.89 (4.76 to 12.31) | -47.98 (-63.98 to -24.15) |
| Eritrea | 362.55 (224.44 to 482.35) | 28.31 (18.11 to 41.66) | 422.17 (281.96 to 599.37) | 15.18 (9.86 to 21.64) | -46.37 (-62.97 to -22.06) |
| Ethiopia | 5394.31 (3643.95 to 6747.07) | 22.71 (15.51 to 31.92) | 3698.85 (2744.12 to 5827.21) | 8.54 (6.21 to 14.61) | -62.41 (-71.67 to -46.2) |
| Kenya | 1194.83 (808.23 to 1925.95) | 13.3 (8.54 to 23.7) | 2171.27 (1448.13 to 3749) | 10.17 (6.69 to 18.61) | -23.58 (-38.12 to -4.39) |
| Madagascar | 1949.48 (1562.92 to 2343.42) | 27.99 (21.82 to 37.2) | 2360.71 (1642.05 to 3240.98) | 19.29 (13.32 to 26.64) | -31.09 (-48.97 to -8.24) |
| Malawi | 799.28 (557.23 to 1042.67) | 17.33 (12.31 to 22.62) | 817.8 (585.85 to 1088.85) | 10.8 (7.51 to 15.25) | -37.68 (-52.26 to -15.09) |
| Mozambique | 1124.33 (764.81 to 1444.21) | 16.45 (11.7 to 21.64) | 1388.82 (1004.17 to 1835.96) | 11.92 (8.36 to 16.19) | -27.55 (-48.29 to -1.64) |
| Rwanda | 1153.9 (764.77 to 1539.27) | 32.56 (21.52 to 46.75) | 760.01 (516.35 to 1217.41) | 12.58 (8.5 to 21.48) | -61.36 (-72.41 to -42.19) |
| Somalia | 1049.3 (595.27 to 1501.74) | 34.1 (18.94 to 52.93) | 1814.9 (1079.59 to 3081.14) | 24.56 (13.93 to 45.57) | -27.99 (-47.99 to -0.4) |
| South Sudan | 602.22 (385.59 to 807.39) | 20.41 (12.53 to 28.53) | 500.74 (327.14 to 775.09) | 12.48 (7.82 to 20.84) | -38.84 (-57.9 to -16.24) |
| Uganda | 1628.97 (976.44 to 2377.04) | 21.29 (12.82 to 33.99) | 1802.17 (1235.89 to 2673.97) | 11.68 (8.03 to 18.76) | -45.14 (-60.4 to -23.02) |
| United Republic of Tanzania | 1662.67 (1269.05 to 2094.93) | 13.42 (9.94 to 17.93) | 2315.59 (1683.73 to 3132.38) | 8.61 (6.11 to 12.3) | -35.8 (-50.41 to -16.39) |
| Zambia | 694.35 (521.75 to 876.22) | 19.5 (14.74 to 25.51) | 780 (594.61 to 1011.12) | 10.87 (8.12 to 14.18) | -44.27 (-58.77 to -26.07) |
| Botswana | 185.35 (129.25 to 258.33) | 34.09 (23.8 to 48.04) | 202.25 (128.53 to 287.02) | 16 (9.95 to 22.76) | -53.06 (-73.37 to -29.62) |
| Eswatini | 101.68 (73.61 to 139.47) | 34.58 (24.6 to 48.53) | 108.23 (77.97 to 146.3) | 20.02 (14.78 to 26.49) | -42.11 (-60.77 to -15.09) |
| Lesotho | 340.62 (214.61 to 544.11) | 36.09 (22.52 to 58.36) | 355.68 (231.81 to 525.75) | 29.85 (19.61 to 46.08) | -17.3 (-45.59 to 27.92) |
| Namibia | 239.6 (168.68 to 347.32) | 35.19 (24.91 to 51.78) | 209.63 (155.93 to 281.09) | 15.94 (11.86 to 21.26) | -54.7 (-69.01 to -33.85) |
| South Africa | 4566.8 (3936.22 to 5452.3) | 20.75 (17.62 to 26.03) | 4668.73 (4215.08 to 5348.31) | 11.61 (10.37 to 13.16) | -44.07 (-55.12 to -34.62) |
| Zimbabwe | 959.16 (795.39 to 1138.98) | 24.97 (20.57 to 30.86) | 1564.86 (1053.75 to 2159.04) | 23.65 (15.65 to 33.15) | -5.28 (-35.43 to 28.21) |
| Benin | 609.03 (468.2 to 767.72) | 29.09 (22.15 to 37.16) | 731.04 (540.78 to 985.5) | 14.83 (11.28 to 19.63) | -49.02 (-61.38 to -32.04) |
| Burkina Faso | 789.36 (605 to 976.87) | 17.82 (13.66 to 22.3) | 1108.21 (841.52 to 1425.28) | 11.42 (8.93 to 14.71) | -35.91 (-50.01 to -17.42) |
| Cabo Verde | 42.21 (25.09 to 52.42) | 17.64 (10.45 to 21.77) | 27.26 (19.36 to 37.99) | 6.34 (4.49 to 8.88) | -64.05 (-78.38 to -22.59) |
| Cameroon | 1182.04 (941.22 to 1441.42) | 27.63 (21.89 to 33.8) | 1505.77 (1096.44 to 2033.16) | 13.07 (9.92 to 17.2) | -52.71 (-66.54 to -32.45) |
| Chad | 889.91 (641.86 to 1175.69) | 31.13 (22.2 to 42.84) | 1156.01 (875.88 to 1504.55) | 19.69 (14.94 to 25.84) | -36.76 (-51.58 to -14.36) |
| Côte d'Ivoire | 1095.59 (830.47 to 1366.3) | 27.34 (20.67 to 33.81) | 1409.59 (1054.05 to 1868.71) | 13.73 (10.78 to 17.63) | -49.79 (-62.26 to -32.76) |
| Gambia | 89.78 (63.53 to 119.92) | 25.97 (18.73 to 34.23) | 153.13 (112.35 to 205.43) | 16.48 (12.06 to 22.24) | -36.55 (-55.31 to -9.11) |
| Ghana | 1483.67 (941.14 to 1838.33) | 24.02 (15.91 to 29.95) | 2120.2 (1404.5 to 2749.51) | 13.97 (9.32 to 17.77) | -41.84 (-57.65 to -22.82) |
| Guinea | 1067.95 (802.9 to 1362.86) | 31.92 (24.07 to 42.67) | 1136.05 (839.97 to 1470.98) | 19.9 (14.76 to 26.13) | -37.64 (-53.45 to -16.96) |
| Guinea-Bissau | 182.43 (137.69 to 230.85) | 43.23 (32.99 to 53.56) | 162.44 (120.7 to 210.4) | 22.44 (16.74 to 28.95) | -48.09 (-62.86 to -28.59) |
| Liberia | 205.62 (161.07 to 257.42) | 17.84 (14.16 to 22.57) | 200.21 (138.73 to 283.73) | 10.08 (7.14 to 14.08) | -43.53 (-58.74 to -21.11) |
| Mali | 1405.63 (977.28 to 1747.95) | 33.92 (23.45 to 42.63) | 1793.88 (1240.31 to 2472.86) | 20.15 (14.01 to 26.86) | -40.59 (-54.85 to -23) |
| Mauritania | 237.04 (164.11 to 300.07) | 24.75 (17.04 to 31.45) | 174.16 (123.79 to 239.27) | 8.93 (6.4 to 11.96) | -63.93 (-73.8 to -48.03) |
| Niger | 1034.35 (740.25 to 1319.15) | 34.08 (24.1 to 46.73) | 1555.67 (1058.8 to 2240.66) | 19.47 (13.42 to 28.3) | -42.86 (-57.01 to -23.09) |
| Nigeria | 7818.23 (5952.06 to 9992.4) | 18.6 (13.95 to 24.21) | 8746.46 (6848.75 to 11134.7) | 10.72 (8.46 to 13.61) | -42.35 (-56.36 to -23.35) |
| Sao Tome and Principe | 25 (19.56 to 30.49) | 41.06 (32.35 to 49.68) | 25.07 (17.39 to 32.86) | 26.13 (17.66 to 34.16) | -36.35 (-56.16 to -12.77) |
| Senegal | 896.68 (657.12 to 1128.04) | 27.49 (20.28 to 35) | 1010.41 (772.17 to 1315.62) | 14.22 (10.97 to 18.62) | -48.27 (-61.5 to -28.26) |
| Sierra Leone | 532.95 (381.83 to 682.52) | 27.31 (19.58 to 35.23) | 574.28 (414.95 to 770.72) | 15.67 (11.56 to 20.77) | -42.61 (-57.47 to -20.97) |
| Togo | 358.21 (275.45 to 453.04) | 28.28 (21.29 to 35.72) | 517.42 (372.97 to 702.98) | 15.31 (11.26 to 20.82) | -45.87 (-60.98 to -26.47) |

**Table S4.** Disability adjusted life years (DALYs) of asthma in 1990 and 2019 and the percentage change in the age-standardized rates (ASRs) per 100,000, by location

|  | 1990 | | 2019 | | Percentage change in ASRs per 100,000 |
| --- | --- | --- | --- | --- | --- |
| countries and territories | No (95% UI) | ASRs per 100 000 (95% UI) | No (95% UI) | ASRs per 100 000 (95% UI) |  |
| Armenia | 3931.16 (2868.21 to 5358.63) | 127.15 (94.55 to 167.83) | 2782.38 (1850.05 to 3988.34) | 91.09 (59.15 to 136.04) | -28.35 (-43.63 to -14.52) |
| Azerbaijan | 16182.92 (12997.86 to 20076.38) | 285.33 (231.56 to 352.08) | 15681.32 (11998.15 to 21013.47) | 172.37 (131.65 to 235.99) | -39.59 (-54.75 to -19.62) |
| Georgia | 13772 (11500.57 to 16262.36) | 236.04 (196.96 to 281.59) | 5102.48 (3991.04 to 6522.09) | 125.32 (95.62 to 167.92) | -46.91 (-55.57 to -36.82) |
| Kazakhstan | 40586.73 (32385.23 to 50664.73) | 301.61 (238.79 to 378.23) | 37622.77 (29570.97 to 46982.29) | 221.01 (173.52 to 275.57) | -26.72 (-41 to -9.41) |
| Kyrgyzstan | 9753.71 (7537.52 to 12453.09) | 263.48 (207.6 to 330.59) | 7744.47 (5436.23 to 11033.54) | 126.35 (91.46 to 175.84) | -52.05 (-60.79 to -41.01) |
| Mongolia | 5445.01 (4275.42 to 6822.26) | 426.48 (337.6 to 534.85) | 5112.41 (3851.06 to 6786.67) | 197.24 (152.5 to 257.36) | -53.75 (-64.78 to -35.81) |
| Tajikistan | 11604.14 (9014.74 to 15150.84) | 347.14 (265.7 to 450.81) | 12762.3 (9476.45 to 17027.91) | 224.27 (175.23 to 279.02) | -35.4 (-51.43 to -17.46) |
| Turkmenistan | 10615.44 (8150.67 to 13172.29) | 384.23 (294.25 to 473.39) | 5536.26 (3855.4 to 7773.96) | 113.65 (78.77 to 159.23) | -70.42 (-78.31 to -56.13) |
| Uzbekistan | 62921.98 (48490.49 to 79004.56) | 446.94 (347.32 to 553.2) | 68396.13 (52527 to 88502.42) | 265.51 (209.66 to 351.74) | -40.59 (-52.66 to -22.36) |
| Albania | 7186.24 (5865.68 to 8890.97) | 301.94 (248.16 to 364.12) | 5376.18 (3990.03 to 6982.38) | 165.88 (120.96 to 222.78) | -45.06 (-57.05 to -30.17) |
| Bosnia and Herzegovina | 9672.76 (6654.41 to 13670.47) | 226.53 (158.65 to 317.3) | 7074.34 (4809.23 to 9973.71) | 189.79 (125.73 to 278.98) | -16.22 (-26.36 to -7.43) |
| Bulgaria | 21140.74 (15574.5 to 28268.45) | 213.74 (153.77 to 294.39) | 11038.58 (7404.34 to 15928.09) | 149.87 (96.64 to 220.62) | -29.88 (-38.82 to -21.48) |
| Croatia | 15592.41 (11554.23 to 20821.68) | 282.08 (205.99 to 382.42) | 7980.75 (5381.39 to 11339.07) | 166.32 (109.02 to 248.94) | -41.04 (-49.44 to -33.82) |
| Czechia | 19864.1 (15126.52 to 26233.14) | 179.79 (134.39 to 243.02) | 14940.76 (10280.79 to 20862.08) | 133.94 (88.49 to 197.11) | -25.5 (-35.38 to -17.36) |
| Hungary | 25450.6 (19410.28 to 33619.49) | 218.87 (162.22 to 296.71) | 14626.55 (10149.06 to 20478.79) | 143.64 (95.95 to 212.33) | -34.38 (-43.29 to -26.65) |
| Montenegro | 868.99 (568.84 to 1280.4) | 139.08 (91.45 to 205.41) | 946.32 (616.34 to 1392.79) | 148.37 (94.72 to 223.34) | 6.68 (-1.26 to 15.94) |
| North Macedonia | 7807.16 (5691.09 to 10565.11) | 417.89 (306.01 to 561.18) | 5715.78 (4058.85 to 7895.55) | 234.59 (163.12 to 335.18) | -43.86 (-53.14 to -33.21) |
| Poland | 213272.31 (158931.15 to 285072.01) | 516.38 (381.61 to 697.27) | 94665.03 (64841.94 to 135619.43) | 226.36 (149.09 to 333.73) | -56.16 (-62.15 to -50.48) |
| Romania | 65715.26 (48625.55 to 87952.11) | 260.51 (190.79 to 354.12) | 36814.49 (25024.2 to 52756.51) | 176.01 (114.22 to 260.57) | -32.44 (-44.15 to -20.42) |
| Serbia | 24716.29 (19473.03 to 31463.21) | 245.99 (193.15 to 316.53) | 15682.8 (11885.9 to 20507.63) | 149.18 (107.35 to 206.23) | -39.35 (-50.08 to -29.43) |
| Slovakia | 8679.1 (6392.91 to 11939.04) | 159.34 (115.97 to 220.82) | 7224.8 (4955.97 to 10216.85) | 127.09 (83.99 to 186.67) | -20.24 (-29.95 to -11.76) |
| Slovenia | 5901.19 (4141.76 to 8135.07) | 275.45 (191.33 to 384.75) | 4241.18 (2796.83 to 6139.36) | 187.58 (120.42 to 281.13) | -31.9 (-39.31 to -24.71) |
| Belarus | 43146.62 (33703.04 to 55114.45) | 365.96 (283.16 to 470.24) | 15366.7 (10345.16 to 21798.16) | 152.27 (100.85 to 222.09) | -58.39 (-67.2 to -49.46) |
| Estonia | 3730.02 (3049.12 to 4557.16) | 208.25 (166.86 to 258.14) | 1536.5 (1145.15 to 2025.57) | 103.88 (72.29 to 147.07) | -50.12 (-59.4 to -41.11) |
| Latvia | 9526.43 (7454.26 to 12147.67) | 309.35 (238.33 to 401.4) | 2997.26 (2123.55 to 4115.35) | 144.01 (97.39 to 206.98) | -53.45 (-62.04 to -44.9) |
| Lithuania | 7952.69 (6078.95 to 10339.49) | 197.5 (148.63 to 260.16) | 3560.73 (2534.91 to 4922.39) | 121.04 (82.44 to 176.8) | -38.71 (-49.32 to -27.91) |
| Republic of Moldova | 9826.21 (7390.55 to 12868.26) | 218.38 (164.07 to 285.92) | 4484.15 (3061.17 to 6340.27) | 120.4 (79.16 to 179.03) | -44.87 (-54.55 to -35.14) |
| Russian Federation | 523864.47 (415101.85 to 660889.34) | 312.45 (244.22 to 399.65) | 176660.75 (126447.21 to 243569.86) | 118.41 (81.22 to 172.5) | -62.1 (-68.13 to -55.68) |
| Ukraine | 126302.69 (88273.47 to 173073.88) | 227.61 (157.9 to 318.44) | 56159.17 (37710.78 to 81008.82) | 138.87 (90.21 to 208.95) | -38.99 (-47.5 to -29.47) |
| Australia | 96895.74 (71449.24 to 129113.41) | 576.54 (421.26 to 775.18) | 88700.09 (60369.92 to 126893.54) | 373.83 (245.52 to 549.84) | -35.16 (-46.04 to -22.6) |
| New Zealand | 23864.68 (17123.74 to 32925.7) | 681.69 (485.37 to 951.8) | 12366.43 (8614.58 to 17551.86) | 283.87 (193.01 to 416.2) | -58.36 (-62.13 to -54.26) |
| Brunei Darussalam | 852.36 (661.67 to 1075.07) | 681.43 (534.11 to 818.08) | 966.06 (719.35 to 1315.13) | 327.48 (255.87 to 419.8) | -51.94 (-60.59 to -41.13) |
| Japan | 556143.43 (406081.39 to 752327.8) | 401.27 (289.81 to 546.61) | 222042.07 (151711.39 to 311870.03) | 161.15 (104.8 to 241.75) | -59.84 (-65.12 to -54.58) |
| Republic of Korea | 173434.98 (132844.6 to 210583.71) | 645.6 (465.16 to 764.32) | 103986.57 (76945.64 to 139186.65) | 178.52 (126.09 to 251.8) | -72.35 (-79.18 to -60.84) |
| Singapore | 11509.51 (8764.19 to 15034.19) | 415.84 (319.01 to 536.92) | 8837.69 (5943.03 to 12756.96) | 159.05 (104.46 to 235.47) | -61.75 (-69.08 to -53.7) |
| Canada | 68555.67 (49185.27 to 95588.24) | 254.23 (178.76 to 361.32) | 69837.95 (47224.04 to 100710.16) | 201.86 (133.08 to 301) | -20.6 (-32.42 to -12.58) |
| Greenland | 280.97 (208.37 to 379.51) | 580.9 (435.29 to 772.26) | 181.12 (130.82 to 248.42) | 316.82 (225.1 to 441.04) | -45.46 (-53.06 to -36.59) |
| United States of America | 1051197.41 (733340.81 to 1478600.89) | 417.47 (287.93 to 591.91) | 1414555.05 (974633.77 to 1957879.68) | 436.01 (298.1 to 616.08) | 4.44 (-3.45 to 12.86) |
| Argentina | 116514.79 (83460.22 to 159741.32) | 352.49 (252.88 to 482.76) | 137569.73 (93582 to 198020.36) | 297.63 (200.47 to 433.35) | -15.56 (-24.99 to -5.67) |
| Chile | 31782.1 (22708.76 to 44408.59) | 265.98 (193.15 to 364.44) | 42643.72 (29417.6 to 60267.12) | 221.58 (150.75 to 318.99) | -16.69 (-25 to -8.21) |
| Uruguay | 14080.04 (10979.97 to 17948.49) | 412.59 (319.25 to 530.08) | 10354.99 (7629.57 to 14023.18) | 273.98 (195.85 to 382.17) | -33.59 (-41.22 to -25.81) |
| Andorra | 175.99 (119.87 to 251.96) | 316.06 (215.9 to 450.06) | 217.2 (146.61 to 315.96) | 246.05 (163.97 to 361.1) | -22.15 (-29.77 to -14.96) |
| Austria | 31622.54 (23039.05 to 42773.77) | 343.21 (246.73 to 472.69) | 20179.75 (13439.07 to 29407.81) | 212.86 (138.33 to 318.2) | -37.98 (-46.81 to -29.9) |
| Belgium | 43119.54 (31723.87 to 58381.5) | 364.92 (265.7 to 501.47) | 23702.12 (16432.71 to 33248.13) | 196.05 (131.16 to 285.57) | -46.28 (-54.03 to -38.27) |
| Cyprus | 3095.15 (2175.43 to 4272.52) | 406.71 (289.04 to 558.36) | 4237.09 (2832.16 to 6127.03) | 299.36 (199.19 to 439.94) | -26.39 (-36.61 to -17.8) |
| Denmark | 18923.28 (14008.21 to 25205.88) | 318.11 (231.69 to 432.14) | 12225.71 (8318.69 to 17633.36) | 201.83 (133.39 to 295.31) | -36.55 (-45.14 to -27.71) |
| Finland | 16159.38 (11232.86 to 22817.46) | 294.12 (201.33 to 422.74) | 16007.22 (10915.97 to 22499.12) | 250.26 (163.9 to 365.94) | -14.91 (-22.15 to -7.99) |
| France | 289898.54 (205937.9 to 396710.7) | 442.74 (311.18 to 615.72) | 195295.43 (132742.95 to 280864.63) | 272.36 (180.15 to 401.39) | -38.48 (-46.03 to -30.09) |
| Germany | 390372.84 (299288.57 to 513743.59) | 391.17 (295.58 to 523.01) | 160821.46 (111734.75 to 226618.63) | 177.2 (117.38 to 258.59) | -54.7 (-62.44 to -47.17) |
| Greece | 30025.69 (20528.72 to 43044.98) | 253.14 (170.53 to 370.4) | 23783.4 (15568.5 to 34956.48) | 212.91 (137.76 to 320.58) | -15.89 (-24.9 to -4.32) |
| Iceland | 1090.41 (754.69 to 1540.8) | 423.32 (292.89 to 595.69) | 1047.58 (684.56 to 1563.92) | 293.81 (189.02 to 442.92) | -30.59 (-38.23 to -23.6) |
| Ireland | 18201.05 (13035.58 to 24792.89) | 490.56 (348.5 to 672.35) | 14421.03 (9721.73 to 21046.05) | 286.28 (190.41 to 424.02) | -41.64 (-48.3 to -35.2) |
| Israel | 17424.38 (12720.88 to 23551.19) | 357 (261.29 to 481.84) | 20568.48 (14044.23 to 29635.28) | 216.41 (146.36 to 314.47) | -39.38 (-46.25 to -32.44) |
| Italy | 185488.16 (133569.77 to 253608.92) | 260.13 (185.04 to 360.52) | 105665.29 (70050.37 to 154388.38) | 163.71 (103.94 to 245.59) | -37.07 (-45.79 to -28.54) |
| Luxembourg | 1998.31 (1453.75 to 2764.09) | 464.24 (332.46 to 649.33) | 1902.26 (1286.24 to 2714.06) | 286.67 (190.6 to 415.18) | -38.25 (-46.64 to -32.3) |
| Malta | 1581.61 (1096.31 to 2209.24) | 399.61 (276.77 to 564.16) | 1425.39 (940.96 to 2023.85) | 287.08 (185.95 to 424.18) | -28.16 (-36.72 to -19.44) |
| Monaco | 89.05 (61.17 to 126.78) | 262.51 (174.98 to 387.38) | 101.31 (68.31 to 143.92) | 247.95 (163.5 to 366.14) | -5.55 (-11.48 to 0.44) |
| Netherlands | 56488.71 (37082.42 to 83273.59) | 347.79 (228.53 to 515.46) | 53135.4 (34849.96 to 77424.4) | 278.6 (178.48 to 410.28) | -19.89 (-29.02 to -4.34) |
| Norway | 28196.86 (20830.23 to 37568.84) | 539.59 (389.99 to 737.07) | 15879.16 (10652.36 to 22942.11) | 282.87 (185.7 to 420.22) | -47.58 (-53.69 to -42.11) |
| Portugal | 58508.84 (41225.66 to 83138.03) | 513.21 (356.97 to 726.81) | 44272.81 (29445.18 to 64047.14) | 364.35 (236.94 to 545.41) | -29.01 (-38.28 to -18.01) |
| San Marino | 61.75 (41.11 to 89.4) | 249.62 (165.3 to 368.55) | 82.53 (55.75 to 122.07) | 238.18 (156.31 to 355.51) | -4.58 (-11.31 to 2.65) |
| Spain | 102579.15 (74287.94 to 141429.58) | 233.74 (166.45 to 329.55) | 99635.76 (69160.31 to 140373.24) | 189.85 (126.03 to 277.08) | -18.78 (-28.8 to -5.02) |
| Sweden | 52972.8 (37183.06 to 74472.81) | 500.65 (343.54 to 719.42) | 33946.19 (22212.9 to 49833.64) | 309.33 (197.73 to 464.87) | -38.21 (-44.85 to -31.24) |
| Switzerland | 30073.97 (21130.82 to 41581.64) | 384.25 (267.68 to 544.6) | 24985.59 (16466.51 to 36202.8) | 268.58 (172.02 to 399.05) | -30.1 (-38.96 to -20.61) |
| United Kingdom | 389187.18 (274702.5 to 546053.98) | 623.19 (433.44 to 880.48) | 275562.43 (188370.13 to 395026.06) | 387.04 (255.47 to 566.1) | -37.89 (-41.32 to -34.44) |
| Bolivia (Plurinational State of) | 45257.25 (29286.95 to 64150.98) | 556.27 (390.32 to 751.54) | 28563.37 (19505.44 to 40943.81) | 227.69 (160.47 to 320.08) | -59.07 (-71.05 to -44.39) |
| Ecuador | 46457.97 (31764.43 to 62289.31) | 381.81 (268.41 to 501.26) | 30722.37 (19605.62 to 47864.5) | 174.67 (112.01 to 270.78) | -54.25 (-65.76 to -37.81) |
| Peru | 91945.33 (63880.79 to 128008.91) | 353.15 (253.11 to 482.09) | 62325.06 (39485.02 to 96672.51) | 189.8 (119.9 to 295.31) | -46.26 (-58.87 to -33.42) |
| Antigua and Barbuda | 172.68 (122.7 to 245.54) | 283 (203.74 to 394.78) | 216.02 (151.23 to 303.91) | 270.79 (184.99 to 389.57) | -4.31 (-12.95 to 3.73) |
| Bahamas | 809.51 (592.89 to 1117.73) | 328.18 (249.13 to 437.94) | 1007.53 (723.63 to 1380.77) | 287.65 (202.93 to 409.17) | -12.35 (-22.16 to -2.93) |
| Barbados | 967.95 (727.27 to 1280.88) | 378.73 (282.91 to 505.69) | 999.14 (731.41 to 1338.96) | 345.01 (240.35 to 482.98) | -8.9 (-19.08 to 2.14) |
| Belize | 751.63 (551.19 to 1018.64) | 361.52 (274.48 to 480.1) | 1291.63 (923.63 to 1793.29) | 313.66 (227.91 to 430.01) | -13.24 (-23.17 to -3.42) |
| Bermuda | 167.89 (122.75 to 227.13) | 302.16 (216.88 to 414.27) | 128.27 (89.34 to 181.23) | 254.94 (165.99 to 380.98) | -15.63 (-27.02 to -5.38) |
| Cuba | 42090.08 (29965.22 to 57741.05) | 392.59 (279.22 to 550.06) | 33006.39 (23467.14 to 45686.32) | 325.93 (221.63 to 471.37) | -16.98 (-25.3 to -8.86) |
| Dominica | 324.21 (247.63 to 427.78) | 435.67 (336.84 to 566.39) | 280.45 (214.44 to 362.46) | 410.41 (308.81 to 540.34) | -5.8 (-18.18 to 5.87) |
| Dominican Republic | 36592.43 (27853.86 to 45464.54) | 501.97 (397.24 to 607.86) | 31385.28 (23320.11 to 40764.21) | 297.9 (221.65 to 385.06) | -40.65 (-52.93 to -28.46) |
| Grenada | 361.19 (262.08 to 492.13) | 413.58 (308 to 545.8) | 329.31 (229.08 to 468.77) | 337.91 (229.6 to 486.8) | -18.3 (-27.92 to -9.67) |
| Guyana | 3491.94 (2669.13 to 4572.7) | 503.31 (403.26 to 633.61) | 2951.76 (2180.05 to 3918.63) | 389.57 (289.54 to 517.48) | -22.6 (-33.77 to -11.1) |
| Haiti | 87013.67 (57119.04 to 118812.55) | 1286.29 (915.71 to 1690.44) | 88556.13 (63396.98 to 116227.86) | 737.41 (524.8 to 978.64) | -42.67 (-54.17 to -27.97) |
| Jamaica | 9802.13 (7172.55 to 13222.31) | 395.46 (296.79 to 521.21) | 7966.26 (5657.4 to 10947) | 300.78 (210.77 to 425.02) | -23.94 (-33.59 to -13.94) |
| Puerto Rico | 20028.52 (14445.53 to 27583.7) | 557.51 (400.36 to 766.54) | 11603.69 (8134.8 to 16140.97) | 382.7 (253.66 to 557.44) | -31.36 (-39.61 to -23.41) |
| Saint Kitts and Nevis | 156.9 (117.14 to 212.15) | 380.92 (288.23 to 503.45) | 148.04 (102.09 to 207.98) | 275.26 (186.59 to 395.95) | -27.74 (-38.79 to -17.52) |
| Saint Lucia | 688.53 (522.56 to 902.61) | 531.15 (418.81 to 673.75) | 658.37 (498.4 to 860.36) | 391.14 (288.63 to 523.39) | -26.36 (-35.18 to -18.13) |
| Saint Vincent and the Grenadines | 400.32 (293.46 to 550.34) | 366.49 (279.87 to 485.97) | 340.5 (253.26 to 456.16) | 311.56 (224.16 to 427.69) | -14.99 (-23.91 to -6.49) |
| Suriname | 1385.03 (1022.77 to 1871.92) | 362.35 (275.89 to 479.15) | 1635.5 (1190.25 to 2216.86) | 291.66 (209.97 to 401.61) | -19.51 (-28.59 to -10.29) |
| Trinidad and Tobago | 4621.45 (3770.93 to 5755.74) | 405.74 (339.14 to 493.57) | 3667.92 (2714.03 to 4901.24) | 280.49 (201.61 to 389.02) | -30.87 (-43.43 to -16.86) |
| United States Virgin Islands | 327.47 (246.09 to 442.92) | 317.49 (242.4 to 426.28) | 286.35 (214.6 to 379.68) | 277.12 (194.39 to 392.66) | -12.71 (-23.88 to -2.66) |
| Colombia | 90058.05 (65356.86 to 124849.54) | 297.96 (227.57 to 395.44) | 68842.78 (45645.61 to 101423.35) | 153.13 (100.59 to 230.3) | -48.61 (-57.23 to -40.66) |
| Costa Rica | 10400.14 (7080.37 to 14990.33) | 346.09 (244.49 to 481.51) | 11233.48 (7626.41 to 16170.29) | 248.29 (165.9 to 364.91) | -28.26 (-36.85 to -19.87) |
| El Salvador | 35686.74 (25819.83 to 47339.54) | 644.16 (485.4 to 825.36) | 16627.09 (11631.38 to 23502.62) | 268.2 (188 to 376.92) | -58.36 (-66.68 to -48.73) |
| Guatemala | 61770.15 (45391.4 to 76483.64) | 680.43 (522.21 to 817.67) | 31633.04 (22428.52 to 44389.09) | 184.36 (136.18 to 249.6) | -72.9 (-79.21 to -63.57) |
| Honduras | 35148.7 (25655.45 to 45356.44) | 647.48 (492.28 to 806.48) | 27761.87 (19765.96 to 37523.77) | 306.8 (221.33 to 402.59) | -52.62 (-63.13 to -40.83) |
| Mexico | 210448.45 (160470.78 to 281254.15) | 285.39 (232.44 to 358.25) | 172040.54 (120406.32 to 249529.59) | 142.86 (100 to 206.56) | -49.94 (-58.07 to -41.42) |
| Nicaragua | 18974.28 (13890.55 to 25274.1) | 434.61 (336.74 to 554.42) | 12925.04 (8932.4 to 18681.12) | 206.2 (147.17 to 289.03) | -52.56 (-60.84 to -43.49) |
| Panama | 8525.58 (6243.55 to 11492.86) | 342.69 (258.56 to 453.35) | 9094.73 (6300.2 to 12865.21) | 220.69 (152.51 to 312.79) | -35.6 (-45.7 to -25.12) |
| Venezuela (Bolivarian Republic of) | 58068.99 (42468.52 to 80255.98) | 292.93 (222.02 to 394.76) | 53764.8 (37500.98 to 75760.85) | 197.75 (135.98 to 282.61) | -32.49 (-41.73 to -23.75) |
| Brazil | 524951.03 (372390.89 to 753508.27) | 343.06 (253.11 to 477.37) | 448965.4 (310381.49 to 661635.68) | 226.7 (154.22 to 339.01) | -33.92 (-41.31 to -27.24) |
| Paraguay | 10788.79 (7416.15 to 15567.92) | 242.42 (174.17 to 337.9) | 17030.47 (11265.02 to 25471.36) | 250 (167.02 to 370.7) | 3.13 (-7.85 to 13.73) |
| Afghanistan | 105484.45 (76397.21 to 136749.17) | 1246.63 (870.02 to 1671.32) | 153145.24 (115193.32 to 198998.79) | 729.26 (523.15 to 970.06) | -41.5 (-54.09 to -26.63) |
| Algeria | 90377.31 (71284.64 to 113539.19) | 560.17 (434.45 to 703.35) | 100735.36 (77472.71 to 131487.54) | 275.58 (213.56 to 354.19) | -50.8 (-61.55 to -37.35) |
| Bahrain | 1558.01 (1258.58 to 1968.48) | 496.96 (415.84 to 600.09) | 2737.36 (2048.08 to 3699.18) | 234.15 (178.95 to 310.36) | -52.88 (-62.06 to -43.4) |
| Egypt | 344227.11 (273801.5 to 410983.47) | 825.97 (662.19 to 993.53) | 329753.96 (248868.56 to 426178.52) | 427.6 (321.75 to 554.64) | -48.23 (-59.67 to -35.28) |
| Iran (Islamic Republic of) | 200080.09 (159762.53 to 248231.83) | 529.31 (430.99 to 651.97) | 178732.64 (141138.9 to 230621.06) | 232.27 (184.98 to 299.31) | -56.12 (-64.91 to -47.46) |
| Iraq | 67948.76 (53874.77 to 85199.29) | 543.22 (438.29 to 661.67) | 88872.91 (65705.6 to 119964.95) | 255.94 (197.61 to 329.54) | -52.89 (-62.21 to -42.46) |
| Jordan | 9784.26 (7325.16 to 13299.52) | 369.14 (293.36 to 460.63) | 22669.86 (15490.98 to 32674.2) | 212.33 (151.09 to 293.61) | -42.48 (-53.74 to -30.43) |
| Kuwait | 4160.12 (3042.78 to 5747.77) | 299.52 (230.07 to 388.5) | 7773.04 (5137.61 to 11224.94) | 193.6 (128.76 to 279.04) | -35.36 (-46.83 to -23.65) |
| Lebanon | 12522.57 (9550.61 to 15986.03) | 475.43 (368.33 to 607.44) | 14282.53 (10619.64 to 18865.07) | 277.31 (205.82 to 366.03) | -41.67 (-55.46 to -27.74) |
| Libya | 13365.52 (10140.62 to 17361.17) | 464.11 (353.27 to 589.46) | 17393.36 (13105.13 to 22497.46) | 298.07 (226.85 to 386.8) | -35.78 (-49.83 to -18.71) |
| Morocco | 93676.13 (72706.25 to 120107.39) | 540.9 (422.58 to 734.4) | 107938.06 (83303.54 to 135378.19) | 336.9 (263.88 to 424.49) | -37.72 (-50.78 to -23.9) |
| Oman | 2763.37 (1812.56 to 4142.87) | 160.28 (112.27 to 223.96) | 7003.72 (4432.61 to 10629.14) | 169.94 (110.29 to 252.49) | 6.03 (-10.86 to 24.02) |
| Palestine | 5707.32 (4277.86 to 7513.85) | 413.65 (317.07 to 524.51) | 9078.67 (6532.04 to 12744.01) | 227.22 (175.59 to 296.42) | -45.07 (-56.74 to -31.31) |
| Qatar | 788.01 (575.89 to 1086.47) | 235.04 (173.1 to 318.72) | 3683.66 (2461.2 to 5454.19) | 160.9 (109.3 to 230.91) | -31.54 (-47.43 to -18) |
| Saudi Arabia | 39715.62 (30156.72 to 52260.69) | 416.2 (308.33 to 573.12) | 62888.32 (48236.93 to 81569.99) | 228.29 (179.74 to 290.52) | -45.15 (-63.32 to -22.55) |
| Sudan | 141640.6 (101447.92 to 184017.27) | 970.19 (657.05 to 1386.03) | 132501.3 (95276.76 to 175171.55) | 464.32 (335.03 to 638.6) | -52.14 (-61.5 to -38.31) |
| Syrian Arab Republic | 55132.82 (45091.88 to 67419.04) | 627 (504.01 to 770.29) | 49560.31 (39101.51 to 62444.09) | 383.15 (305.03 to 480.9) | -38.89 (-53.42 to -20.11) |
| Tunisia | 27407.39 (21872.16 to 35100.54) | 426.32 (340.76 to 554.81) | 29858.38 (22707.05 to 38756.53) | 255.82 (192.65 to 333.93) | -39.99 (-53.92 to -26.02) |
| Turkey | 214973.16 (165137.95 to 281292.12) | 435.17 (338.41 to 553.26) | 203950.6 (147952.5 to 277542.5) | 253.49 (182.6 to 347.09) | -41.75 (-53.63 to -31.18) |
| United Arab Emirates | 11270.76 (8436.01 to 15311.25) | 1061.78 (784.41 to 1505.99) | 46367.22 (32308.95 to 66889.33) | 554.11 (397.5 to 778.3) | -47.81 (-59.73 to -31.48) |
| Yemen | 80745.81 (59122.06 to 105898.74) | 1033.32 (719.28 to 1502.71) | 107152.07 (82283.42 to 139677.23) | 531.23 (404.62 to 689.11) | -48.59 (-62.8 to -28.17) |
| Bangladesh | 530594.6 (398569.62 to 695693.62) | 870.61 (632.15 to 1225.37) | 336581.37 (253568.7 to 491855.92) | 243.4 (181.94 to 362.6) | -72.04 (-80.18 to -57.4) |
| Bhutan | 2700.3 (1678.19 to 4070.7) | 839.55 (514.81 to 1398.09) | 2004.22 (1450.4 to 2870.85) | 341.82 (247.77 to 490.24) | -59.29 (-72.25 to -38.65) |
| India | 4946069.95 (3335325.25 to 6747398.93) | 977.17 (630.47 to 1427.56) | 5838764.76 (4244934.46 to 7429627.78) | 498.52 (357.5 to 639.83) | -48.98 (-59.33 to -38.17) |
| Nepal | 149258.09 (94000.99 to 199509.14) | 1429.52 (877.35 to 2060.26) | 165506.73 (108362.74 to 230017.67) | 740.99 (482.28 to 1040.92) | -48.16 (-62.56 to -31.23) |
| Pakistan | 477238.89 (367948.4 to 660566.5) | 700.36 (510.04 to 1039.02) | 564729.3 (453492.27 to 709475.38) | 414.78 (330.09 to 544.54) | -40.78 (-55.11 to -22.11) |
| China | 2004543.71 (1501975.05 to 2635017.11) | 209.24 (159.57 to 272.33) | 1413151.56 (1052300.51 to 1918822.31) | 102.81 (72.3 to 147.42) | -50.87 (-64.29 to -38.38) |
| Democratic People's Republic of Korea | 64339.73 (47509.71 to 83424.74) | 361.75 (268.32 to 468.76) | 73231.89 (53618.98 to 95448.33) | 256.52 (188.64 to 336.46) | -29.09 (-42.14 to -14.42) |
| Taiwan (Province of China) | 69494.34 (57199.25 to 85757.86) | 418.56 (351.15 to 503.58) | 41695.73 (30470.54 to 56439.73) | 168.76 (117.5 to 238.02) | -59.68 (-68.07 to -49.95) |
| American Samoa | 174.48 (141.95 to 215.55) | 554.15 (461.47 to 668.74) | 166.52 (130.92 to 212.15) | 334.72 (265.12 to 423.51) | -39.6 (-53.26 to -25.96) |
| Cook Islands | 69.52 (53.52 to 88.23) | 456.84 (359.38 to 566.11) | 48.05 (35.19 to 62.82) | 243.64 (176.51 to 322.48) | -46.67 (-57.55 to -35.53) |
| Fiji | 8980.39 (7294.25 to 10925.48) | 2081.4 (1684.12 to 2534.03) | 7347.39 (5823.99 to 9216.93) | 979.87 (780.24 to 1226.82) | -52.92 (-66.33 to -35.72) |
| Guam | 323.97 (239.51 to 440.56) | 316.23 (247.66 to 404.34) | 365.29 (266.89 to 490.06) | 210.93 (153.96 to 285.97) | -33.3 (-42.73 to -24.83) |
| Kiribati | 1227.77 (1021.62 to 1460.03) | 2630.86 (2187.96 to 3178.83) | 1362.29 (1087.89 to 1703.9) | 1795.09 (1411.44 to 2242.16) | -31.77 (-47.73 to -13.54) |
| Marshall Islands | 285.69 (221.68 to 384.04) | 1163.81 (891.36 to 1631.5) | 299.43 (216.04 to 400.13) | 707.37 (507.63 to 938.82) | -39.22 (-54.45 to -21.55) |
| Micronesia (Federated States of) | 828.41 (638.27 to 1045.53) | 1333.27 (1019.54 to 1786.14) | 534.87 (378.61 to 710.51) | 689.57 (503.94 to 921.35) | -48.28 (-66.13 to -27.22) |
| Nauru | 62.14 (45.65 to 78.76) | 975.76 (733.1 to 1212.48) | 41.09 (27.65 to 54.5) | 591.65 (395.65 to 770.18) | -39.36 (-54.54 to -22.14) |
| Niue | 14.24 (10.78 to 17.47) | 625.36 (475.67 to 769.43) | 6.54 (4.57 to 8.6) | 349.16 (244.68 to 460.23) | -44.17 (-57.59 to -29.77) |
| Northern Mariana Islands | 127.76 (96.48 to 165.43) | 416.95 (336.23 to 514.13) | 104.65 (78.81 to 135.27) | 254.49 (192.34 to 335.97) | -38.96 (-50.1 to -27.18) |
| Palau | 71.34 (56.42 to 89.57) | 613.02 (487.24 to 763.78) | 69.77 (55.37 to 86.77) | 375.76 (300.06 to 462.94) | -38.7 (-52.23 to -22.56) |
| Papua New Guinea | 41834.22 (32424.45 to 54217.25) | 1716.79 (1319.01 to 2334.88) | 73540.96 (55513.26 to 96924.49) | 1250.25 (941.24 to 1697.62) | -27.17 (-42.86 to -7.82) |
| Samoa | 980.82 (743.34 to 1331.39) | 929.85 (695.65 to 1286.59) | 838.42 (646.4 to 1071.53) | 507.54 (399.31 to 644.89) | -45.42 (-62.43 to -25.21) |
| Solomon Islands | 2388.63 (1853.81 to 3076.59) | 1292.61 (961.08 to 1676.54) | 3778.91 (2889.9 to 4771.97) | 933.13 (688.95 to 1182.34) | -27.81 (-46.7 to -5.57) |
| Tokelau | 12.91 (9.79 to 16.77) | 904.26 (684.46 to 1193.67) | 5.44 (4.06 to 7.11) | 410.83 (307.67 to 530.69) | -54.57 (-67.17 to -39.12) |
| Tonga | 384.35 (304.12 to 497.29) | 552.91 (448.25 to 697.14) | 325.87 (247.82 to 410.85) | 360.69 (278.44 to 446.81) | -34.77 (-49.06 to -18.5) |
| Tuvalu | 99.9 (74.98 to 127.74) | 1324.72 (975.46 to 1752.18) | 59.05 (44.99 to 76.3) | 567.65 (435.1 to 730.31) | -57.15 (-69.7 to -39.36) |
| Vanuatu | 1020.55 (708.19 to 1448.42) | 1147.56 (778.29 to 1712.62) | 1659.22 (1218.53 to 2287.2) | 791.35 (577.48 to 1103.54) | -31.04 (-48.11 to -8.03) |
| Cambodia | 49435.01 (37804.21 to 60137.71) | 696.57 (531.05 to 827.62) | 48944.67 (39096.15 to 60141.74) | 368.41 (296.96 to 448.67) | -47.11 (-58.01 to -33.33) |
| Indonesia | 1116448.24 (914322.15 to 1293965.19) | 797.62 (661.05 to 954.67) | 1044984.01 (853095.56 to 1263065.26) | 455.62 (372.92 to 549.47) | -42.88 (-52.13 to -32.76) |
| Lao People's Democratic Republic | 51693.82 (36877.74 to 67652.89) | 1554.24 (1146.86 to 1932.48) | 30179.27 (22655.32 to 38816.88) | 561.99 (421.88 to 729.9) | -63.84 (-73.82 to -49.54) |
| Malaysia | 66041.02 (53604.36 to 78907.37) | 531.04 (439.86 to 623.84) | 56918.74 (43122.1 to 73572.14) | 192.92 (146.17 to 251.06) | -63.67 (-71.8 to -52.66) |
| Maldives | 1199.59 (831.96 to 1643.74) | 686.58 (485.22 to 902.7) | 660.23 (485.32 to 900.22) | 167.46 (125.38 to 222.35) | -75.61 (-83.03 to -64.26) |
| Mauritius | 7648.05 (6717.48 to 8565.78) | 940.21 (825.24 to 1044.36) | 4249.09 (3385.75 to 5325.67) | 301.65 (237.5 to 374.22) | -67.92 (-73.8 to -58.97) |
| Myanmar | 547268.69 (399441.13 to 696313.4) | 1859.79 (1293.91 to 2341.73) | 313651.1 (238290.79 to 390682.02) | 664.49 (500.43 to 822.04) | -64.27 (-73.6 to -51.95) |
| Philippines | 497293 (412758.13 to 594827.73) | 989.6 (858.59 to 1143.55) | 576672.89 (479743.04 to 693345.76) | 589 (493.03 to 696.54) | -40.48 (-48.83 to -30.18) |
| Seychelles | 237.98 (197.23 to 293.19) | 379.13 (313.51 to 471.15) | 237.15 (183.65 to 298.56) | 232.33 (178.5 to 296) | -38.72 (-48.53 to -29.18) |
| Sri Lanka | 166276 (142216.26 to 186780.02) | 1524.52 (1308.93 to 1708.21) | 166925 (124625.4 to 211782.18) | 726.95 (539.66 to 914.93) | -52.32 (-64.62 to -38.63) |
| Thailand | 201757.85 (160922.04 to 246728.41) | 469.84 (377.04 to 565.82) | 171925.45 (131778.6 to 222440.89) | 225.57 (170.55 to 298.53) | -51.99 (-63.17 to -39.97) |
| Timor-Leste | 6805.46 (4357.98 to 9732.45) | 1096 (789.16 to 1414.05) | 5657.14 (3989.33 to 7396.61) | 542.73 (394.04 to 724.39) | -50.48 (-64.61 to -32.38) |
| Viet Nam | 247687.04 (136441.32 to 322503.33) | 508.24 (248.38 to 680.44) | 254205.01 (164896.32 to 324341.73) | 282.28 (183.46 to 357.84) | -44.46 (-62.83 to -22.53) |
| Angola | 77299.57 (55991.17 to 104121.32) | 1015.44 (742.56 to 1386.63) | 91585.82 (67634.75 to 117202.08) | 449.9 (338.18 to 600.93) | -55.69 (-65.79 to -42.66) |
| Central African Republic | 23403.65 (16771.25 to 29743.27) | 1277.2 (883.06 to 1818.08) | 33206.29 (22904.92 to 46077.54) | 985.01 (641.63 to 1640.9) | -22.88 (-42.1 to 2.62) |
| Congo | 15280.83 (11762.16 to 19737.28) | 955.97 (702.93 to 1382.29) | 17376.92 (12767.45 to 22647.75) | 457.92 (333.99 to 597.6) | -52.1 (-63.85 to -36.2) |
| Democratic Republic of the Congo | 241367.75 (167618.29 to 315851.36) | 873.54 (583.96 to 1305.15) | 330097.53 (227076.79 to 486931.68) | 605.29 (386.94 to 1036.18) | -30.71 (-48.3 to -8.14) |
| Equatorial Guinea | 3294.37 (2295.76 to 4277.69) | 1092.63 (739.42 to 1657.26) | 3054.72 (2103.58 to 4292.13) | 329.14 (225.86 to 516.34) | -69.88 (-79.92 to -54.95) |
| Gabon | 4607.96 (3551.1 to 5712.22) | 627.59 (481.72 to 801.55) | 4222.9 (3112.4 to 5475.93) | 307.1 (230.42 to 392.63) | -51.07 (-63.3 to -35.31) |
| Burundi | 55095.4 (37753.5 to 74434.94) | 1171.56 (825.74 to 1498.76) | 53167.16 (38664.52 to 73604.48) | 590.55 (423.09 to 865.37) | -49.59 (-62.84 to -30.45) |
| Comoros | 2722.25 (1545.52 to 3792.96) | 734.19 (446.71 to 1001.37) | 2603.49 (1951.47 to 3390.63) | 418.34 (319.26 to 542.97) | -43.02 (-57.14 to -6.63) |
| Djibouti | 2404.77 (1788.17 to 3196.09) | 654.77 (480.38 to 845.73) | 3729.41 (2642.9 to 4931.1) | 379.96 (264.6 to 497.39) | -41.97 (-54.23 to -25.71) |
| Eritrea | 23890.02 (16455.86 to 31723.79) | 1046.46 (722.06 to 1349.62) | 27914.06 (20649 to 36722.79) | 566.11 (412.56 to 744.46) | -45.9 (-58.31 to -27.11) |
| Ethiopia | 315568.97 (222614.26 to 393204.81) | 804.38 (595.58 to 999.14) | 257986.06 (196329.69 to 339515.16) | 314.01 (245.38 to 431.58) | -60.96 (-69.06 to -44.26) |
| Kenya | 75338.5 (56425.91 to 99647.08) | 456.64 (327.21 to 682.62) | 129123.11 (94816.38 to 180149.24) | 347.84 (251.59 to 523.64) | -23.83 (-34.82 to -11.43) |
| Madagascar | 148679.06 (120822.13 to 180647.68) | 1317.19 (1084.31 to 1569.41) | 168468.02 (128491.98 to 217802.79) | 783.82 (593.06 to 1003.59) | -40.49 (-51.87 to -27.01) |
| Malawi | 52596.27 (36843.46 to 70071.59) | 644.93 (482.11 to 791.18) | 57673.89 (43989.58 to 75179.59) | 410.96 (317.01 to 522.12) | -36.28 (-49.82 to -16.26) |
| Mozambique | 75192 (52646.6 to 102537.66) | 631.36 (464.23 to 788.33) | 105284.19 (78682.37 to 135902.71) | 469.54 (359.79 to 600.07) | -25.63 (-41.43 to -4.13) |
| Rwanda | 85844.47 (61935.33 to 111541.74) | 1430.29 (1064.2 to 1804.8) | 67352.8 (48879.87 to 90616.23) | 637.61 (471.79 to 860.02) | -55.42 (-65.11 to -39.76) |
| Somalia | 65283.82 (42058.4 to 88796.82) | 1201.81 (757.36 to 1627.73) | 112049.1 (77922.4 to 162912.65) | 816 (540.35 to 1313.42) | -32.1 (-48.42 to -9.13) |
| South Sudan | 38766.82 (27014.73 to 52908.52) | 770.56 (547.02 to 994.52) | 34556.84 (25850.54 to 46293.52) | 465.95 (343.37 to 659.65) | -39.53 (-53.92 to -19.97) |
| Uganda | 111661.5 (78564.5 to 147702.38) | 784.91 (540.29 to 1064.4) | 154512.31 (114190.66 to 206702.61) | 482.3 (364.43 to 648.81) | -38.55 (-51.91 to -19.64) |
| United Republic of Tanzania | 134352.66 (103621.15 to 172519.04) | 604 (472.37 to 742.66) | 227459.76 (167651.24 to 300831.42) | 455.66 (347.11 to 587.01) | -24.56 (-37.06 to -10.11) |
| Zambia | 39896.67 (28491.76 to 52688.31) | 643.4 (504.96 to 777) | 47523.88 (36794.93 to 60496.84) | 365.36 (285.13 to 457.51) | -43.21 (-56.1 to -26.11) |
| Botswana | 6944.84 (5060.77 to 9401.05) | 907.97 (656.17 to 1252.58) | 8548.41 (5978.04 to 11450.87) | 479.47 (335.39 to 639.26) | -47.19 (-66.4 to -23.85) |
| Eswatini | 4841.75 (3712.95 to 6217.31) | 1026.81 (783.64 to 1366.13) | 5234.55 (4001.75 to 6617.15) | 653.37 (501.29 to 834.43) | -36.37 (-53.57 to -12.51) |
| Lesotho | 11033.88 (7318.26 to 16937.02) | 921.25 (597.58 to 1432.61) | 12086.18 (8029.29 to 17090.93) | 792.64 (527.03 to 1135.15) | -13.96 (-44.03 to 29.19) |
| Namibia | 8056.92 (5871.29 to 11015.43) | 900.62 (659.01 to 1274.99) | 7388.38 (5572.85 to 9542.08) | 429.39 (328.18 to 551.78) | -52.32 (-65.61 to -31.76) |
| South Africa | 228742.14 (192889.65 to 275223.56) | 756.16 (643.75 to 891.52) | 209097.53 (169509.34 to 265379.75) | 414.35 (342.25 to 518.4) | -45.2 (-51.68 to -37.81) |
| Zimbabwe | 34069.92 (28549.88 to 39917.35) | 640.11 (536.59 to 742.38) | 56615.99 (40569.19 to 75381.74) | 631.76 (441.96 to 844.09) | -1.3 (-29.58 to 30.62) |
| Benin | 26060.25 (20666.48 to 31821.78) | 834.14 (656.64 to 1018.2) | 38634.46 (29283.65 to 50620.76) | 457.36 (352.45 to 589.59) | -45.17 (-56.74 to -29.07) |
| Burkina Faso | 39694.69 (30394.05 to 50383.39) | 548.41 (436.74 to 662.13) | 66573.45 (50923.4 to 86896.72) | 389.52 (311.84 to 483.67) | -28.97 (-43.19 to -11.9) |
| Cabo Verde | 1497.27 (1054.98 to 1807.11) | 559.42 (379.48 to 667.03) | 1122.82 (876.43 to 1434.82) | 224.17 (176 to 283.66) | -59.93 (-69.96 to -32.81) |
| Cameroon | 48518.2 (39383 to 58615.64) | 760.35 (624.51 to 913.54) | 76874.63 (57784.21 to 99461.68) | 395.85 (301.77 to 514.71) | -47.94 (-61.04 to -29.22) |
| Chad | 34125.03 (26017.16 to 43350.5) | 852.89 (645.6 to 1100.22) | 56302.98 (43374.74 to 71510.52) | 563.02 (436.18 to 719.98) | -33.99 (-48.7 to -12.51) |
| Côte d'Ivoire | 56170.45 (44146.18 to 69226.77) | 792.78 (620.76 to 964.82) | 80170.09 (60968.15 to 102254.66) | 444.6 (352.98 to 555.64) | -43.92 (-56.51 to -27.95) |
| Gambia | 4220.3 (3153.58 to 5493.62) | 729.6 (547.58 to 944.71) | 6880.7 (5141.61 to 8785.56) | 478.28 (365.18 to 607.3) | -34.45 (-52.09 to -10.03) |
| Ghana | 64240.05 (43137.71 to 79170.4) | 669.94 (447.8 to 817.76) | 92271.49 (65882.05 to 116017.63) | 408.19 (292.65 to 512.89) | -39.07 (-53.93 to -21.38) |
| Guinea | 44062.18 (35174.57 to 53923.7) | 945.42 (748.55 to 1157.9) | 54345.02 (41601.92 to 69592.68) | 622.83 (485.41 to 780.62) | -34.12 (-48.96 to -15.84) |
| Guinea-Bissau | 8014.56 (6168.79 to 10007.82) | 1290.23 (1011.22 to 1587.9) | 7901.39 (6146.22 to 9912.65) | 675.56 (521.88 to 841.49) | -47.64 (-61.13 to -30.3) |
| Liberia | 10127.83 (7620.04 to 13052.52) | 586.9 (470.38 to 708.08) | 11003.35 (7890.02 to 14832.2) | 321.78 (236.48 to 432.45) | -45.17 (-58.93 to -27.09) |
| Mali | 52571.91 (41056 to 63362.5) | 979.23 (688.44 to 1202.1) | 77598.47 (57560.14 to 103189.34) | 602.77 (429.35 to 813.25) | -38.44 (-52.71 to -19.66) |
| Mauritania | 11168 (8423.71 to 13854.81) | 805.48 (607.41 to 987.25) | 11760.89 (8591.85 to 15662.98) | 375.99 (280.75 to 481.52) | -53.32 (-63.54 to -39.49) |
| Niger | 53954.32 (39946.34 to 68549.84) | 996.36 (749.03 to 1252) | 85130.23 (61578.56 to 113711.94) | 572.37 (412.71 to 780.65) | -42.55 (-56.45 to -24.36) |
| Nigeria | 401313.43 (315838.59 to 498448.31) | 613.18 (484.56 to 760.11) | 573192.14 (431715.82 to 739975.4) | 375.79 (297.44 to 460.28) | -38.71 (-50.14 to -25.05) |
| Sao Tome and Principe | 962.13 (762.51 to 1159.14) | 1130.85 (908.5 to 1362.81) | 1000.25 (765.45 to 1258.55) | 723.67 (550.15 to 907.36) | -36.01 (-51.51 to -16.4) |
| Senegal | 37610.35 (28972.56 to 45883.73) | 767.97 (589.62 to 940.06) | 43109.35 (32902.89 to 55926.6) | 412.37 (323.4 to 524.38) | -46.3 (-59.9 to -28.89) |
| Sierra Leone | 21831.32 (16131.33 to 27889.55) | 796.92 (596.64 to 986.22) | 28664.4 (21536.88 to 36841.42) | 490.65 (378.4 to 626.41) | -38.43 (-52.96 to -17.74) |
| Togo | 18256.41 (14774.39 to 22473.54) | 847.12 (680.05 to 1037.93) | 27594.93 (20937.28 to 35928.94) | 496.91 (380.36 to 632.54) | -41.34 (-54.21 to -25.13) |


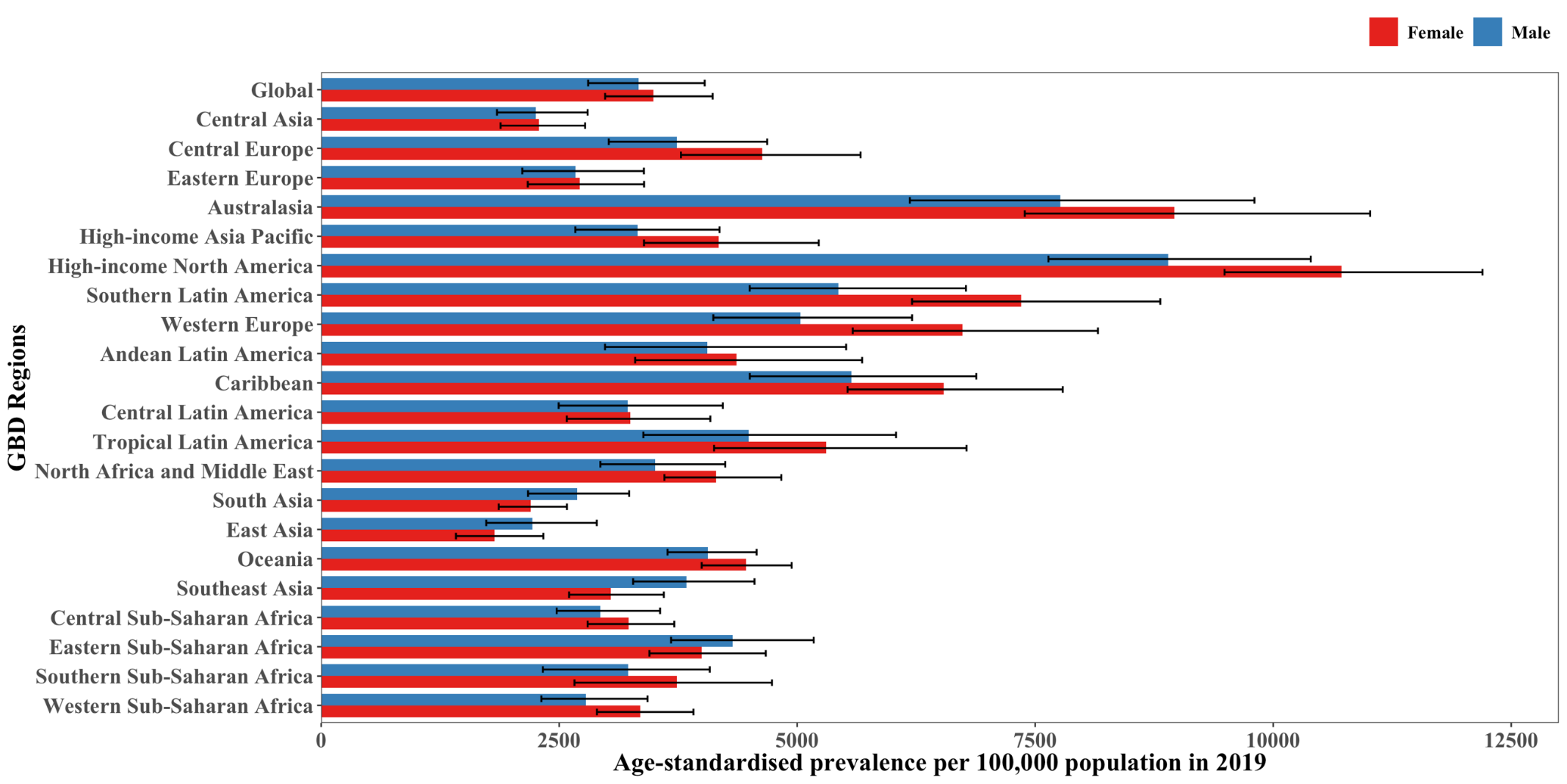


**Figure S1**. The age-standardized prevalence of asthma in 2019 for the 21 Global Burden of Disease regions, by sex.


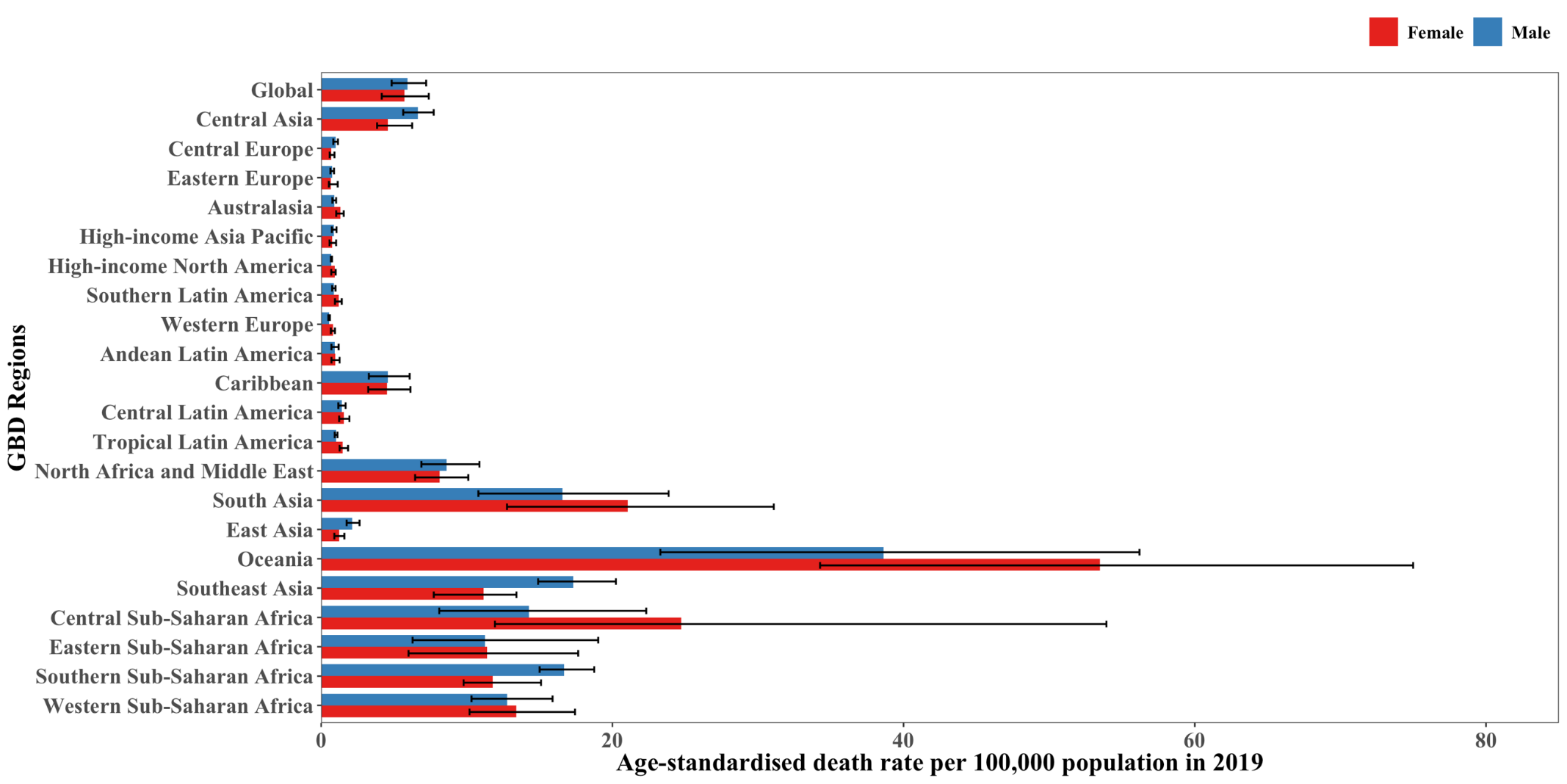


**Figure S2**. The age-standardized deaths rate of asthma in 2019 for the 21 Global Burden of Disease regions, by sex.


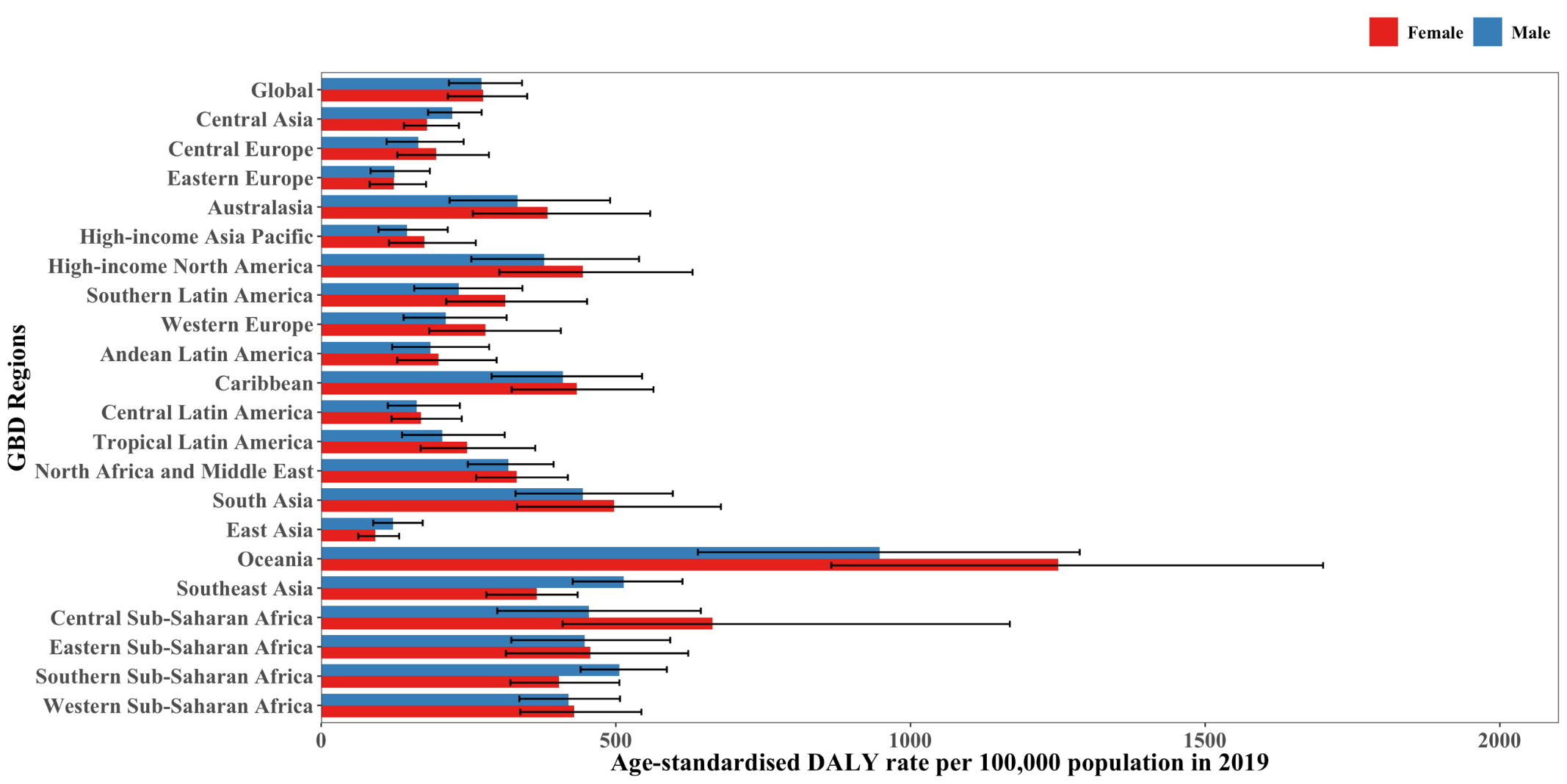


**Figure S3**. The age-standardized DALY rate of asthma in 2019 for the 21 Global Burden of Disease regions, by sex. DALY: disability adjusted life year.


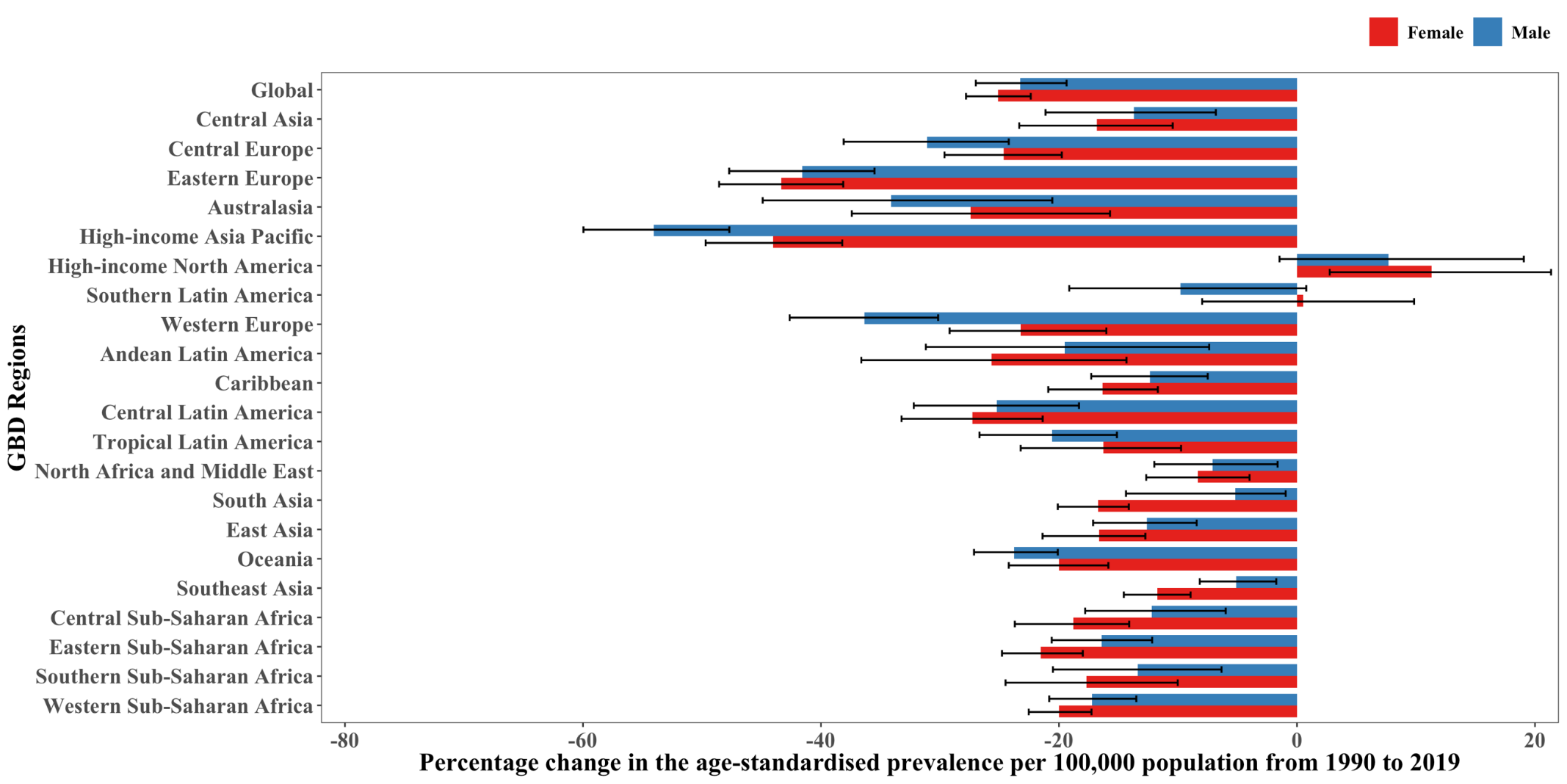


**Figure S4**. The percentage change in the age-standardized prevalence of asthma from 1990 to 2019 for the 21 Global Burden of Disease regions, by sex.


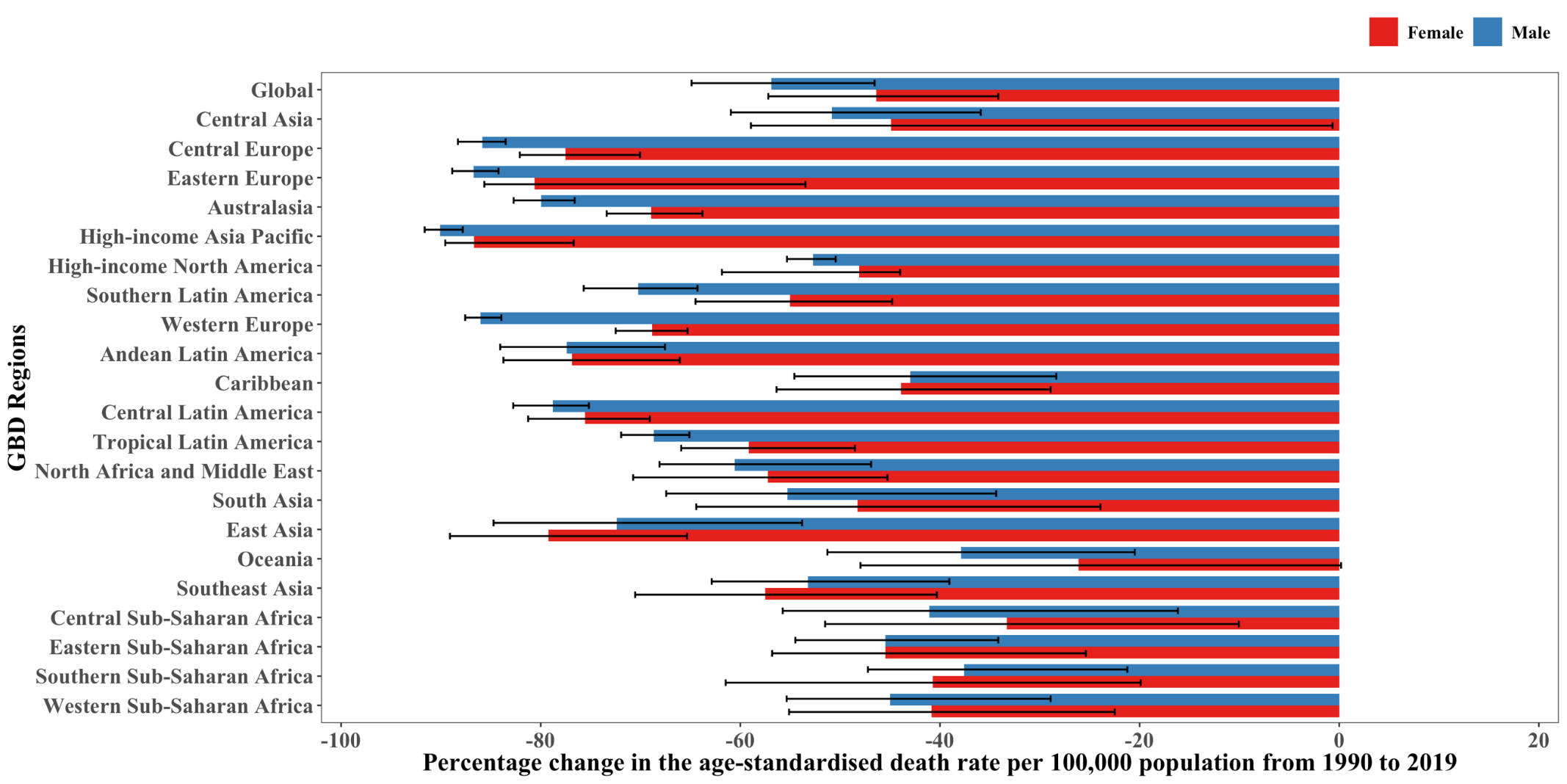


**Figure S5**. The percentage change in the age-standardized death rate of asthma from 1990 to 2019 for the 21 Global Burden of Disease regions, by sex.


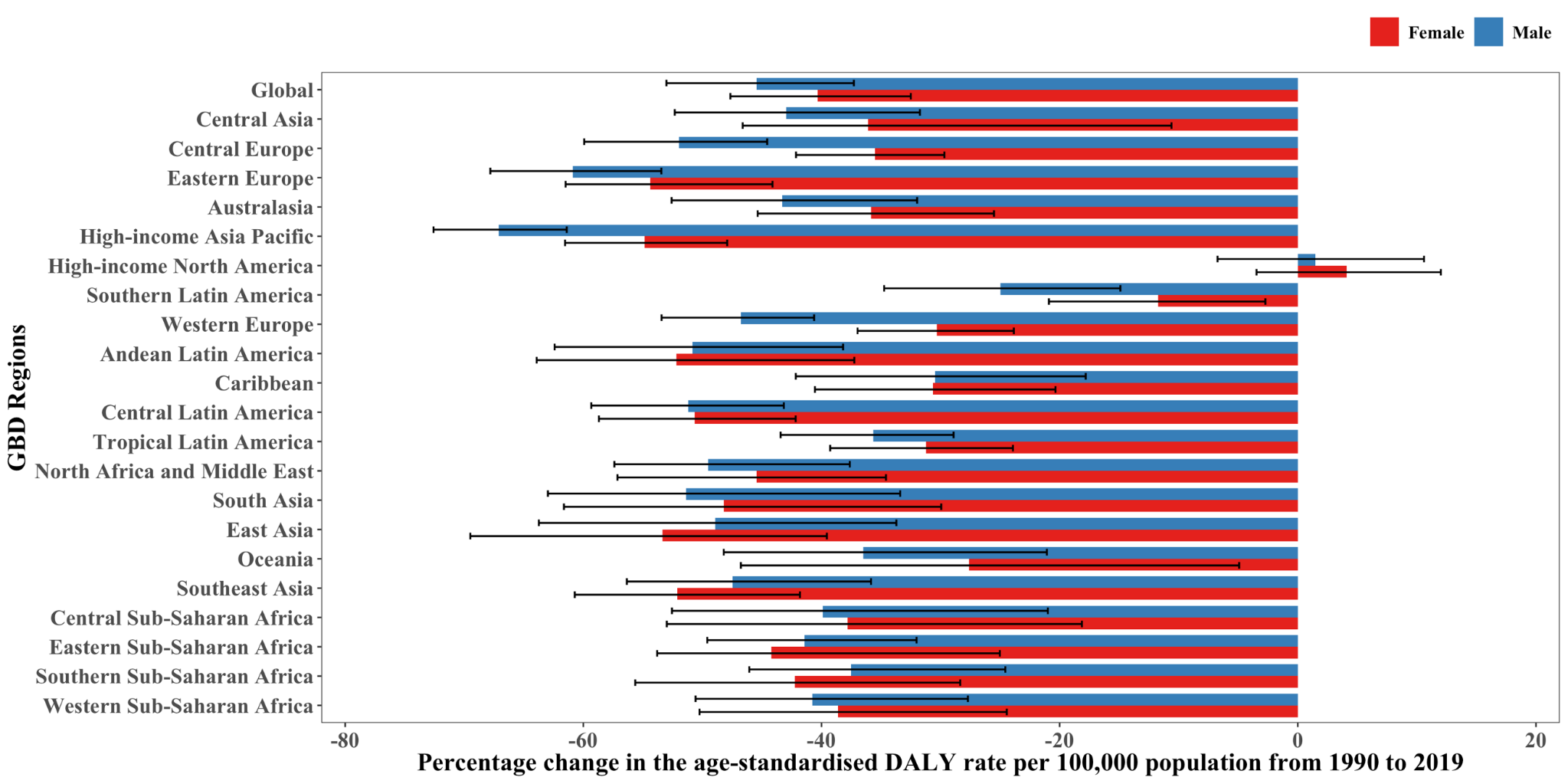


**Figure S6**. The percentage change in the age-standardized DALY rate of asthma from 1990 to 2019 for the 21 Global Burden of Disease regions, by sex. DALY: disability adjusted life year.


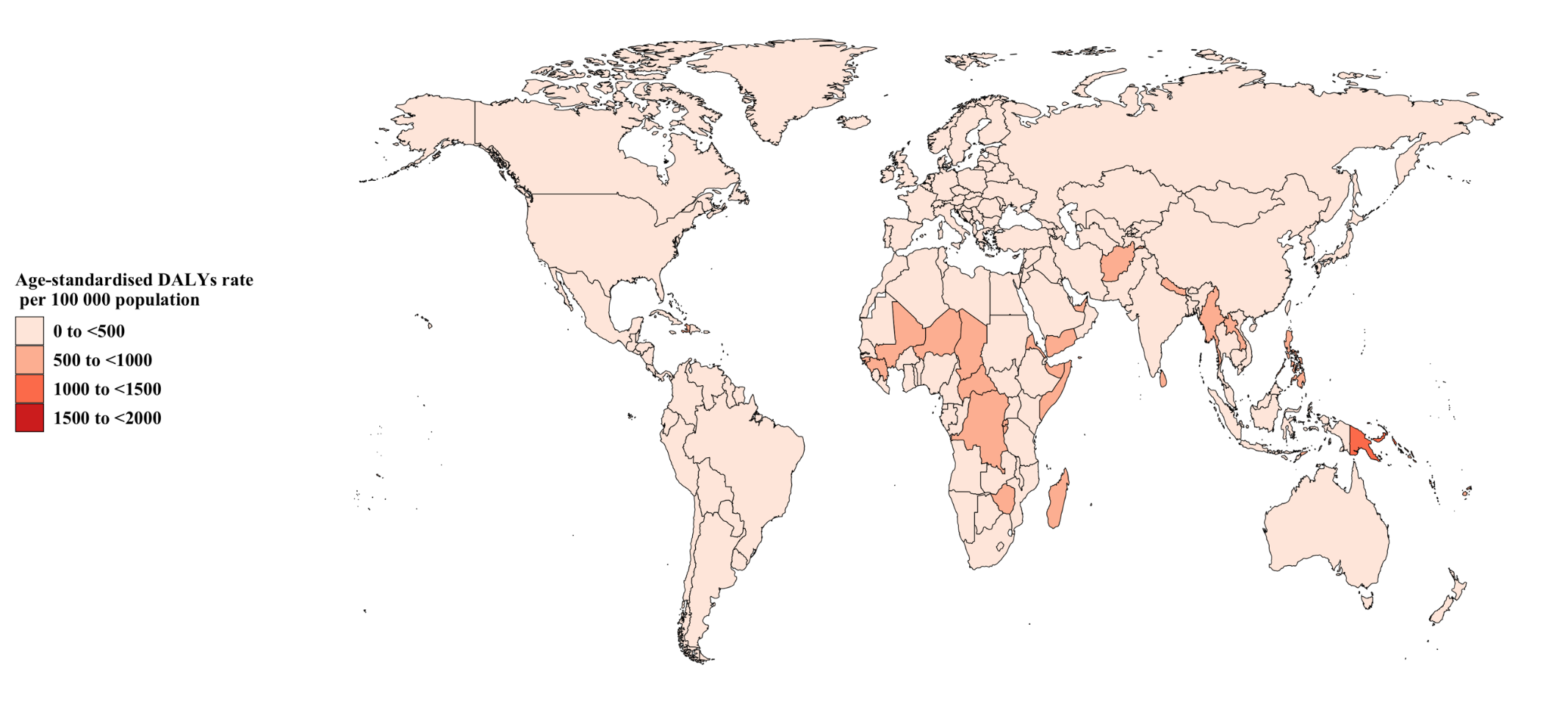


**Figure S7**. Age-standardized disability adjusted life years (DALYs) rate of asthma per 100 000 population in 2019


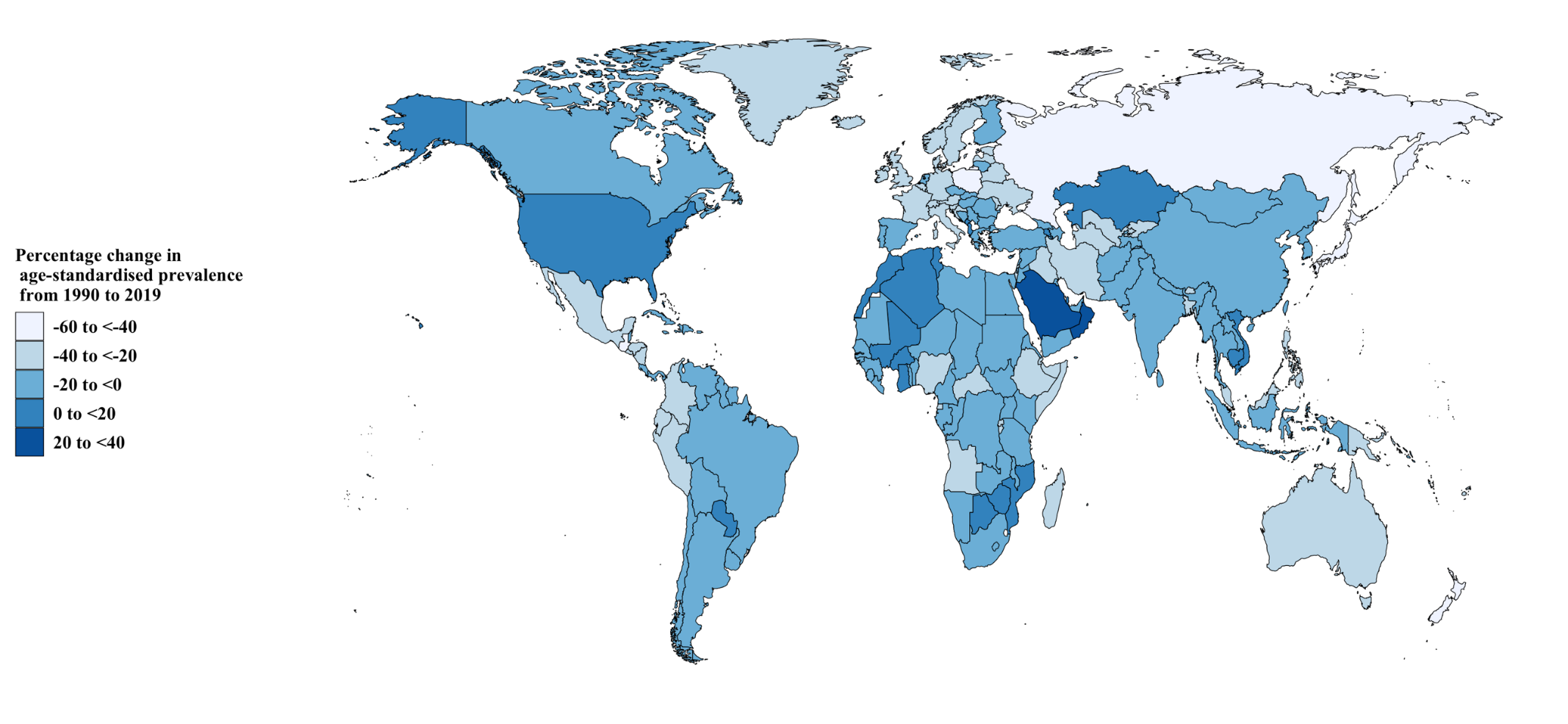


**Figure S8**. The percentage change in the age-standardized prevalence of asthma from 1990 to 2019 for the 204 Global Burden of Disease countries and territories


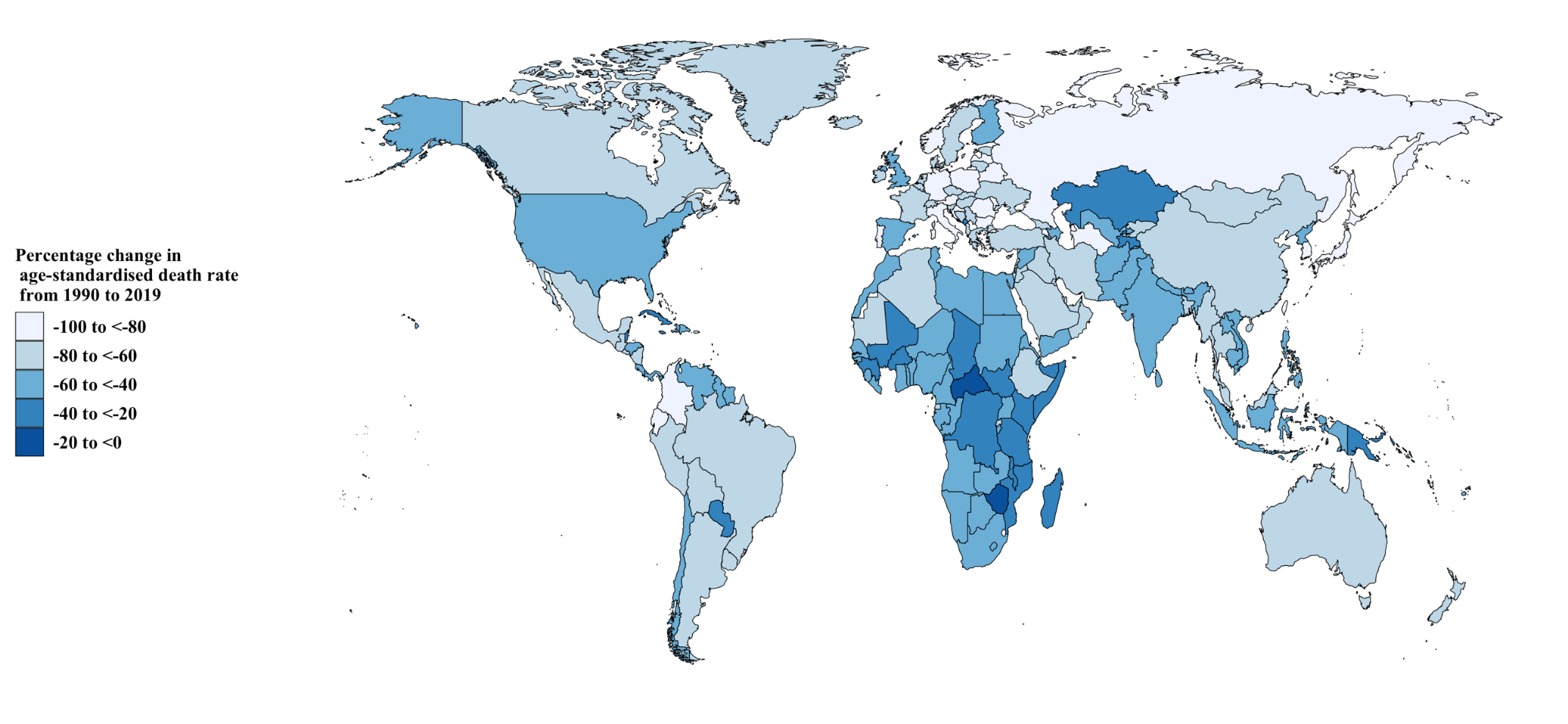


**Figure S9**. The percentage change in the age-standardized death rate of asthma from 1990 to 2019 for the 204 Global Burden of Disease countries and territories


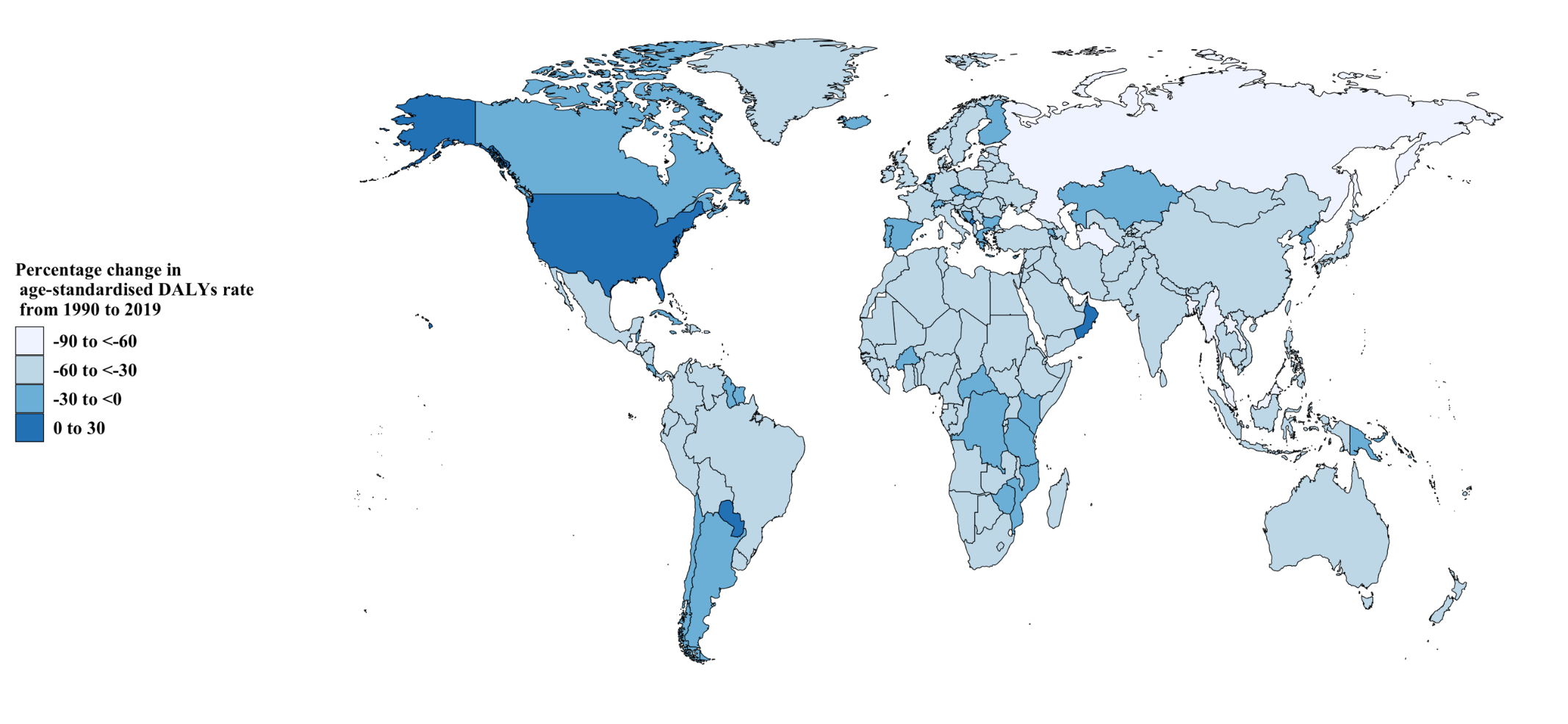


**Figure S10**. The percentage change in the age-standardized DALY rate of asthma from 1990 to 2019 for the 204 Global Burden of Disease countries and territories


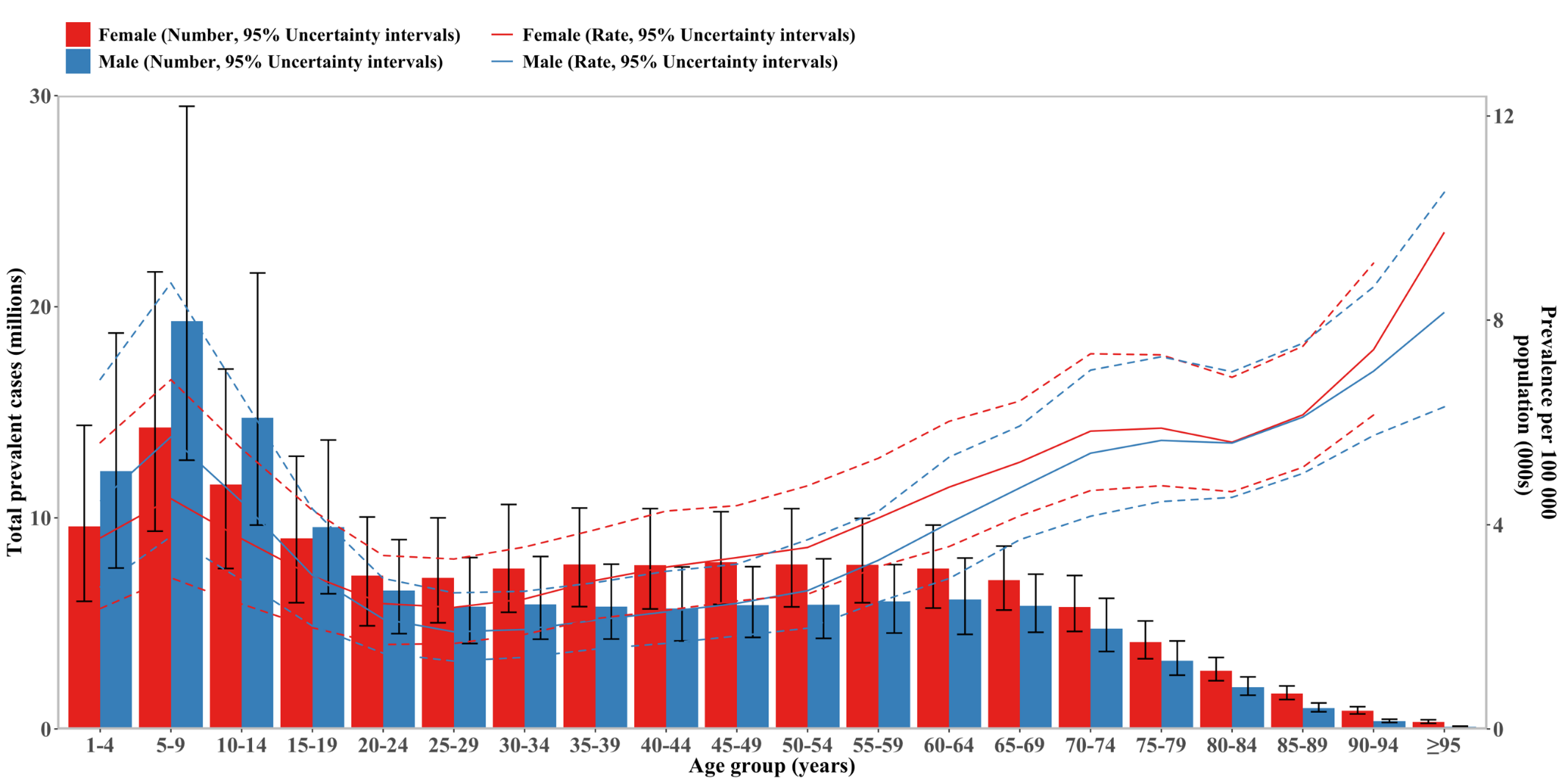
**Figure S11**. Number of prevalent cases globally and prevalence of asthma per 100 000 population, by age and sex in 2019. Boxes indicate prevalent cases with 95% uncertainty intervals for men and women.


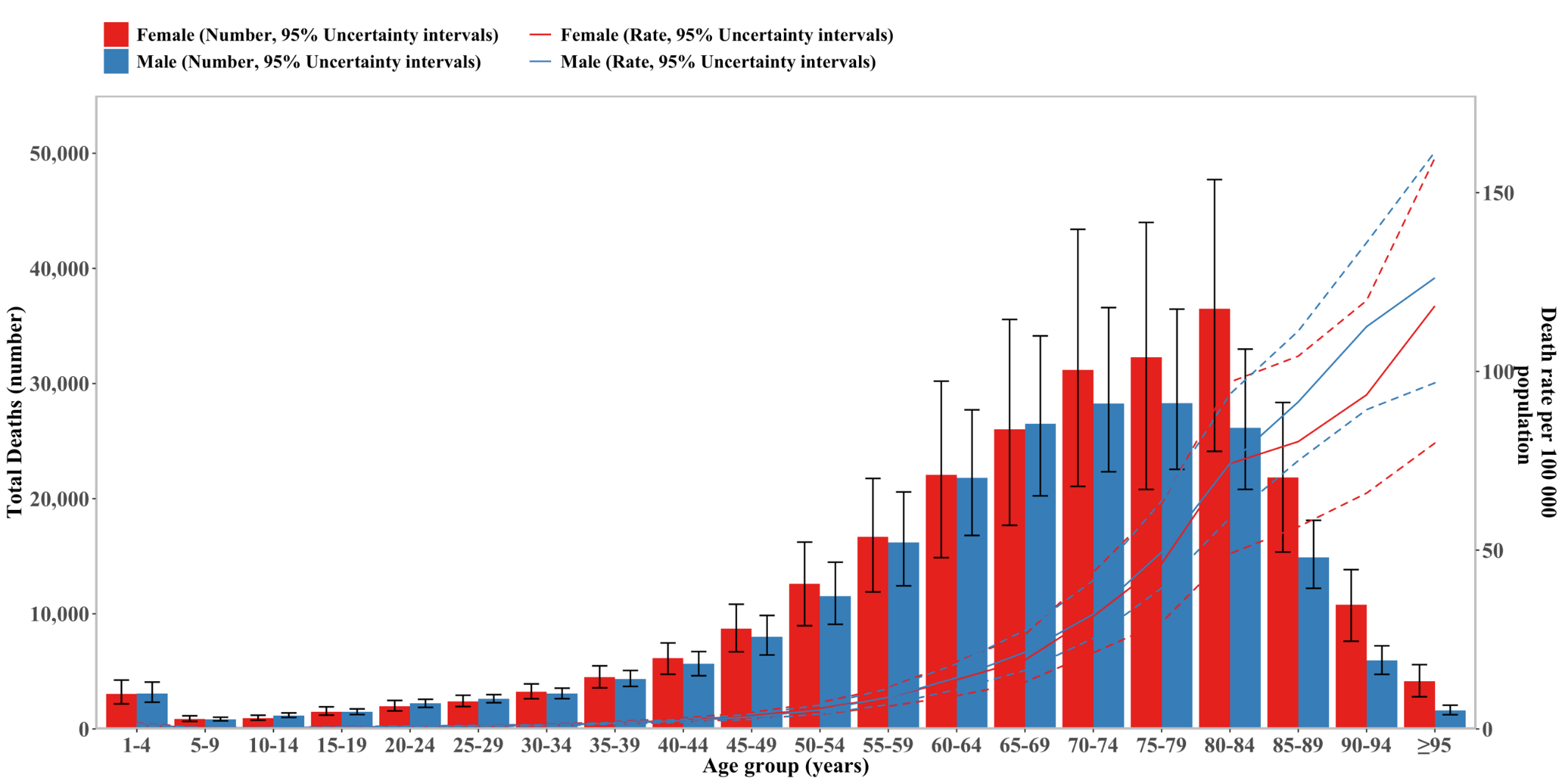


**Figure S12**. Number of death cases globally and death rate of asthma per 100 000 population, by age and sex in 2019. Boxes indicate death cases with 95% uncertainty intervals for men and women.


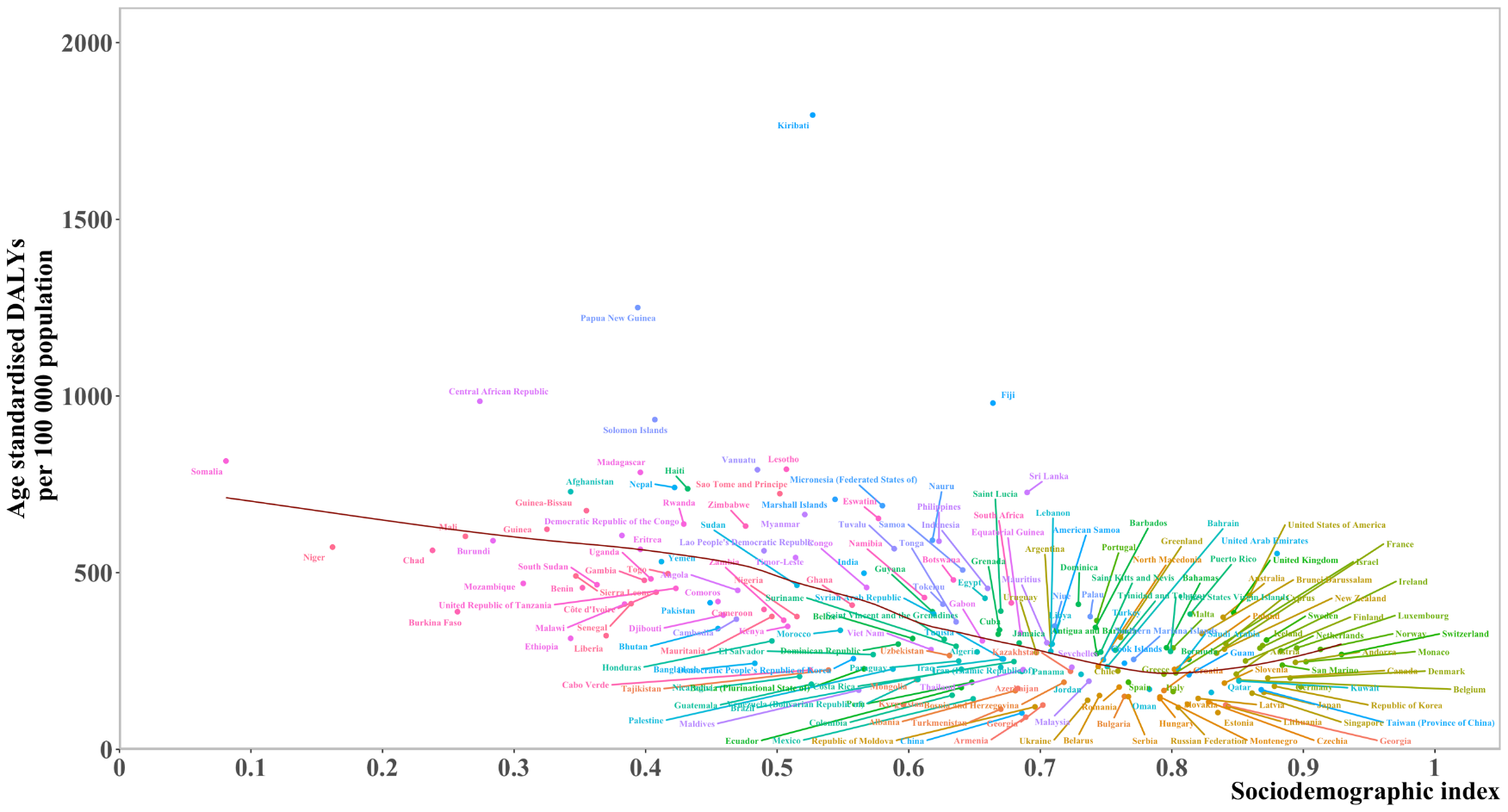


**Figure S13.** Age-standardized disability adjusted life year (DALY) rates of asthma for the 204 Global Burden of Disease countries and territories by sociodemographic index, in 2019. Points are plotted for each country and territory and show the observed age-standardized DALY rates in 2019 for that country or territory. Expected values, based on the sociodemographic index and disease rates in all locations, are shown as a solid line. Countries and territories above the solid line represent a higher than expected burden and countries and territories below the line show a lower than expected burden.


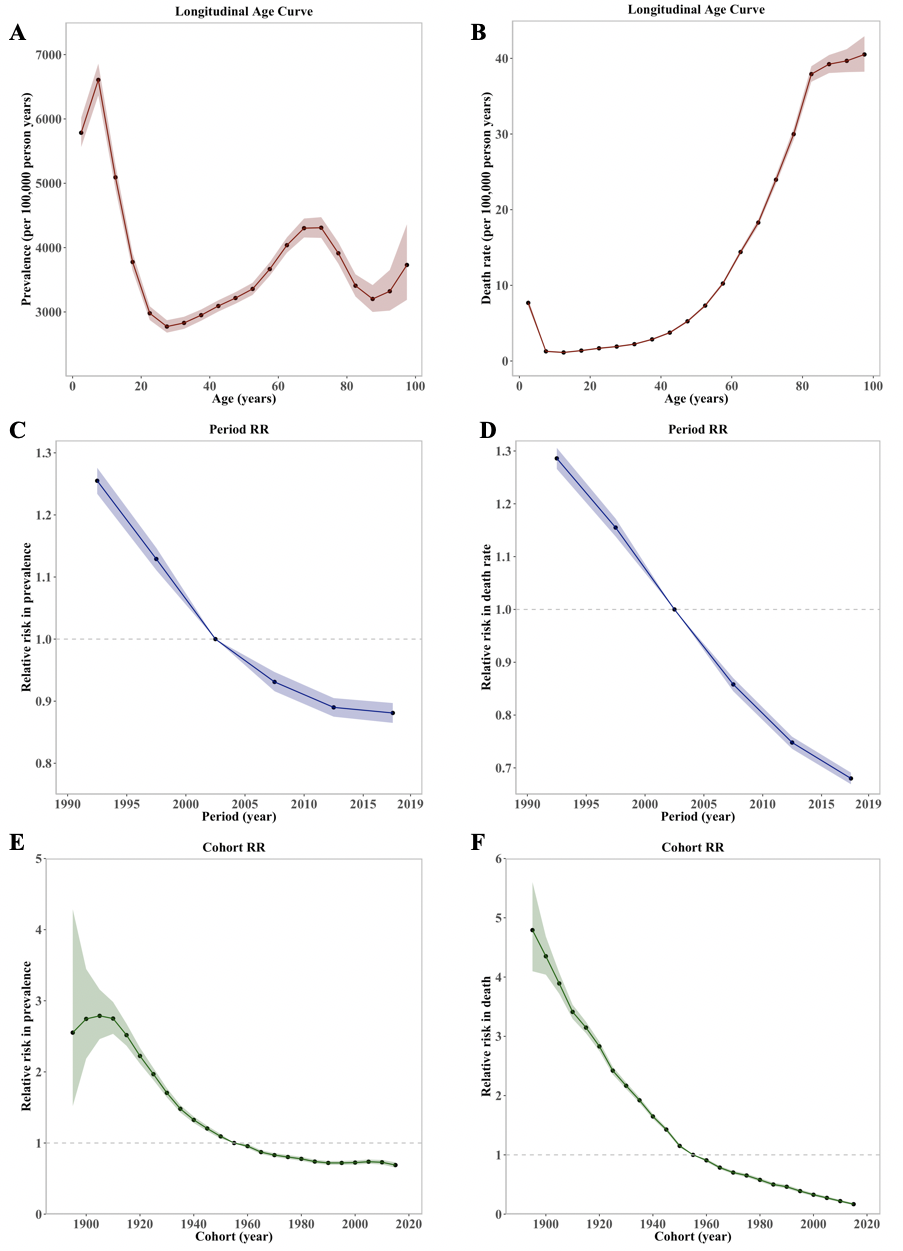
**Figure S14.** The Age-Period-Cohort analysis in prevalence and death rate of asthma; A: The age effect in the prevalence of asthma; B: The age effect in the death rate of asthma; C: The period effect in the prevalence of asthma; D: The period effect in the death rate of asthma; E: The cohort effect in the prevalence of asthma; F: The cohort effect in the death rate of asthma.


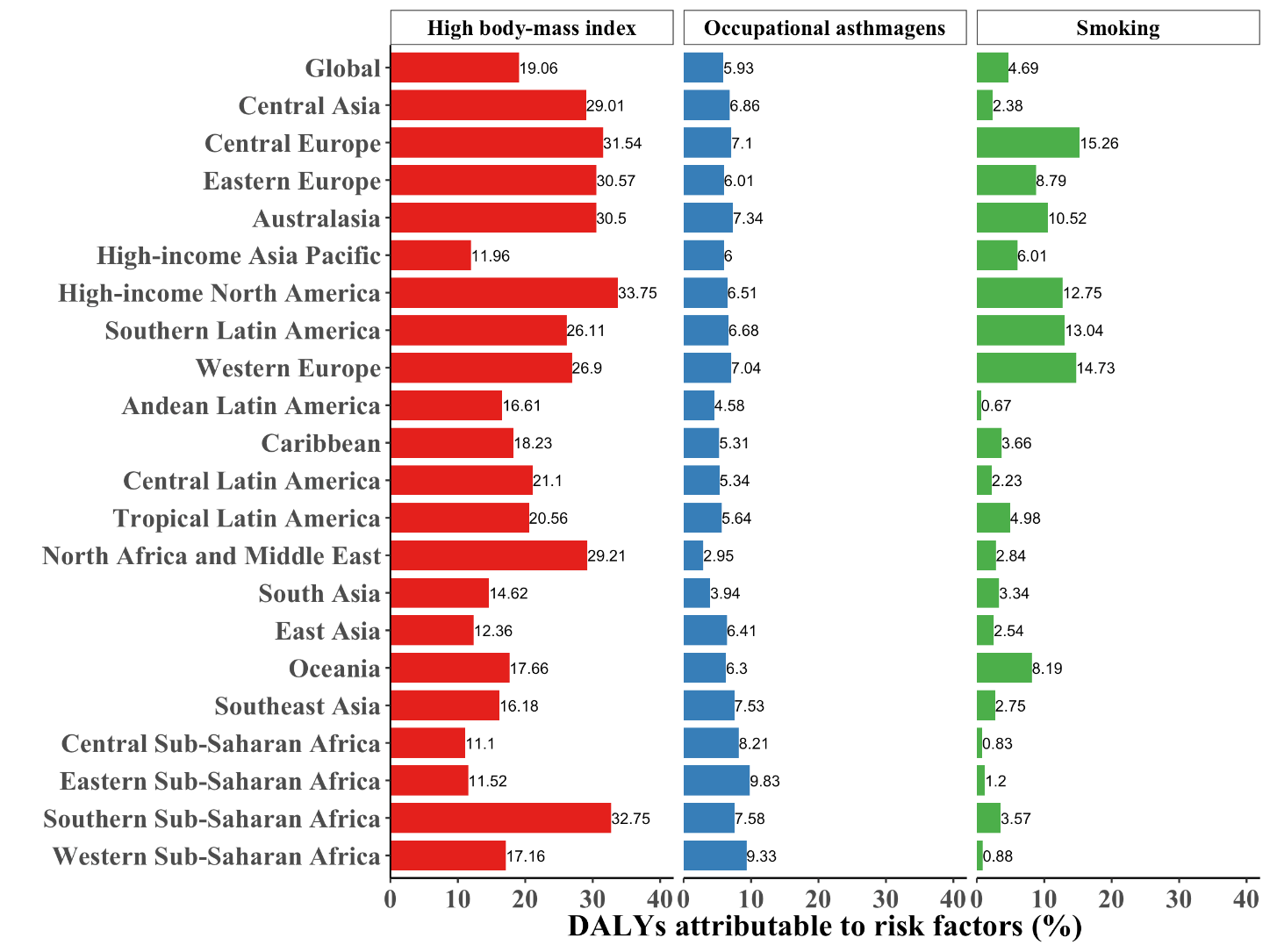


**Figure S15.** Percentage of DALYs due to asthma attributable to risk factors among females for 21 GBD regions in 2019. DALY=disability adjusted life years


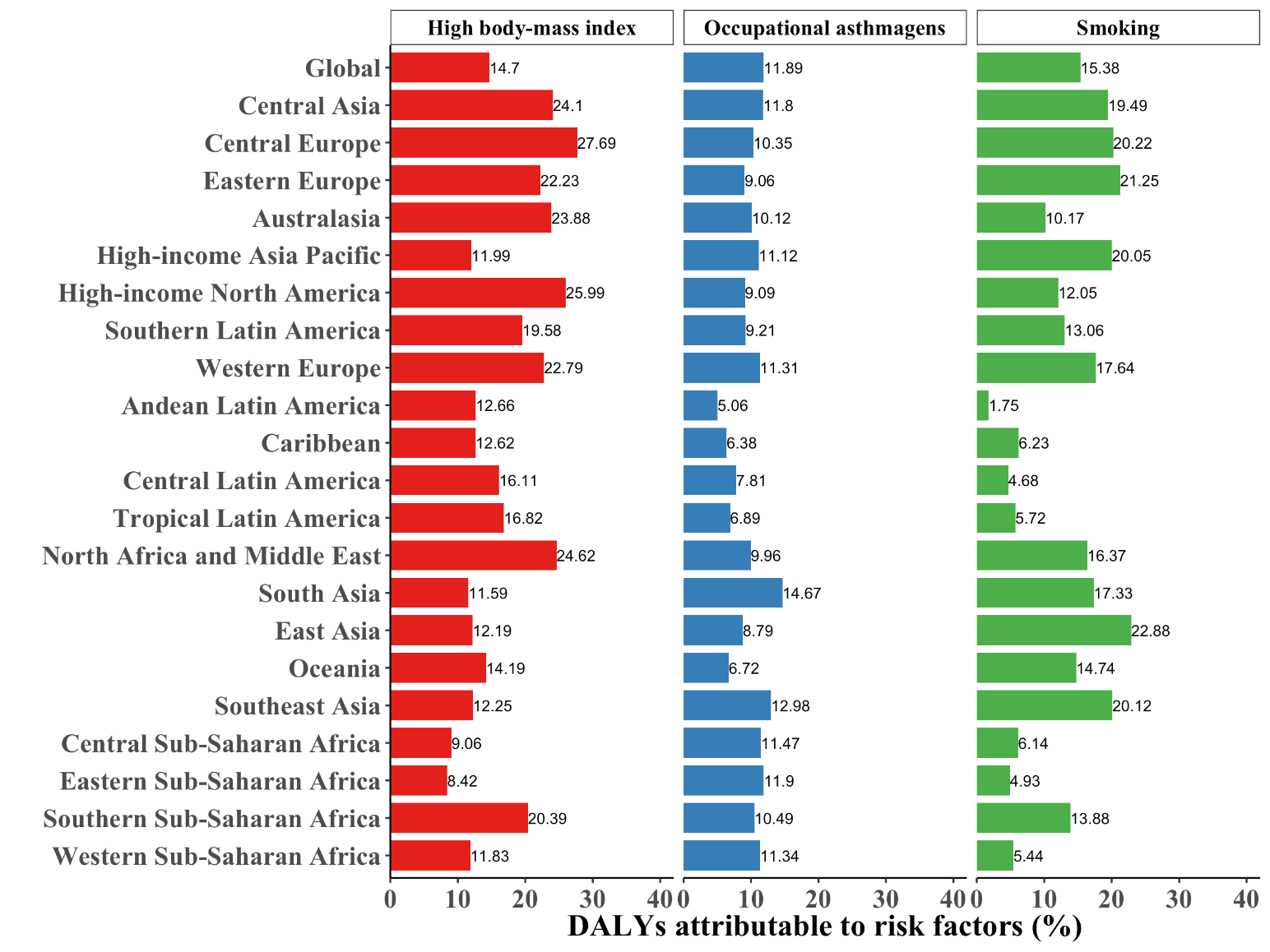


**Figure S16.** Percentage of DALYs due to asthma attributable to risk factors among males for 21 GBD regions in 2019. DALY=disability adjusted life years


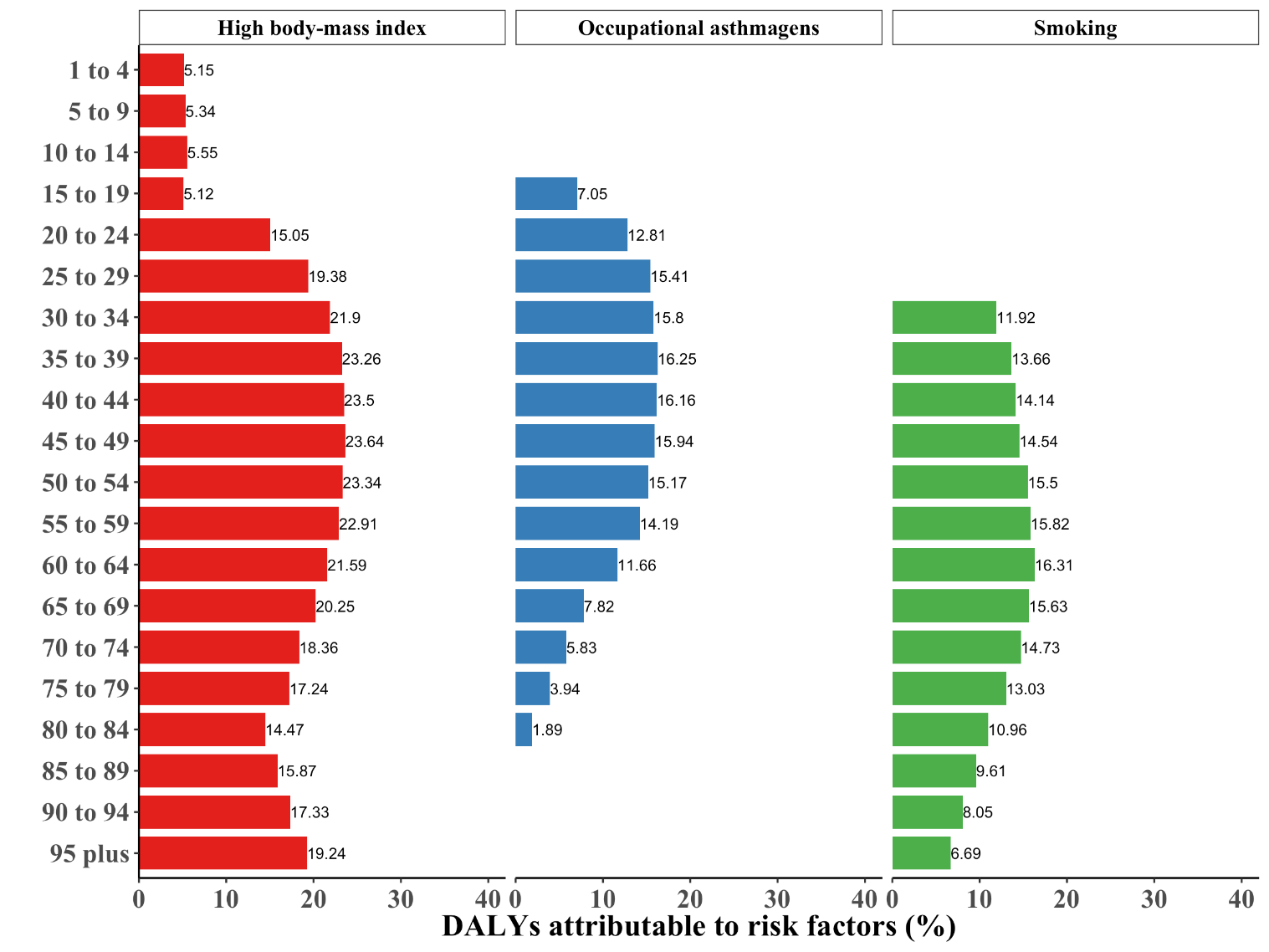


**Figure S17.** Percentage of DALYs due to asthma attributable to each risk factor, by age, in 2019. DALY=disability adjusted life years


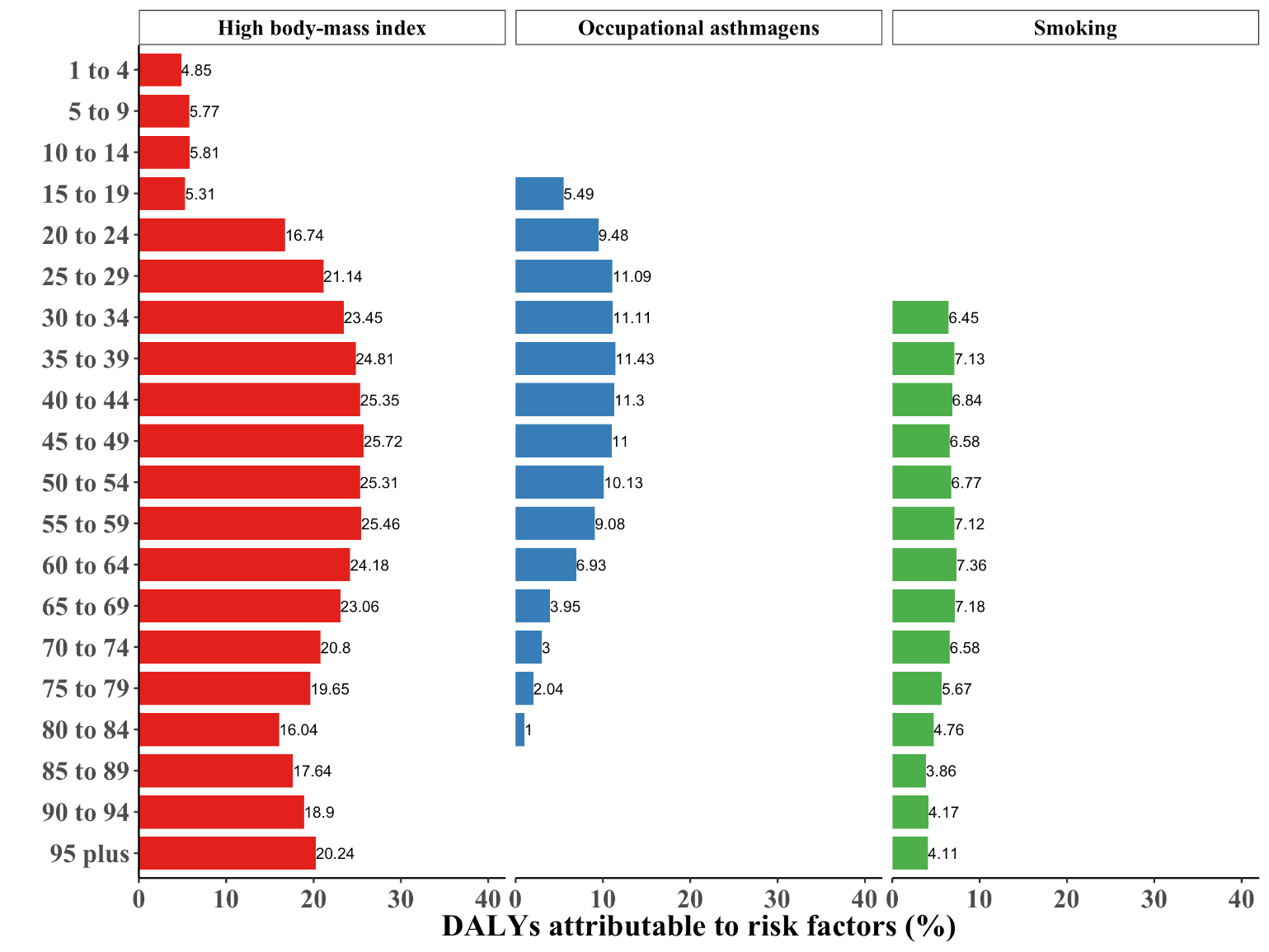


**Figure S18.** Percentage of DALYs due to asthma attributable to each risk factor among females, by age, in 2019. DALY=disability adjusted life years


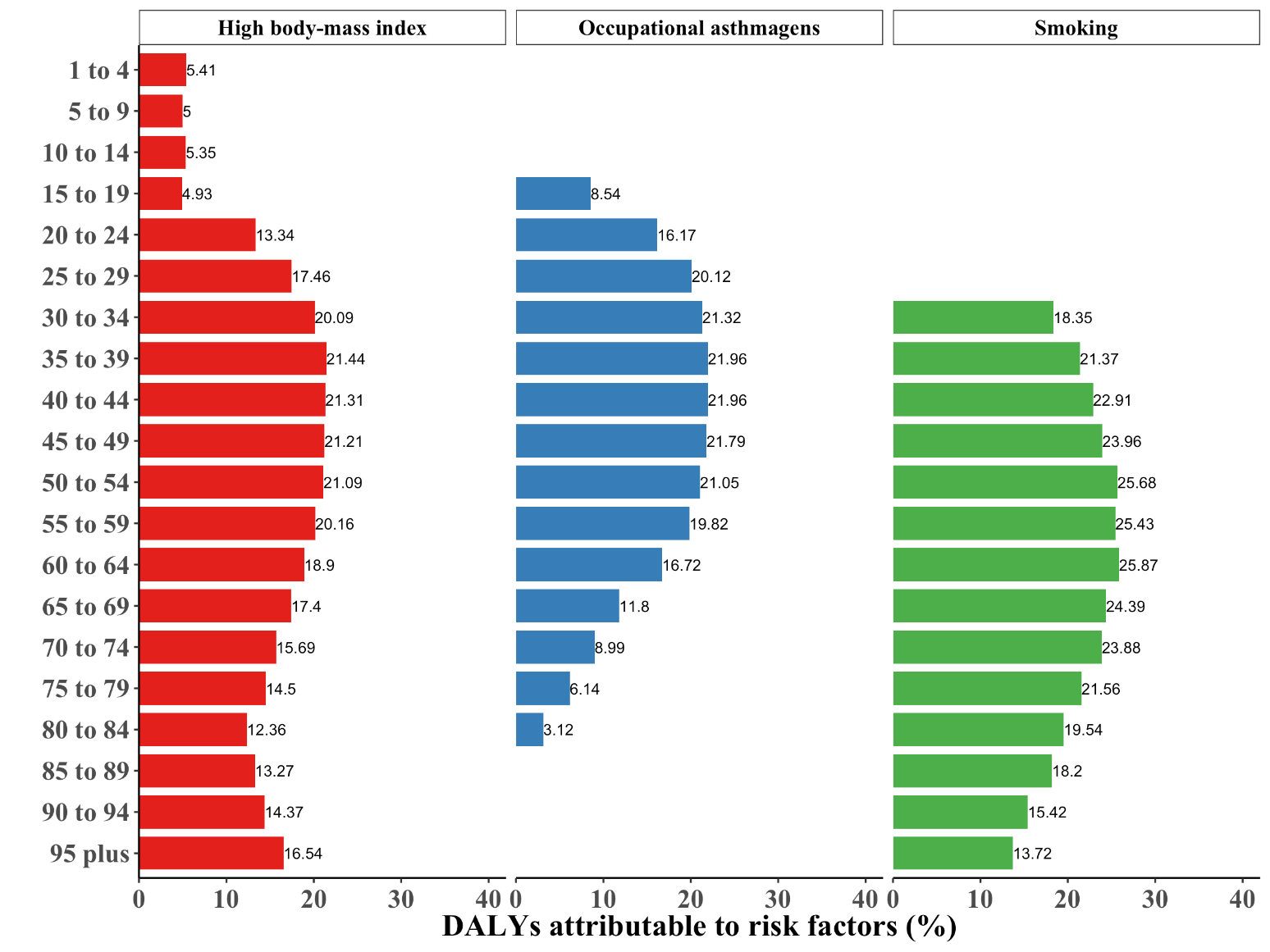


**Figure S19.** Percentage of DALYs due to asthma attributable to each risk factor among males, by age, in 2019. DALY=disability adjusted life years
